# Anthropogenic Factors and Social Organisation Drive Picobirnavirus Communities in Wild Rhesus Macaques

**DOI:** 10.1002/ece3.71727

**Published:** 2025-07-07

**Authors:** Krishna N. Balasubramaniam, Isamara Navarrete‐Macias, Shariful Islam, Heather L. Wells, Christopher Tubbs, Nistara Randhawa, Melinda K. Rostal, Karin E. Darpel, Daniel Horton, Jonathan H. Epstein, Ariful Islam, Simon J. Anthony

**Affiliations:** ^1^ School of Life Sciences, Faculty of Science and Engineering Anglia Ruskin University Cambridge UK; ^2^ Department of Population Health and Reproduction, School of Veterinary Medicine University of California at Davis Davis California USA; ^3^ Department of Pathology, Microbiology, and Immunology, School of Veterinary Medicine University of California at Davis Davis California USA; ^4^ Institute of Epidemiology Disease Control and Research (IEDCR) Dhaka Bangladesh; ^5^ EcoHealth Alliance New York New York USA; ^6^ Global Change Center Virginia Tech Blacksburg Virginia USA; ^7^ Department of Ecology, Evolution, and Environmental Biology Columbia University New York New York USA; ^8^ Reproductive Sciences, Conservation Science Wildlife Health San Diego Zoo Wildlife Alliance San Diego California USA; ^9^ One Health Institute, School of Veterinary Medicine University of California at Davis Davis California USA; ^10^ One Health Research Consulting LCC Glen Rock New Jersey USA; ^11^ The Pirbright Institute Pirbright UK; ^12^ Institute of Virology and Immunology IVI Mittelhäusern Switzerland; ^13^ University of Surrey School of Veterinary Medicine Guildford UK; ^14^ Animal and Plant Health Agency (APHA) Surrey UK; ^15^ One Health Science New York New York USA; ^16^ Gulbali Research Institute Charles Sturt University Wagga Wagga New South Wales Australia

**Keywords:** anthropogenic factors, joint species distributions models, microbial community ecology, picobirnaviruses, rhesus macaques, social network analysis, social organisation

## Abstract

Biologists are increasingly interested in the ecological and evolutionary factors that influence microbial communities. Yet compared to bacterial communities, our understanding of viral community ecology remains limited. Here, we investigated the factors influencing viral community composition and structure among wild rhesus macaques ( *Macaca mulatta* ) in human‐modified environments in Bangladesh, focusing on assemblages of picobirnaviruses (PbVs) as a model system. We found that anthropogenic factors—particularly human and livestock densities—were the strongest predictors of viral community composition. Host social organisation played a secondary role, shaping viral communities at the group and site levels. Virus–virus associations influenced co‐occurrence patterns primarily within individual hosts, but their effect became less evident at broader scales. In contrast, individual host traits (age, sex and physiological stress) and viral phylogenetic relationships had minimal effects on viral co‐occurrence. Notably, anthropogenic factors had opposing effects on different PbV taxa: While some viruses were more prevalent in areas with higher human and livestock densities, others were less common, possibly due to their association with wildlife or natural environments. This suggests that macaques may acquire certain PbVs from anthropogenic sources while experiencing reduced exposure to others in human‐modified landscapes. Together, these findings reveal the dominant role of environmental and social factors in shaping viral communities and highlight the hierarchical nature of virus community assembly—with different ecological processes operating at individual, group and site scales.

## Graphical abstract

We assessed the relative importance of host‐related, environmental and virus‐specific factors in influencing viral communities among wildlife hosts. Among wild rhesus macaques, we found that picobirnaviral communities were most strongly influenced by macaques' exposure to human impact, followed by their sociality and group living, and finally by virus–virus associations within hosts. Our study addresses critical gaps in our understanding of viral community ecology, adding to a growing perception of viruses as being integral components of host ecology and ecosystems.

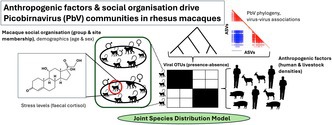

## 1 Introduction

As microbial communities are increasingly recognised as integral components of host and ecosystem functioning (French and Holmes 2020), understanding the processes that shape their composition and structure has become a central focus within microbial ecology. Research on *bacterial* communities has made significant progress towards this goal, revealing how community composition and structure are shaped by interactions among microbes, host traits and environmental conditions (e.g., Hibbing et al. 2010; Sun et al. 2016; Fackelmann et al. 2021; Zhu et al. 2021; Risely et al. 2022; Heni et al. 2023). In contrast, the ecology of *viral* communities—particularly those within wildlife hosts—remains comparatively underdeveloped. Only recently have researchers begun to examine the ecological and evolutionary forces that shape viral community assembly. Emerging studies have identified potential drivers of viral communities, including host and space‐use overlap (e.g., shorebirds and waterfowl: Wille et al. 2019; vampire bats: Bergner et al. 2019; cave bats: Sjodin et al. 2024), host demographics (e.g., vampire bats: Bergner et al. 2019; waterbirds: Hill et al. 2022), seasonal dynamics (e.g., rodents: Raghwani et al. 2023), environmental resource availability (e.g., livestock density for vampire bats: Bergner et al. 2019) and phylogenetic relationships of viral taxa (e.g., Anthony et al. 2015). Together, these studies contribute to a growing recognition that viruses are not merely agents of disease or spillover, but integral components of natural ecosystems, structured by multiscale interactions between hosts, microbes and their environments (French and Holmes 2020; Ladau and Eloe‐Fadrosh 2019; Prosser and Martiny 2020).

Despite growing interest in viral community ecology, empirical studies remain scarce. For instance, it is increasingly recognised that viral communities are structured hierarchically across biological levels—from cells to individuals, social groups, populations and ecosystems (French and Holmes 2020). However, we still know little about how these different scales shape viral communities within hosts. One important but underexplored aspect of this is the effect of host social organisation, defined here as the assortment of individual animals of varying age‐ and sex classes into stable social groups, and in turn of groups into populations (Clutton‐Brock 2016; Prox and Farine 2020), on viral communities. For instance, the social organisation of hosts has been shown to drive the transmission of both pathogenic and commensal bacteria, and thereby to shape bacterial communities in a variety of group‐living species (e.g., ungulates, nonhuman primates, social carnivores; reviewed in Sarkar et al. 2020). Yet, the role of social organisation in shaping viral community composition and structure remains largely unknown.

The impact of anthropogenic factors on viral communities in wildlife is also poorly understood. Human activities such as urbanisation and agricultural expansion are well‐documented drivers of cross‐species viral transmission (e.g., rodents: Epstein et al. 2020; meso‐carnivores: Bradley and Altizer 2007; wild boars: Triguero‐Ocaña et al. 2020; nonhuman primates: Engel and Jones‐Engel 2011). However, less is known about how these pressures affect the *composition* (i.e., which viruses are present) and *structure* (i.e., patterns of interactions and associations among viruses) of viral communities within hosts or populations. Indeed, most research on ecologically flexible wildlife species that overlap with humans and anthropogenic landscapes has focused on spillover risk and disease emergence (Grange et al. 2021; Fagre et al. 2022; Wolfe et al. 2007) often overlooking the broader ecological processes that govern viral community assembly and dynamics.

A third key factor likely shaping viral communities among wildlife is virus–virus interaction, yet this remains among the least studied. Viruses frequently co‐occur within hosts, and interactions such as competition, interference or facilitation can influence patterns of community assembly. While these processes are well documented in experimental systems (e.g., Delima et al. 2023; Sims et al. 2023; Bonavita et al. 2024), they are rarely incorporated into ecological models of viral diversity in wildlife (Anthony et al. 2015; French and Holmes 2020). Disentangling the effects of virus–virus associations from host and environmental influences requires analytical frameworks that can accommodate the complexity of multivariate data. Bayesian Joint Species Distribution Models (JSDMs; Ovaskainen et al. 2017; Tikhonov et al. 2020) offer a promising solution. Originally developed in macroecology to assess species co‐occurrence across landscapes, JSDMs are increasingly used in host–microbe systems to identify the relative contributions of host traits, environmental conditions and microbial interactions. Recent applications include analyses of pathogen coinfection in African lions ( *Panthera leo* ; Fountain‐Jones et al. 2019), parasite communities in rodents (Broecke et al. 2023) and gut microbiota in neotropical birds (Björk et al. 2018). These studies underscore the potential of JSDMs to quantify how interacting forces structure viral communities across biological and environmental scales.

To address these knowledge gaps, here we investigated the factors shaping viral community composition and structure in wild rhesus macaques ( *Macaca mulatta* ) living in human‐impacted environments in Bangladesh. Specifically, we evaluated how viral communities are influenced by (i) host exposure to anthropogenic factors in the environment, (ii) host‐specific characteristics related to social organisation, demographics and physiology and (iii) virus–virus associations. Rhesus macaques are particularly well‐suited for this research due to their complex social organisation which spans multiple hierarchical levels (individual animals → matrilines or subgroups within groups → groups → populations) and their frequent spatial overlap and social interactions with humans and livestock (Balasubramaniam et al. 2021; Jaman and Huffman 2013; Southwick and Siddiqi 1994). These interactions facilitate the bidirectional exchange of microbes with both humans and livestock (Engel and Jones‐Engel 2011; Balasubramaniam et al. 2020).

Within these macaque populations, we focused on picobirnaviruses (PbVs) as a model system for studying viral community ecology, given their abundance, widespread distribution and extensive taxonomic diversity across vertebrates, including macaques and other wildlife (Anthony et al. 2015; Duraisamy et al. 2018; Gallagher et al. 2017; Ganesh et al. 2014; Kuhar and Jamnikar‐Ciglenecki 2019; Raghwani et al. 2023). PbVs are gastrointestinal‐associated viruses likely transmitted through faecal–oral exposure, either via contaminated environmental sources (e.g., soil, faeces) or direct social contact with infected individuals (Ganesh et al. 2014; Raghwani et al. 2023). Although increasing evidence supports that PbVs infect bacteria (Krishnamurthy and Wang 2018; Wang 2022; Gan and Wang 2023), these associations remain embedded within vertebrate hosts and their social organisations and therefore represent a useful model with which to examine how viral communities are influenced by both environmental exposure and host social organisation.

Specifically, we investigated whether PbVs exhibit nonrandom patterns of co‐occurrence indicative of modular viral communities, and whether these patterns are shaped by viral phylogenetic relationships. We also examined whether macaques' social organisation influenced the similarity of viral community composition among individuals. Finally, we used Bayesian JSDMs to evaluate the relative contributions of multiple factors—including exposure to anthropogenic pressures (e.g., human and livestock densities), host demographics (e.g., age class and sex), physiological stress (e.g., cortisol levels) and virus–virus associations—in shaping PbV community composition and structure.

## 2 Materials and Methods

### 2.1 Sample Collection and Selection

**FIGURE 1:**
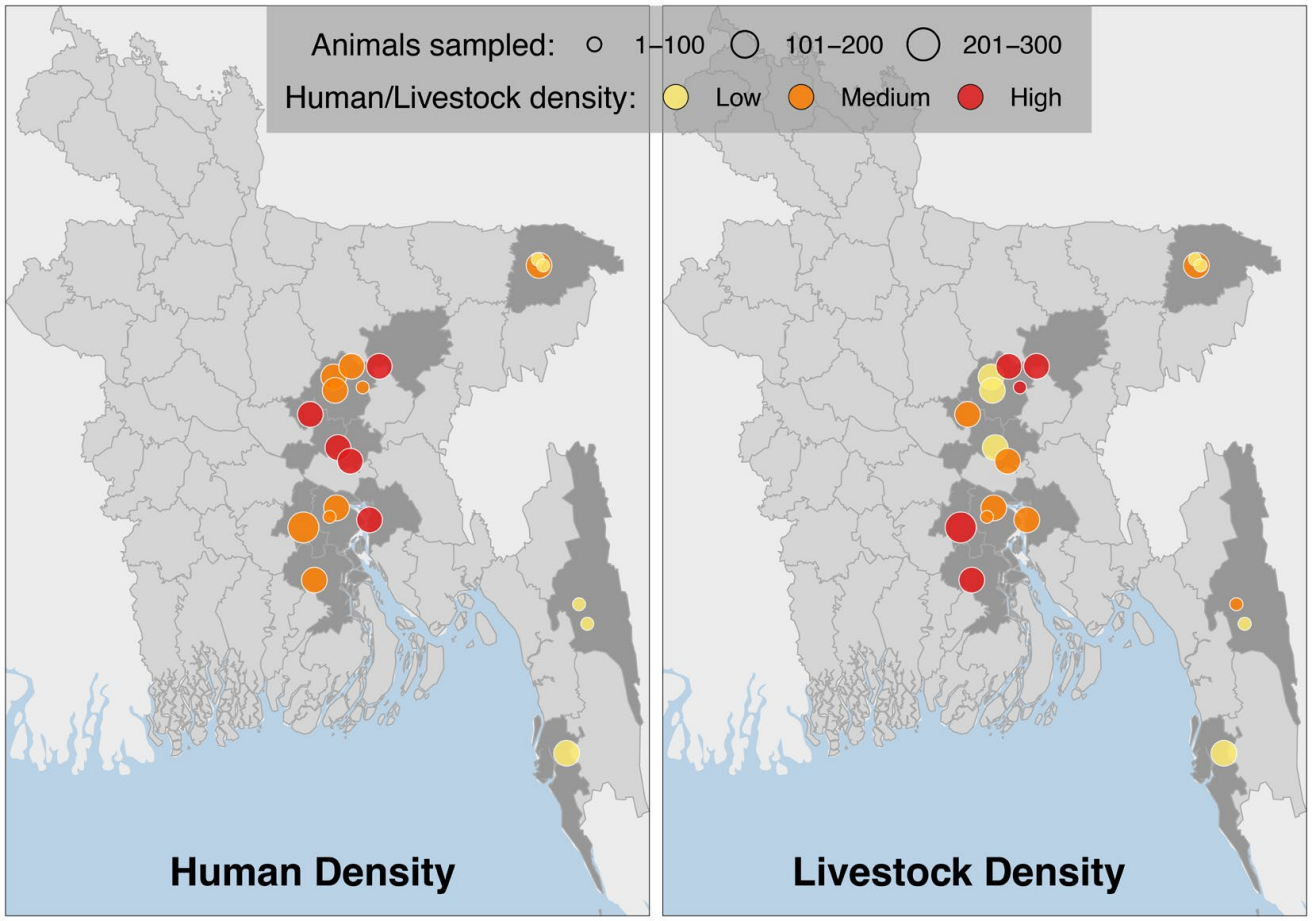
Location of 19 collection sites of rhesus macaques (sp. *Macaca mulatta* ) in Bangladesh, with indications of anthropogenic factors: Human and livestock density.

All sampling protocols were implemented following the procurement of ethical approval from the International Centre for Diarrhoeal Disease Research, Bangladesh (icddr, b; protocol: 2008‐074) and UC Davis (protocol: 16048). Faecal samples were collected noninvasively from 38 social groups of rhesus macaques across 19 locations or study sites in Bangladesh (Figure 1; Table S1). Sampling was conducted during two seasons: June–September 2015 (monsoon) and January–April 2016 (dry season). To facilitate defecation, macaques were provisioned with food items (e.g., fruits, bread), which they approached and retrieved voluntarily. All sampled populations were already well‐habituated to humans and regularly provisioned by local residents and, at some sites, tourists. Thus, provisioning was unlikely to alter their behaviour or affect the study outcomes. Furthermore, this study did not include a behavioural component and did not assess contact rates between individual macaques or between macaques and people, which might have been influenced somewhat by provisioning. All field team members wore appropriate PPE (full‐body suits, face‐masks, gloves, shoes) always while in the field, and so the risk of researcher‐to‐macaque viral acquisition was very minimal.

The field team chose study ‘sites’ as specific locations within Bangladesh chosen based on prior observations of rhesus macaque populations in the area, as well as varying degrees of human or livestock presence and overlap. Each site was unique and thereby separated from other sites by way of natural or anthropogenic barriers across varying distances (Figure 1; coordinates also provided in Table S1). Within each site, field team members sampled individuals from between 1 and 4 groups of macaques (Table S1). Macaques were designated as being part of the same ‘group’ based on the degree to which individual animals were observed to (i) rest or forage in close proximity to each other and (ii) show cohesivity and collective movement, that thereby clearly indicated that they formed a distinct ‘unit’ from other monkeys that belonged to a different group.

Following provisioning, field team members followed individual macaques to observe defecation, after which the specimen was collected within 10 min (Figure S1). Duplicate samples were placed into 2 mL cryovials and immediately frozen in a liquid nitrogen dry shipper. For each macaque group, a target sample size of 80% of individuals was set to ensure sufficient representation for PbV discovery. To achieve this, field teams revisited each site for three consecutive days, but only the most productive day (i.e., the day with the highest number of samples collected) was selected for analysis. In total, 8704 samples were collected and 2663 were subselected for further analysis. To minimise the chance of resampling the same individuals, the field team took several precautions. First, they agreed on the identification and distinction of macaque groups within a given location or site (see above). While sampling a group the observers (i) used physical features (e.g., facial recognition, body size and posture, genitals) to distinguish individual macaques both within and across age‐sex classes, (ii) adequately spaced sampling events to ensure the selection of different animals within a group for baiting, and (iii) on a given day moved to a different sampling group every 2–3 h to further minimise the likelihood of repeatedly sampling the same individuals since macaques seldom defecated more than once within a given 2–3 h sampling period per group per day.

### 2.2 Collection of Individual and Environmental Variables

Demographic data (sex and age class) were obtained through visual observation of individual macaques from whom faecal samples were collected. While demographic data were recorded for many sampled macaques, it was not always possible to determine sex or age class if individuals were too far away for accurate observation. When observed, sex was categorised as ‘female’ or ‘male’, and age class was categorised as ‘adult’ (defined as ≥ 4 years for males and ≥ 3 years for females: Rawlins and Kessler 1986) or ‘young’ (< 4 years for males and < 3 years for females: Rawlins and Kessler 1986) based on physically distinguishable features (Islam et al. 2022). Sex was recorded for 873 out of 2663 individuals, and age class was recorded for 993 out of 2663 individuals (Table S2).

To evaluate anthropogenic factors in their environment, we recorded human and livestock exposure levels for all 19 sites using two metrics: Human density impact (HDI) and livestock density impact (LDI). HDI and LDI classifications were based on repeated field observations conducted within the home ranges of macaque groups, using the average of at least three site visits, each involving counts by a minimum of two team members. No sites were recorded as entirely lacking human or livestock presence. For analytical purposes—and to reduce the influence of minor interobserver differences in count estimates—both HDI and LDI were categorised into three‐level ordinal variables. HDI was defined as ‘High’ (≥ 1000 humans present at a site), ‘Medium’ (100–999 humans) and ‘Low’ (10–99 humans). LDI was defined based on the abundance and diversity of livestock species, including goats, cattle, swine and poultry. Sites were categorised as ‘High’ (100–1000 individuals from two or more livestock species), ‘Medium’ (11–100 individuals of two or more species) and ‘Low’ (0–10 individuals of two or more species).

### 2.3 Total Nucleic Acid (TNA) Extraction

For each macaque, one faecal sample (*n* = 2663 samples) was selected for total nucleic acid (TNA) extraction. Samples were homogenised in 500 μL of Viral Transport Media (VTM) by vortexing for 1 min, followed by centrifugation at 8000× *g* for 5 min to pellet faecal debris. A 250 μL aliquot of the resulting supernatant was recovered and mixed with 250 μL of QIAamp lysis buffer (Qiagen). All extractions were performed under Biosafety Level 3 (BSL‐3) containment prior to sample inactivation, given the potential for zoonotic pathogens in macaques (Engel and Besnard 2017; Wolfe et al. 1998, 2007). TNAs were extracted using the QIAamp 96 Virus QIAcube HT kit (96‐well format) with protocol modifications specific to stool samples, including an additional wash step as recommended by the manufacturer.

### 2.4 Checking for PCR Inhibitors

Given the prevalence of PCR inhibitors in faecal samples (Oikarinen et al. 2009), a real‐time PCR assay targeting a synthetic exogenous DNA control was performed on all purified TNA samples to assess inhibition. The assay utilised the TaqMan Exogenous Internal Positive Control (EIC; Applied Biosystems) with TaqMan Universal PCR Master Mix (Applied Biosystems). Reaction conditions were as follows: 50°C for 2 min, 95°C for 10 min, followed by 45 cycles of 95°C for 15 s and 60°C for 1 min. The total reaction volume was 20 μL, which included 2 μL of purified macaque faecal TNA. PCR inhibition was determined by deviations in the expected cycle threshold (Ct) values of the exogenous control.

### 2.5 Detection of PbVs

The workflow and molecular results leading up to PbV detection and confirmation are outlined in Figure S2. Following confirmation that samples did not contain significant PCR inhibitors, all 2663 samples were assayed using a consensus PCR (cPCR) approach for the broad detection of PbVs (Anthony et al. 2015). The assay was performed using the One‐Step RT‐PCR kit (Qiagen). A total of 5 μL of TNA was first denatured at 95°C for 3 min and then transferred to ice. A master mix containing primers, enzymes, dNTPs and buffers was prepared according to the manufacturer's instructions. RT‐PCR cycling conditions included an initial reverse transcription stage at 50°C for 30 min, followed by enzyme activation at 95°C for 20 min. Amplification was carried out for 40 cycles of 96°C for 5 s, 48°C for 8 s and 68°C for 26 s. The cycling protocol concluded with a final extension at 72°C for 4 min, after which the reaction was held at 4°C. Primer sequences used in this assay are reported in Anthony et al. (2015). PCR reactions were run on 1% agarose gels, and bands of the expected size (600–800 bp) were excised. Presumptive positive bands were purified using Ultrafree‐DA centrifugal filters (Millipore #42600). A total of 2 μL of purified PCR product was then cloned into the Strataclone PCR cloning vector (Agilent Technologies) following the manufacturer's instructions. Colonies were selected via blue and white screening with X‐gal, and for each sample, 18 white colonies were picked and sent for traditional Sanger sequencing using the M13 reverse primer (Genewiz).

### 2.6 Sequence Data Cleaning

All raw Sanger sequences were trimmed to remove vector sequences and primer‐binding regions using Geneious software (Version 9.1). Sequences shorter than 300 nucleotides were excluded, as they were typically of poor quality, non‐PbV origin (e.g., cross‐reactions), or derived from PbVs but insufficiently long for robust phylogenetic analyses. Raw chromatograms of the remaining sequences were manually reviewed to trim low‐quality termini or remove low‐quality regions entirely. Sequences that passed quality assessment were verified using the Basic Local Alignment Search Tool (BLAST, NCBI). Both nucleotide BLAST (blastn) and translated nucleotide‐protein BLAST (blastx) were performed against the complete PbV GenBank database. Any sequences that did not match PbVs were excluded. The correct orientation was confirmed for all high‐quality PbV sequences, which were then used for operational taxonomic unit (OTU) classification and further analyses.

### 2.7 Classification of Viral Operational Taxonomic Units (OTUs)

A matrix of pairwise sequence identities for all possible combinations of sequence pairs was generated using global alignment with the ape package in R (Padaris and Schliep 2019). In this approach, each sequence was independently aligned against every other sequence to create a matrix of percent identities. The frequency of Percent Identity Distances (PIDs) was then plotted as a histogram to identify troughs and peaks, with troughs used to define cut‐off values for demarcating sequences into viral OTUs (Maes et al. 2009; Sjodin et al. 2024). Multiple troughs were observed in the histogram (see Results), prompting the application of additional criteria to select the appropriate cut‐off point. To select the appropriate cut‐off(s), we applied an evolutionarily informed definition of an OTU, reasoning that (i) an OTU should reflect the smallest evolutionary unit, aligning with the use of ‘species’ in macroecology, and (ii) it should not exhibit evidence of multiple ecological or evolutionary processes within, as such heterogeneity would confound analyses of community assembly. Sequences were assigned to OTUs at various cut‐off points (based on troughs in the histogram) and then evaluated phylogenetically to ensure that sequences formed a radial distribution with no evidence of subclustering. Subclustering of sequences separated by long branch lengths could indicate the presence of multiple discrete ecological or evolutionary processes. Consequently, OTUs were defined based on the cut‐off that produced radial phylogenetic distributions of sequences.

### 2.8 Faecal Cortisol

Faecal cortisol levels were analysed for a subset of macaques (1047 out of 2663 samples) using a standardised radioimmunoassay (RIA) protocol developed by the Endocrine Laboratory of the San Diego Zoo Wildlife Alliance. Approximately 0.1 g of lyophilised faeces was resuspended in 5 mL of 90% ethanol, vortexed for 2 min and gently boiled at 70°C for 20 min. Samples were then centrifuged at 1000× *g* for 10 min at room temperature, and the supernatant was collected into a fresh tube. The faecal pellet was resuspended in an additional 5 mL of 90% ethanol, vortexed for 20 min and centrifuged again (1000× *g* for 10 min at room temperature). The resulting second supernatant was combined with the first and dried under air before being reconstituted in 1 mL of 90% ethanol and stored at −20°C until assayed. Faecal cortisol concentrations (ng/g) were measured in duplicate using a radioimmunoassay. Briefly, 0.1 mL of faecal extract was incubated in phosphate‐buffered saline (PBS, 0.5 mL, pH 7.0) with ~10,000 cpm/100 μL of ^3^H‐cortisol tracer (PerkinElmer, NET396) and a rabbit anticortisol antibody (1:3000 dilution, MP Biomedicals, #07‐121016). Samples were incubated overnight at 4°C. Following incubation, bound hormone was separated from unbound hormone using 0.5% dextran‐coated charcoal and centrifugation at 1000× *g* for 15 min at 4°C. For each assay, cortisol standards (7.8–1000 pg/50 μL) were run in duplicate and used to generate a standard curve, which was then used to extrapolate cortisol concentrations in unknown samples.

### 2.9 Data Analyses

All data analyses were conducted based on unique PbV OTU assignments at a genetic similarity cut‐off of ≥ 96%. To assess the robustness of our findings to OTU similarity criteria, sensitivity analyses were performed using an alternative cut‐off of ≥ 88%. These estimates were based on the detection of suitable peaks and troughs while plotting the distributions of the PID values between sequences (see Results for more details). The results of these additional analyses are presented either in the main Results section or as supporting information. All statistical tests were two‐tailed, with a significance threshold set at *p* < 0.05.

To determine whether PbVs showed nonrandom patterns of virus–virus co‐occurrence that underlay their community structure, we used network analysis (as in Anthony et al. 2015; Risely et al. 2022; reviewed in Fountain‐Jones et al. 2019). We constructed three unipartite viral co‐occurrence networks, one for each macaque social organisational level of individual animals within macaque groups within site‐specific populations. That is, nodes in these networks were viral OTUs, and edges were the number of (i) individual macaques (*n* = 2664), (ii) macaque groups (*n* = 38) and (iii) sites (*n* = 19). To determine whether viral OTUs showed evidence for subgrouping, we calculated the community modularity (Newman 2006) of each network, using the Walktrap *y* algorithm (Pons and Latapy 2005) in the *Igraph* R package (Csardi and Nepusz 2006). Modularity indicates the extent to which a network shows subgrouping of nodes, that is, sets of nodes that are more well‐connected to each other than they are to other nodes, and can range between 0 and 1 (Newman 2006). Higher modularity would indicate greater evidence for viral subgrouping, that is, for OTUs to cluster together by co‐occurring within the same individual, group or site. To determine whether observed modularity was greater than expected by chance, we used a prenetwork randomisation test (Farine 2017). Holding the number of nodes and edges constant, these tests compared the observed modularity scores to a distribution of scores calculated from 10,000 permuted networks generated by randomly swapping the edges of the original network (Farine 2017).

To test whether viral co‐occurrence and community structure were influenced by their phylogenetic relationships, we used univariate Matrix Regression using a Quadratic Assignment Procedure (or MR‐QAP) test (Dekker et al. 2007), using the *sna* R package (Butts 2016). In this analysis, the outcome matrix was the virus–virus unipartite co‐occurrence network, in which the cells or edge‐weights represented the number of macaques in which pairs of OTUs co‐occurred. The outcome matrix was then regressed against a predictor matrix containing branch length information (ultrametric phylogenetic distances) between pairs of OTUs.

To determine whether macaques' social organisation influenced viral communities, we calculated coefficients of pairwise Beta‐(*β*) diversity dissimilarity (e.g., Anthony et al. 2015) in their viral community compositions between pairs of macaque hosts using the *betapart* R package (Baselga et al. 2021). This approach converted bipartite matrices or networks of the occurrence of OTUs (columns) by individual macaques (rows) or by groups (rows), into unipartite matrices in which the rows and columns are macaques (individuals, groups), and in which the cells contain continuously distributed likelihood scores of the extent of viral dissimilarity between macaques. We then used a logistic MR‐QAP to regress the pairwise *β*‐diversity dissimilarity coefficients against macaques' group membership scored as pairs of macaques occurring in the same social group scored as ‘1’ vs. in different social groups scored as a ‘0’. To then determine whether viral communities were more similar among macaques within the same site compared to those at different sites, we ran a second MR‐QAP. In this we regressed a matrix of pairwise viral *β*‐diversity dissimilarity calculated between each pair of macaque groups, against a matrix in which the cells contained measures of the intersite geographic distances between pairs of macaque groups with ‘0’ values being entered for the distance between groups that occurred within the same sites.

To assess the relative effects of macaque characteristics, anthropogenic factor and virus–virus associations on viral communities, we used JSDMs. Specifically, we fit two JSDMs for two scales of macaque social organisation—one at the scale of individual macaques within groups and another at the scale of macaque groups within sites. We restricted the JSDM analyses to a subset of 264 macaques for whom we were able to collect data on all anthropogenic and animal‐specific variables (exposure to humans, exposure to livestock, age, sex *and* cortisol concentrations) assessed in the study. Such a subsetted covariate dataset was a tradeoff against our prioritising greater depth and geographic spread of faecal sample collection. Despite this restriction, we were assured in our results reflecting broader ecological and evolutionary patterns given that the 264 macaques were evenly distributed across the 38 groups and 19 sites that we sampled, and as such represented a meaningful sample size for these analyses (Fountain‐Jones et al. 2019). Moreover, we refrained from running multiple univariate analyses for some covariates (e.g., human and livestock densities) that were more broadly represented across the dataset compared to others, since JSDMs are multivariate analyses that test the relative importance of multiple covariates on the distribution of taxonomic entities (Ovaskainen et al. 2017).

In the first JSDM, we included a bipartite matrix, in which rows were a subset of 264 macaques for whom data on all animal and environmental covariates were available (age, sex, and cortisol: see above). As columns, we included a subset of 42 viral OTUs (39 at 88% similarity) that occurred within at least two macaques. OTUs that occurred in just one macaque were excluded from this analysis (Fountain‐Jones et al. 2019). The cells contained binary information on the presence (1) or absence (0) of each OTU in the row animal. As covariates, we included macaques' demographic characteristics (animals' age, sex), physiological factors (faecal cortisol concentrations, in nanograms per microlitre of faeces: see Supporting Information), and anthropogenic factors (human and livestock densities) as fixed effects. Macaques' group ID was included as a random effect. In the second JSDM, the bipartite matrix contained 38 macaque groups as rows, and 104 viral OTUs that were each found in two or more macaque groups as columns. We included macaque group size, human density and livestock density all as main effects, and site ID as a random effect. To assess the degree to which OTU evolutionary relationships impacted virus–virus co‐occurrence within macaques, we included an ultrametric tree of inter‐OTU phylogenetic distances in both JSDMs. We fitted both models with Bayesian inference criterion with a probit link function using the *HMSC* package in R (Blanchet et al. 2018). We utilised the default priors, and ran two chains of 10,500 iterations each, of which we removed the first 500 iterations as burn‐in and thinned the remaining ones to give 5000 posterior samples per chain, per model. We used visual inspections of MCMC traces and calculations of the Gelman‐Rubin diagnostics to assess model convergence.

## 3 Results

### 3.1 PbV Occurrence Among Macaques

**FIGURE 2:**
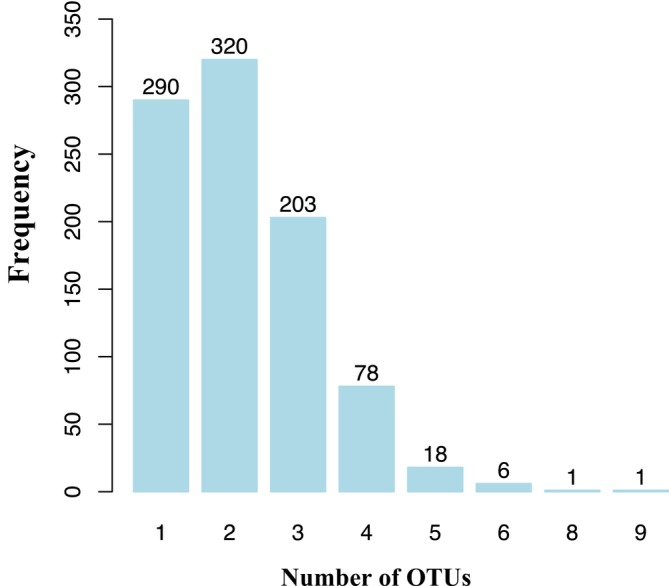
Summary of OTU co‐occurrence. The numbers of OTUs found in individual macaques ranged from 1 to 9 (*X* axis = number of OTUs). Frequency indicated on the *y*‐axis.

cPCR analysis yielded 11,303 PbV sequences from 917 individual macaques, representing 34% of the 2663 individuals sampled. The high number of sequences relative to the number of positive macaques reflects the sequencing methodology: for each positive macaque, a single cPCR band was cloned, and 18 colonies were sequenced. Depending on the sample, these sequences represented either 18 identical copies of the same viral strain or a mix of distinct PbV strains, capturing both biological diversity and potential redundancy within each cPCR band. In some cases, not all 18 sequences represented viral origin. The number of unique PbV OTUs detected in individual macaques ranged from 1 to 9 (Figure 2). Specifically, 230 individuals had 1 OTU, 320 individuals had 2 OTUs, 203 individuals had 3 OTUs, 78 individuals had 4 OTUs, 18 individuals had 5 OTUs, 6 individuals had 6 OTUs, 1 individual had 8 OTUs, and 1 individual had 9 OTUs (Figure 2).

### 3.2 OTU Classifications and Assignments

A histogram of PID values was generated from the 11,303 × 11,303 PID matrix (Figure S3), revealing several peaks and troughs that could be used as cut‐offs to define OTUs. Notable troughs were observed at 96% (highest taxonomic resolution) and 88% PID. When OTUs were defined using a ≥ 96% PID cut‐off, almost all OTUs displayed a radial phylogenetic structure (Figure S4), consistent with homogeneity in evolutionary processes within each OTU and the absence of confounding subclustering. In contrast, defining OTUs with a ≥ 88% PID cut‐off revealed significant subclustering, indicating potential ecological or evolutionary heterogeneity within OTUs (Figure S5). Based on these findings, the ≥ 96% PID cut‐off was selected, resulting in the identification of 215 OTUs.

### 3.3 PbV Community Structure

**FIGURE 3:**
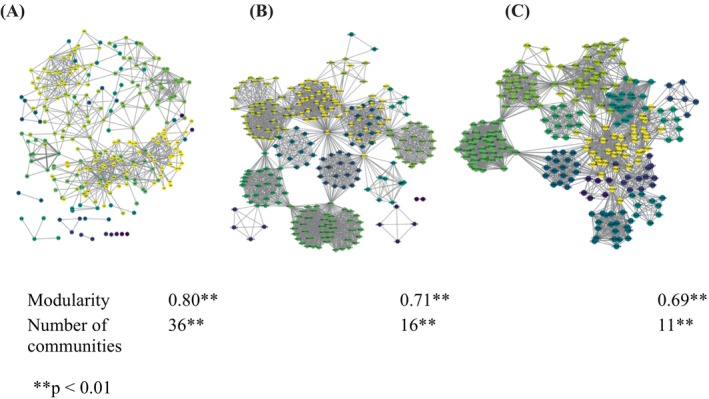
Viral OTU co‐occurrence networks constructed at different social organisational levels. Nodes represent uniquely identified OTUs of PbVs. Nodes of the same color indicate OTUs that were part of the same community, as identified through the Walktrap algorithm. Edges represent the numbers of (A) individual monkeys, (B) groups and (C) populations within sites from which each pair of OTUs were jointly isolated from. Values under each network indicate the corresponding network modularity and the number of communities.

Our network analysis revealed that unipartite viral co‐occurrence networks exhibited high modularity at all three levels of host social organisation, with clearly distinguishable clusters of OTUs that were more likely to co‐occur with each other than with other OTUs (Figure 3A–C; Table S3). Prenetwork randomisation tests (Farine 2017) further confirmed that, at all three organisational levels, both the modularity and the number of distinct OTU clusters detected within co‐occurrence networks were significantly greater than expected by chance, given the network size and total number of network connections (Figure 3A–C; Table S3).

### 3.4 Effects of Evolutionary and Ecological Factors on Viral Communities

The MR‐QAP test assessing the effects of viral phylogenetic distances on the co‐occurrence network revealed no significant relationship (*β* = 0.02, *p* = 0.83; for > 88% cut‐off: *β* = −0.01, *p* = 0.88). In other words, neither closely related nor distantly related viruses tended to co‐occur within the same host macaque. However, other MR‐QAP tests showed a strong effect of macaques' social organisation, specifically their group and population membership, on viral β‐diversity. Specifically, macaques from the same groups showed significantly lower degrees of viral dissimilarity, or conversely higher degrees of viral similarity, than macaques from different groups (viral β‐diversity dissimilarity ~ group membership: *β* = −1.75, *p* < 0.01; for > 88% cut‐off: *β* = −1.32, *p* < 0.01). Furthermore, there was a significant increase in viral β‐diversity dissimilarity with inter‐site geographic distance (*β* = 2.93, *p* < 0.01; for > 88% cutoff: *β* = 3.03, *p* < 0.01), that is, macaques within more closely located or the same sites showed a higher viral similarity compared to macaques across more distantly located sites.

**FIGURE 4:**
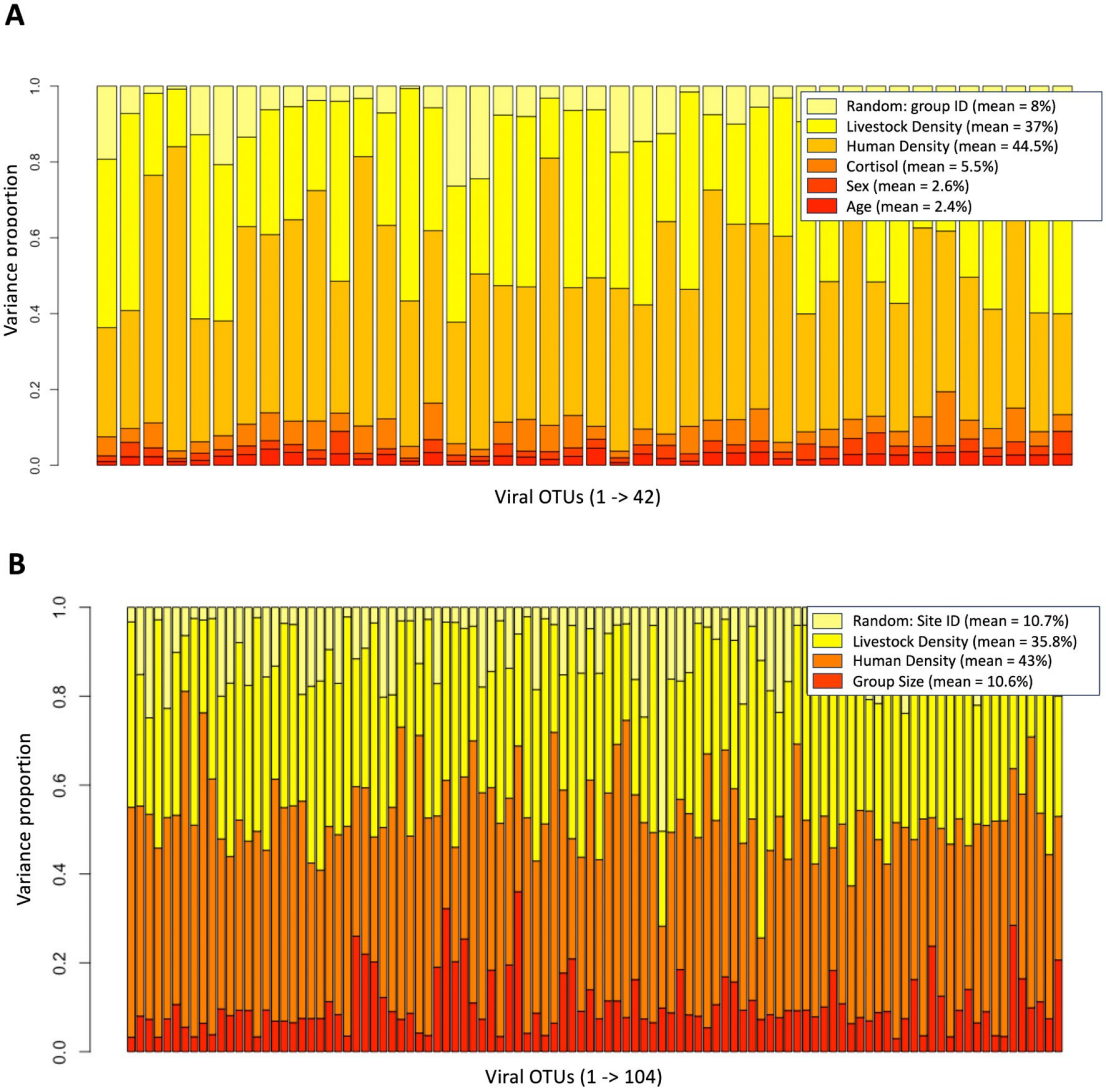
Plots of variance partitioning of the effects of animal and environmental covariates on PbV OTU communities, from JSDMs of (A) individual macaques within groups, and (B) groups within species.

**FIGURE 5:**
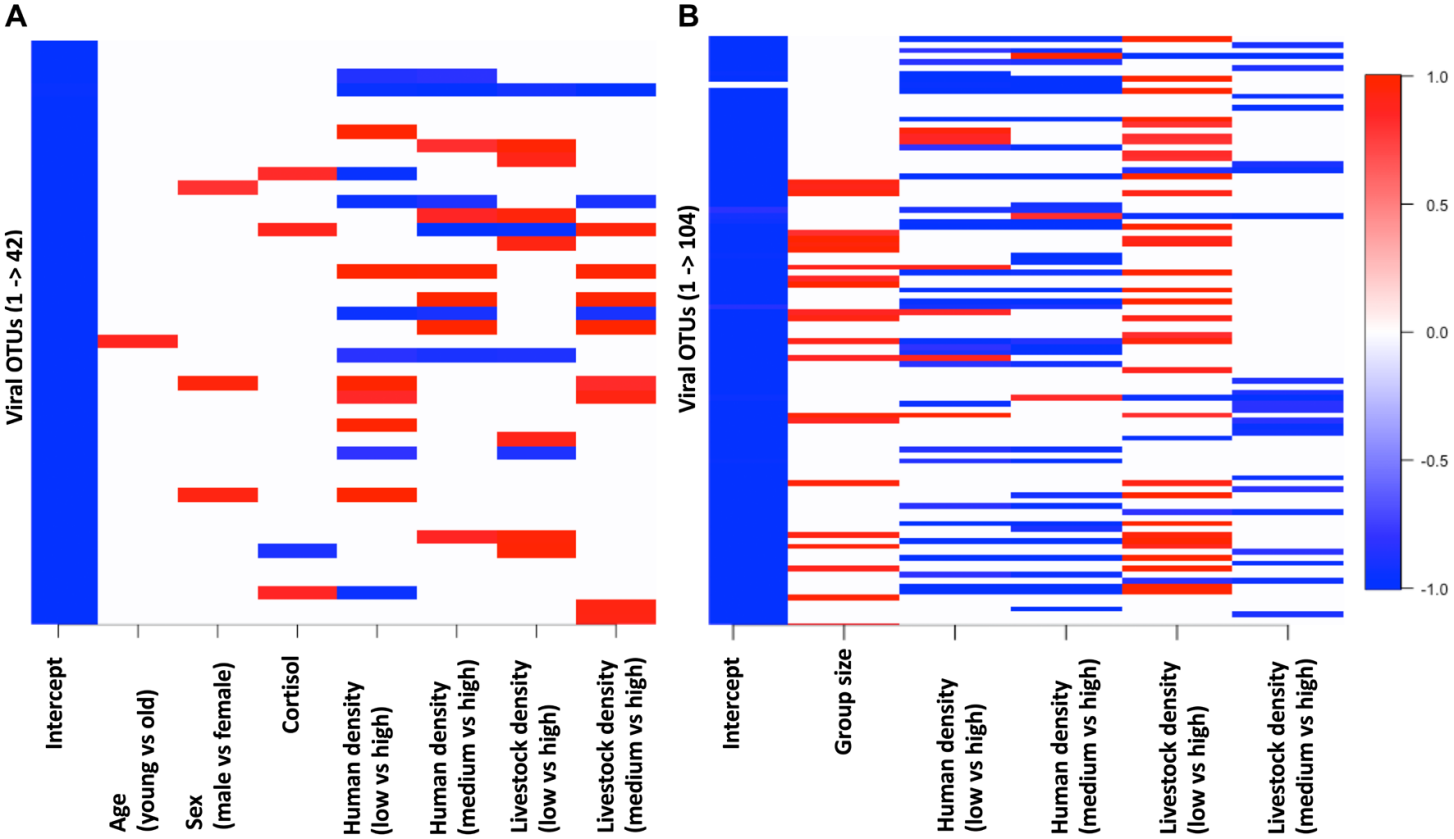
Heat maps of posterior support values from the JSDMs examining the effects of animal and environmental covariates on viral OTU communities at (A) the individual level with group as a random effect and (B) the group level with site as a random effect. Rows contain the covariates, and the columns PbV OTUs. Cells indicate both strongly positive (red) and strongly negative (blue) effects at > 95% posterior support, particularly for anthropogenic factors of human and livestock densities. Blank cells indicate no strong effects.

JSDMs to evaluate the relative effects of anthropogenic factors, macaque‐specific characteristics and virus–virus associations on viral communities revealed that exposure to anthropogenic factors in the environment—specifically HDI and LDI—had the strongest influence on OTU communities within macaques. At the individual level, HDI and LDI together accounted for an average of 81.5% of the variance in OTU occurrence (37% contribution from HDI + 44.5% from LDI; Figure 4A). Similarly, at the group level, HDI and LDI accounted for 78.8% of the variance (43% from HDI + 35.8% from LDI; Figure 4B) in OTU occurrence. Interestingly, HDI and LDI effects were nonlinear: While they significantly increased the likelihood of occurrence of some OTUs, they also decreased the likelihood of occurrence of others (Figure 5A,B). In comparison, the effects of macaques' social organisation, specifically their group membership (8% of the variance; Figure 4A) and site membership (10.7% of the variance; Figure 4B) which were included as random effects, and their group size (10.6% of the variance; Figure 4B), were weaker. Finally, macaques' demographic characteristics, specifically their age class (2.4%) and sex (2.6%; Figure 4A), and physiological stress levels measured via faecal cortisol (5.5%; Figure 4A) had the weakest effects on the likelihoods of OTU occurrence.

**FIGURE 6:**
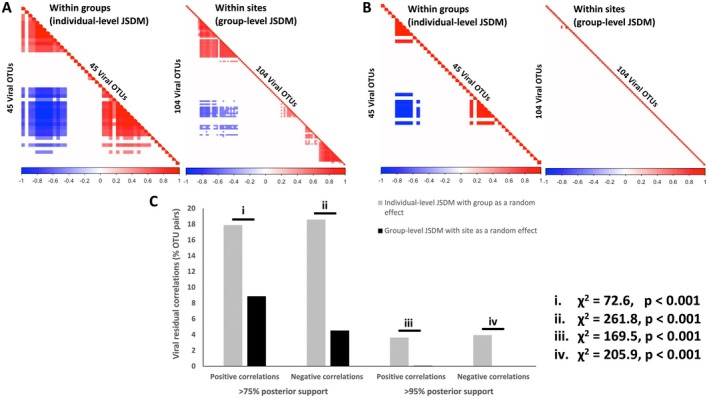
Heat maps that indicate correlations between viral OTU pairs calculated as the residual values from individual‐level JSDMs with group as a random effect and from group‐level JSDMs with site as a random effect, for posterior support cut‐offs of (A) 75% and (B) 95%. Cells indicate both strongly positive (red) and strongly negative (blue) correlations between OTU pairs after accounting for phylogenetic relationships, and for animal and environmental covariates. White cells indicate no strong correlations. In each heat map, OTUs have been reordered to best illustrate the observed association structure. (C) Bar plots and proportions‐test results comparing the percentages of viral OTU pairs that showed strong (both positive and negative) correlations across the organisational levels, that is, individuals within groups, versus groups within sites.

In both JSDMs, we found no evidence of phylogenetic signals in viral communities—that is, closely related OTUs were not more likely to co‐occur within the same macaques. This was consistent across both individual‐level (*ρ* = 0.20, 95% confidence interval: 0–0.74) and group‐level models (*ρ* = 0.33, 95% confidence interval: 0–0.73). However, the JSDMs identified several strong virus–virus associations among OTUs that appeared to influence viral community composition, even after accounting for the effects of anthropogenic factors, host‐specific social organisation and biology and viral phylogenetic relationships. Notably, these virus–virus associations were more pronounced at lower organisational levels (i.e., among individual animals within macaque groups) compared to higher levels (i.e., among macaque groups within sites). Figure 6 highlights OTU pairs that exhibited strongly positive or negative correlations, with posterior support values exceeding > 75% (Figure 6A) and > 95% (Figure 6B), respectively. Chi‐squared proportions tests confirmed that significantly greater percentages of OTU pairs showed strongly positive or strongly negative correlations at the individual level compared with the group level (Figure 6). In other words, this test confirmed that the contribution of virus–virus associations to shaping viral community composition was more discernible at the level of individual animals than at the level of social groups.

All results remained consistent when we repeated the JSDMs using OTU assignments based on a similarity cut‐off of > 88% (Figures S6A,B, S7A,B and S8A–C).

## 4 Discussion

Our findings reveal that anthropogenic factors—especially human and livestock densities—are the dominant drivers of PbV community composition and structure in rhesus macaques. In contrast, host social organisation plays a secondary role, shaping viral communities at the group level, while virus–virus associations are most apparent within individual hosts. Despite the presence of strong viral co‐occurrence patterns, we found little evidence that phylogenetic relatedness among PbVs influenced their distribution, suggesting that ecological exposures, rather than shared evolutionary history, drive viral community patterns within hosts. Together, these findings highlight the interconnected roles of host social and environmental exposure in shaping viral communities, with anthropogenic factors and macaque social organisation playing particularly significant roles. They also demonstrate the hierarchical or multiscale nature of viral community assembly, with different ecological processes operating at individual, group and site scales.

### 4.1 The Dominant Role of Anthropogenic Factors

Our findings confirm that human and livestock densities are the strongest predictors of PbV community composition and structure in rhesus macaques. These anthropogenic factors outweighed the effects of host characteristics and virus–virus associations, shaping viral diversity at both the individual and group levels. However, different viral taxa seemed to respond differently to human and livestock pressures. Specifically, both HDI and LDI had both significantly positive and significantly negative effects on viral OTUs. That is, while some OTUs correlated positively with higher human or livestock densities possibly indicating transmission from humans, livestock or their associated waste, other OTUs were significantly less common in areas with higher human or livestock densities, potentially indicating associations with natural environments where human activity is lower. In such settings, macaques may encounter OTUs associated with wildlife species or environmental reservoirs not typically found in densely human‐impacted environments. Testing the hypothesis that HDI and LDI drive distinct patterns of OTU occurrence through comparative analyses of macaque PbVs and sympatric human, livestock and wildlife PbVs would provide valuable insights into these dynamics (Engel and Jones‐Engel 2011; Wolfe et al. 1998, 2007). As such, our results here show that these anthropogenic factors positively impacted the occurrence of some OTUs while negatively impacting the occurrence of others, underscoring the complexity of viral community dynamics in human‐modified landscapes.

### 4.2 The Role of Host Social Organisation

Although anthropogenic factors were the strongest predictors of PbV community composition and structure, host social organisation still played a measurable role at the group and site (population) levels. Macaques within the same group and at more closely located sites had more similar PbV communities than those from different groups and more distantly located sites. Although this suggests that the organisation of macaques into groups does influence PbV communities, the underlying drivers remain unclear. One possibility is that PbV similarity within groups is driven by direct social interactions, such as grooming or aggressive contact (Albery et al. 2020). Alternatively, shared space use, rather than direct interactions, may be the primary driver, with macaques in the same group encountering similar environmental reservoirs or anthropogenic influences (Albery et al. 2020; Bradley and Altizer 2007). The latter explanation is supported by our finding that anthropogenic factors had a stronger impact on PbV community composition than macaques' group or site membership. Future research disentangling the relative contributions of wildlife social interactions vs. shared space use in shaping viral community ecology would thus be valuable (Albery et al. 2020).

### 4.3 Virus–Virus Associations in PbV Communities

As expected, virus–virus associations were strongest at the individual level, but their effect diminished at broader scales, such as among macaque groups within sites. This scale‐dependent dynamic highlights the localised nature of virus–virus interactions, which become less impactful as host social organisation and environmental exposures dominate. The observed co‐occurrence and exclusion patterns within hosts may reflect direct viral interactions, such as mutual facilitation (positive associations) or competitive exclusion (negative associations). Alternatively, these patterns could result from the bacterial host hypothesis: if PbVs primarily infect bacteria, which the evidence certainly supports (Krishnamurthy and Wang 2018; Wang 2022; Gan and Wang 2023), then co‐occurrence may reflect overlapping bacterial host ranges rather than direct virus–virus interactions. Site‐specific differences in bacterial community composition could further explain the diminishing influence of virus–virus associations at broader scales. Testing these hypotheses by integrating bacterial host data will be critical for understanding the ecological networks underlying these patterns.

### 4.4 Weak Phylogenetic Signals and Niche Conservatism

The lack of a strong phylogenetic signal in PbV communities suggests that ecological and environmental factors, rather than shared evolutionary history, play a dominant role in shaping viral co‐occurrence patterns. Although we observed significant virus–virus associations, these relationships were not driven by phylogenetic relatedness. Indeed, closely related OTUs did not co‐occur more frequently than distantly related ones in the same macaque, indicating that phylogenetic clustering was weak or absent. This pattern aligns with findings from studies on bats and rodents, which have similarly reported limited evidence for niche conservatism in viral communities (Luis et al. 2013; Wille 2020). One possible explanation is that functional traits, rather than phylogeny, drive viral community structure. Future work integrating functional virology approaches—such as examining replication efficiency or host entry mechanisms—could provide further insight into how PbVs assemble into communities.

Importantly, the weak role of phylogeny suggests that viral distributions may be more sensitive to external ecological factors than to intrinsic evolutionary constraints. This has important implications for predicting how viral communities will respond to ongoing environmental change and anthropogenic pressures. If viral distributions and interactions are primarily shaped by host exposure, land use changes or human–wildlife interactions, then shifts in these external conditions could rapidly reshape viral communities in unpredictable ways. However, while our findings indicate that phylogenetic constraints do not strongly shape PbV communities, further studies are needed to disentangle the relative contributions of ecological selection, functional constraints and stochastic processes in structuring viral diversity. Understanding how these forces interact will be essential for forecasting viral emergence patterns, particularly in human‐impacted environments.

### 4.5 Study Limitations

Our study had several important limitations that should be addressed in future research. First, increasing evidence suggests that PbVs are likely viruses of bacteria (Krishnamurthy and Wang 2018; Gan and Wang 2023; Ghosh and Malik 2021; Wang 2022). However, we did not collect bacterial community data, limiting our ability to evaluate the role of bacterial hosts in structuring PbV communities. This is particularly relevant given our findings that virus–virus associations may be influenced by site‐specific bacterial community composition. Future studies should integrate bacterial host data to test whether site‐specific differences in bacterial species distribution contribute to the observed patterns of viral community composition. Second, while our biological sampling spanned multiple macaque populations, we lacked fine‐grained behavioural data for specific groups. Behavioural data, such as macaque–macaque grooming frequencies, contact and noncontact aggression and space‐use overlap, would be essential to more convincingly disentangle independent viral acquisition from shared environmental reservoirs from viral transmission through direct social interactions with humans, livestock or other macaques (Albery et al. 2020; Bradley and Altizer 2007). These data would also allow for a more precise understanding of how social organisation interacts with environmental factors to shape viral community dynamics. Third, our data set was limited in the number of samples with associated demographic and (especially) cortisol data. This restricted the effective sample size of the JSDMs, as well as our ability to determine the impact of seasonal differences (dry versus wet) in sampling on viral communities since these shortlisted samples were all from the dry season. Although the JSDM analyses were constrained to 264 macaques, this subset was evenly distributed across groups and sites. Moreover, network analyses of PbV community structure included all sampled macaques and OTUs, ensuring a comprehensive assessment of viral community dynamics and the effects of social organisation. Nevertheless, expanding demographic and stress‐related datasets would allow for more robust modelling of the interactions between host factors and viral communities. Finally, while JSDMs proved useful for understanding the drivers of viral community composition, they are inherently static and unable to differentiate between simultaneous (e.g., multiple viral OTUs acquired from the same source) versus sequential (e.g., one OTU facilitating or inhibiting the acquisition of another) microbial acquisition (Brian and Aldridge 2021; Fountain‐Jones et al. 2019). This limitation is particularly relevant given our findings on the scale‐dependent dynamics of virus–virus associations, which likely vary over time. Longitudinal studies tracking viral communities in individual macaques over time would be critical for exploring temporal variation in viral communities in response to seasonal or environmental shifts. Despite these limitations, our study provides a robust framework for studying viral community dynamics in wildlife. Addressing these gaps—including the integration of bacterial host data, fine‐grained behavioural analyses and temporal tracking of viral communities—will help further advance our understanding of viral ecology and its response to ongoing environmental changes.

## Author Contributions

**Krishna N. Balasubramaniam:** conceptualization (equal), formal analysis (equal), investigation (equal), methodology (equal), validation (equal), visualization (equal), writing – original draft (equal), writing – review and editing (equal). **Isamara Navarrete‐Macias:** conceptualization (equal), data curation (equal), formal analysis (equal), investigation (equal), methodology (equal), validation (equal), visualization (equal), writing – review and editing (equal). **Shariful Islam:** data curation (equal), methodology (equal), resources (equal), writing – review and editing (equal). **Heather L. Wells:** formal analysis (equal), methodology (equal), writing – review and editing (equal). **Christopher Tubbs:** data curation (equal), methodology (equal), writing – review and editing (equal). **Nistara Randhawa:** software (equal), visualization (equal), writing – review and editing (equal). **Melinda K. Rostal:** data curation (equal), methodology (equal), writing – review and editing (equal). **Karin E. Darpel:** supervision (equal), writing – review and editing (equal). **Daniel Horton:** supervision (equal), writing – review and editing (equal). **Jonathan H. Epstein:** investigation (equal), project administration (equal), writing – review and editing (equal). **Ariful Islam:** investigation (equal), project administration (equal), writing – review and editing (equal). **Simon J. Anthony:** conceptualization (equal), data curation (equal), funding acquisition (equal), investigation (equal), methodology (equal), project administration (equal), resources (equal), software (equal), supervision (equal), validation (equal), writing – review and editing (equal).

## Conflicts of Interest

The authors declare no conflicts of interest.

## Supporting information


Appendix S1.

## Data Availability

The data analysed for this manuscript, along with the R code, has been made available at: https://doi.org/10.6084/m9.figshare.22492897.v3.
