# Supplementary material for: Anthropogenic Factors and Social Organisation Drive Picobirnavirus Communities in Wild Rhesus Macaques
Source: Ecol Evol. 2025 Jul 7;15(7):e71727. doi: 10.1002/ece3.71727 (PMC12230366; doi:10.1002/ece3.71727)
Supplement: Supplementary file 1 — Appendix S1. [file ECE3-15-e71727-s001.docx]

**Supporting Figures and Tables:**

**Supporting Table 1: Information about the macaque groups sampled, the study sites, the number of individual animals sampled at the group-level**

| **Group ID** | **Site ID** | **Site Coordinates**  **(^0^N) (^0^E)** | | **Sampling Seasons** | **Human Density** | **Livestock Density** | **Animals Sampled**  **(Group Size proxy)** |
| --- | --- | --- | --- | --- | --- | --- | --- |
| 1 | BBG | 24.2 | 90.5 | Wet; dry | Medium | High | 91 |
| 2 | BBG | 24.2 | 90.5 | Wet; dry | Medium | High | 49 |
| 3 | BBG | 24.2 | 90.5 | Wet; dry | Medium | High | 11 |
| 4 | BN | 23.6 | 90.5 | Wet; dry | High | Medium | 171 |
| 5 | BNPG | 24.0 | 90.4 | Wet; dry | Medium | Low | 110 |
| 6 | BNPG | 24.0 | 90.4 | Wet; dry | Medium | Low | 79 |
| 7 | BSMSPG | 24.2 | 90.4 | Wet; dry | Medium | Low | 69 |
| 8 | BSMSPG | 24.2 | 90.4 | Wet; dry | Medium | Low | 81 |
| 9 | CM | 23.2 | 90.2 | Wet; dry | Medium | High | 174 |
| 10 | CM | 23.2 | 90.2 | Wet; dry | Medium | High | 53 |
| 11 | CM | 23.2 | 90.2 | Wet; dry | Medium | High | 50 |
| 12 | CMS | 24.9 | 91.9 | Wet; dry | Medium | Medium | 113 |
| 13 | DD | 23.9 | 90.2 | Wet; dry | High | Medium | 119 |
| 14 | DD | 23.9 | 90.2 | Wet; dry | High | Medium | 72 |
| 15 | KR | 22.5 | 92.2 | Wet; dry | Low | Low | 69 |
| 16 | KR | 22.5 | 92.2 | Wet; dry | Low | Low | 28 |
| 17 | KS | 23.2 | 90.4 | Dry | Medium | Medium | 129 |
| 18 | KS | 23.2 | 90.4 | Dry | Medium | Medium | 32 |
| 19 | KSH | 23.2 | 90.4 | Wet; dry | Medium | Medium | 21 |
| 20 | MES | 24.9 | 91.9 | Wet; dry | Low | Low | 62 |
| 21 | ODD | 23.7 | 90.4 | Wet; dry | High | Low | 69 |
| 22 | ODD | 23.7 | 90.4 | Wet; dry | High | Low | 74 |
| 23 | PBC | 23.2 | 90.6 | Wet; dry | High | Medium | 62 |
| 24 | PBC | 23.2 | 90.6 | Wet; dry | High | Medium | 48 |
| 25 | PBC | 23.2 | 90.6 | Wet; dry | High | Medium | 26 |
| 26 | PKG | 24.1 | 90.6 | Dry | Medium | High | 73 |
| 27 | RBMR | 22.6 | 92.2 | Dry | Low | Medium | 83 |
| 28 | RN | 24.2 | 90.7 | Wet; dry | High | High | 64 |
| 29 | RN | 24.2 | 90.7 | Wet; dry | High | High | 29 |
| 30 | RN | 24.2 | 90.7 | Wet; dry | High | High | 52 |
| 31 | SJMS | 24.9 | 91.9 | Wet; dry | Low | Low | 77 |
| 32 | SPCB | 21.7 | 92.1 | Wet; dry | Low | Low | 31 |
| 33 | SPCB | 21.7 | 92.1 | Wet; dry | Low | Low | 91 |
| 34 | SPCB | 21.7 | 92.1 | Wet; dry | Low | Low | 39 |
| 35 | WB | 22.8 | 90.2 | Wet; dry | Medium | High | 62 |
| 36 | WB | 22.8 | 90.2 | Wet; dry | Medium | High | 63 |
| 37 | WB | 22.8 | 90.2 | Wet; dry | Medium | High | 37 |
| 38 | WB | 22.8 | 90.2 | Wet; dry | Medium | High | 21 |

Expansions of abbreviations used to indicate the various study sites:

BBG: Bormi Bazar (Gazipur); BN: Bandar (Narayangonj); BNPG: Bhawal National Park (Gazipur); BSMSPG: Bangabandhu Sheikh Mujib Safari Park (Gazipur); CM: Charmuguria (Madaripur); CMS: Chasnepeer Mazar (Sylhet); DD: Dhamrai (Dhaka); KR: Kaptai (Rangamati); KS: Kartikpur (Shariatpur); KSH: Kotapara (Shariatpur); MES: Malnichora Ecopark (Sylhet); ODD: Old Dhaka (Dhaka); PBC: Puran Bazaar (Chandpur); PKG: Panbarat-Kapasia (Gazipur); RBMR: Rajbon Buddha Mandir (Rangamati); RN: Rampur (Narsingdi); SJMS: Sayed Jahan Mazar (Syleht); SPCB: Safari Park (Cox Bazaar); WB: Wazirpur (Barisal).

**Supporting Table 2: Information about the 2664 individual macaques included in the study – their group ID, site ID, and (where collected) data on their age, sex, and faecal cortisol concentrations. ‘NA’ indicates that data is Not Available**

| **Animal ID** | **Site ID** | **Group ID** | **Human Density** | **Livestock Density** | **Age** | **Sex** | **Cortisol concentration**  **(ng/ml faeces)** |
| --- | --- | --- | --- | --- | --- | --- | --- |
| PRB02613 | BBG | BBGG1 | Medium | High | Adult | Female | 25.6 |
| PRB02615 | BBG | BBGG1 | Medium | High | NA | NA | 15 |
| PRB02617 | BBG | BBGG1 | Medium | High | NA | NA | 5 |
| PRB02619 | BBG | BBGG1 | Medium | High | NA | NA | 15.1 |
| PRB02621 | BBG | BBGG1 | Medium | High | Adult | Female | 8.9 |
| PRB02623 | BBG | BBGG1 | Medium | High | Adult | Female | 11.5 |
| PRB02625 | BBG | BBGG1 | Medium | High | NA | NA | NA |
| PRB02627 | BBG | BBGG1 | Medium | High | NA | NA | NA |
| PRB02629 | BBG | BBGG1 | Medium | High | NA | NA | 11.3 |
| PRB02631 | BBG | BBGG1 | Medium | High | NA | NA | 5.3 |
| PRB02633 | BBG | BBGG1 | Medium | High | NA | NA | 9.1 |
| PRB02635 | BBG | BBGG1 | Medium | High | NA | NA | 11.9 |
| PRB02637 | BBG | BBGG1 | Medium | High | NA | NA | 24.8 |
| PRB02639 | BBG | BBGG1 | Medium | High | NA | NA | 18.5 |
| PRB02641 | BBG | BBGG1 | Medium | High | NA | NA | 11 |
| PRB02643 | BBG | BBGG1 | Medium | High | NA | NA | 4.7 |
| PRB02645 | BBG | BBGG1 | Medium | High | NA | NA | 6.5 |
| PRB02647 | BBG | BBGG1 | Medium | High | Adult | NA | 7.4 |
| PRB02649 | BBG | BBGG1 | Medium | High | NA | NA | 7.7 |
| PRB06131 | BBG | BBGG1 | Medium | High | NA | NA | NA |
| PRB06133 | BBG | BBGG1 | Medium | High | NA | NA | NA |
| PRB06135 | BBG | BBGG1 | Medium | High | Young | Female | NA |
| PRB06137 | BBG | BBGG1 | Medium | High | NA | NA | NA |
| PRB06139 | BBG | BBGG1 | Medium | High | NA | NA | NA |
| PRB06141 | BBG | BBGG1 | Medium | High | NA | NA | NA |
| PRB06143 | BBG | BBGG1 | Medium | High | NA | NA | NA |
| PRB06145 | BBG | BBGG1 | Medium | High | NA | NA | NA |
| PRB06147 | BBG | BBGG1 | Medium | High | NA | NA | NA |
| PRB06149 | BBG | BBGG1 | Medium | High | NA | NA | NA |
| PRB06151 | BBG | BBGG1 | Medium | High | NA | NA | NA |
| PRB06153 | BBG | BBGG1 | Medium | High | NA | NA | NA |
| PRB06155 | BBG | BBGG1 | Medium | High | NA | NA | NA |
| PRB06157 | BBG | BBGG1 | Medium | High | NA | NA | NA |
| PRB06159 | BBG | BBGG1 | Medium | High | NA | NA | NA |
| PRB06161 | BBG | BBGG1 | Medium | High | NA | NA | NA |
| PRB06163 | BBG | BBGG1 | Medium | High | NA | NA | NA |
| PRB06165 | BBG | BBGG1 | Medium | High | NA | NA | NA |
| PRB06167 | BBG | BBGG1 | Medium | High | NA | NA | NA |
| PRB06169 | BBG | BBGG1 | Medium | High | Adult | Male | NA |
| PRB06171 | BBG | BBGG1 | Medium | High | NA | NA | NA |
| PRB06173 | BBG | BBGG1 | Medium | High | NA | NA | NA |
| PRB06175 | BBG | BBGG1 | Medium | High | NA | NA | NA |
| PRB06177 | BBG | BBGG1 | Medium | High | NA | NA | NA |
| PRB06179 | BBG | BBGG1 | Medium | High | NA | NA | NA |
| PRB06181 | BBG | BBGG1 | Medium | High | NA | NA | NA |
| PRB06183 | BBG | BBGG1 | Medium | High | NA | NA | NA |
| PRB06185 | BBG | BBGG1 | Medium | High | NA | NA | NA |
| PRB06187 | BBG | BBGG1 | Medium | High | NA | NA | NA |
| PRB06189 | BBG | BBGG1 | Medium | High | NA | NA | NA |
| PRB06191 | BBG | BBGG1 | Medium | High | NA | NA | NA |
| PRB06193 | BBG | BBGG1 | Medium | High | NA | NA | NA |
| PRB06195 | BBG | BBGG1 | Medium | High | NA | NA | NA |
| PRB06197 | BBG | BBGG1 | Medium | High | NA | NA | NA |
| PRB06199 | BBG | BBGG1 | Medium | High | NA | NA | NA |
| PRB06201 | BBG | BBGG1 | Medium | High | NA | NA | NA |
| PRB06203 | BBG | BBGG1 | Medium | High | NA | NA | NA |
| PRB06205 | BBG | BBGG1 | Medium | High | NA | NA | NA |
| PRB06207 | BBG | BBGG1 | Medium | High | NA | NA | NA |
| PRB06209 | BBG | BBGG1 | Medium | High | NA | NA | NA |
| PRB06211 | BBG | BBGG1 | Medium | High | NA | NA | NA |
| PRB06213 | BBG | BBGG1 | Medium | High | NA | NA | NA |
| PRB06215 | BBG | BBGG1 | Medium | High | NA | NA | NA |
| PRB06217 | BBG | BBGG1 | Medium | High | NA | NA | NA |
| PRB06219 | BBG | BBGG1 | Medium | High | NA | NA | NA |
| PRB06221 | BBG | BBGG1 | Medium | High | NA | NA | NA |
| PRB06223 | BBG | BBGG1 | Medium | High | NA | NA | NA |
| PRB06225 | BBG | BBGG1 | Medium | High | NA | NA | NA |
| PRB06227 | BBG | BBGG1 | Medium | High | NA | NA | NA |
| PRB06229 | BBG | BBGG1 | Medium | High | Adult | Male | NA |
| PRB06231 | BBG | BBGG1 | Medium | High | NA | NA | NA |
| PRB06233 | BBG | BBGG1 | Medium | High | NA | NA | NA |
| PRB06235 | BBG | BBGG1 | Medium | High | NA | NA | NA |
| PRB06237 | BBG | BBGG1 | Medium | High | NA | NA | NA |
| PRB06239 | BBG | BBGG1 | Medium | High | NA | NA | NA |
| PRB06241 | BBG | BBGG1 | Medium | High | NA | NA | NA |
| PRB06243 | BBG | BBGG1 | Medium | High | NA | NA | NA |
| PRB06245 | BBG | BBGG1 | Medium | High | NA | NA | NA |
| PRB06247 | BBG | BBGG1 | Medium | High | NA | NA | NA |
| PRB06249 | BBG | BBGG1 | Medium | High | NA | NA | NA |
| PRB06251 | BBG | BBGG1 | Medium | High | NA | NA | NA |
| PRB06253 | BBG | BBGG1 | Medium | High | NA | NA | NA |
| PRB06255 | BBG | BBGG1 | Medium | High | NA | NA | NA |
| PRB06257 | BBG | BBGG1 | Medium | High | NA | NA | NA |
| PRB06259 | BBG | BBGG1 | Medium | High | NA | NA | NA |
| PRB06261 | BBG | BBGG1 | Medium | High | NA | NA | NA |
| PRB06263 | BBG | BBGG1 | Medium | High | NA | NA | NA |
| PRB06265 | BBG | BBGG1 | Medium | High | NA | NA | NA |
| PRB06267 | BBG | BBGG1 | Medium | High | NA | NA | NA |
| PRB06269 | BBG | BBGG1 | Medium | High | NA | NA | NA |
| PRB06271 | BBG | BBGG1 | Medium | High | NA | NA | NA |
| PRB06273 | BBG | BBGG1 | Medium | High | NA | NA | NA |
| PRB03015 | BBG | BBGG2 | Medium | High | Adult | NA | 2.3 |
| PRB03017 | BBG | BBGG2 | Medium | High | Adult | NA | 1.6 |
| PRB03019 | BBG | BBGG2 | Medium | High | Adult | NA | 0.9 |
| PRB03021 | BBG | BBGG2 | Medium | High | Adult | NA | 17.2 |
| PRB03023 | BBG | BBGG2 | Medium | High | NA | NA | 0 |
| PRB03025 | BBG | BBGG2 | Medium | High | NA | NA | 3.7 |
| PRB03027 | BBG | BBGG2 | Medium | High | Adult | Male | 8.1 |
| PRB03029 | BBG | BBGG2 | Medium | High | NA | NA | 0.5 |
| PRB03031 | BBG | BBGG2 | Medium | High | NA | NA | 6 |
| PRB03033 | BBG | BBGG2 | Medium | High | Adult | Female | 7.8 |
| PRB03037 | BBG | BBGG2 | Medium | High | NA | NA | 7.4 |
| PRB03039 | BBG | BBGG2 | Medium | High | NA | NA | 3.2 |
| PRB03041 | BBG | BBGG2 | Medium | High | NA | NA | 1.3 |
| PRB03043 | BBG | BBGG2 | Medium | High | NA | NA | 2.6 |
| PRB03045 | BBG | BBGG2 | Medium | High | NA | NA | 4.4 |
| PRB03047 | BBG | BBGG2 | Medium | High | NA | NA | 0.9 |
| PRB03049 | BBG | BBGG2 | Medium | High | NA | NA | 0.3 |
| PRB03051 | BBG | BBGG2 | Medium | High | Adult | Female | 0.3 |
| PRB03055 | BBG | BBGG2 | Medium | High | Adult | NA | 0.4 |
| PRB03057 | BBG | BBGG2 | Medium | High | NA | NA | 0.3 |
| PRB03059 | BBG | BBGG2 | Medium | High | NA | NA | 0.4 |
| PRB03061 | BBG | BBGG2 | Medium | High | NA | NA | 0.5 |
| PRB03063 | BBG | BBGG2 | Medium | High | Adult | NA | 11.4 |
| PRB03065 | BBG | BBGG2 | Medium | High | NA | NA | 1.7 |
| PRB03067 | BBG | BBGG2 | Medium | High | NA | NA | 0.4 |
| PRB06035 | BBG | BBGG2 | Medium | High | NA | NA | NA |
| PRB06037 | BBG | BBGG2 | Medium | High | NA | NA | NA |
| PRB06039 | BBG | BBGG2 | Medium | High | NA | NA | NA |
| PRB06041 | BBG | BBGG2 | Medium | High | NA | NA | NA |
| PRB06043 | BBG | BBGG2 | Medium | High | NA | NA | NA |
| PRB06045 | BBG | BBGG2 | Medium | High | NA | NA | NA |
| PRB06047 | BBG | BBGG2 | Medium | High | NA | NA | NA |
| PRB06049 | BBG | BBGG2 | Medium | High | NA | NA | NA |
| PRB06051 | BBG | BBGG2 | Medium | High | NA | NA | NA |
| PRB06053 | BBG | BBGG2 | Medium | High | NA | NA | NA |
| PRB06055 | BBG | BBGG2 | Medium | High | NA | NA | NA |
| PRB06057 | BBG | BBGG2 | Medium | High | NA | NA | NA |
| PRB06059 | BBG | BBGG2 | Medium | High | NA | NA | NA |
| PRB06061 | BBG | BBGG2 | Medium | High | NA | NA | NA |
| PRB06063 | BBG | BBGG2 | Medium | High | NA | NA | NA |
| PRB06065 | BBG | BBGG2 | Medium | High | Adult | Male | NA |
| PRB06067 | BBG | BBGG2 | Medium | High | NA | NA | NA |
| PRB06069 | BBG | BBGG2 | Medium | High | NA | NA | NA |
| PRB06071 | BBG | BBGG2 | Medium | High | NA | NA | NA |
| PRB06073 | BBG | BBGG2 | Medium | High | NA | NA | NA |
| PRB06075 | BBG | BBGG2 | Medium | High | NA | NA | NA |
| PRB06077 | BBG | BBGG2 | Medium | High | NA | NA | NA |
| PRB06079 | BBG | BBGG2 | Medium | High | NA | NA | NA |
| PRB06081 | BBG | BBGG2 | Medium | High | NA | NA | NA |
| PRB03069 | BBG | BBGG3 | Medium | High | Adult | NA | 3.7 |
| PRB03071 | BBG | BBGG3 | Medium | High | NA | NA | 0.2 |
| PRB03073 | BBG | BBGG3 | Medium | High | Adult | NA | 3.3 |
| PRB03075 | BBG | BBGG3 | Medium | High | NA | NA | 1.5 |
| PRB03077 | BBG | BBGG3 | Medium | High | NA | NA | 4.2 |
| PRB03079 | BBG | BBGG3 | Medium | High | Adult | NA | 1.5 |
| PRB03081 | BBG | BBGG3 | Medium | High | NA | NA | 0.4 |
| PRB03083 | BBG | BBGG3 | Medium | High | Adult | Female | 2.3 |
| PRB03085 | BBG | BBGG3 | Medium | High | Adult | Female | 0.2 |
| PRB03091 | BBG | BBGG3 | Medium | High | Adult | Male | 3.6 |
| PRB03093 | BBG | BBGG3 | Medium | High | Adult | Female | 3.6 |
| PRB02871 | BN | BNG1 | High | Medium | Adult | Female | 0.4 |
| PRB02873 | BN | BNG1 | High | Medium | Adult | Female | 12.4 |
| PRB02875 | BN | BNG1 | High | Medium | Adult | Female | 35.2 |
| PRB02877 | BN | BNG1 | High | Medium | Adult | Male | 4 |
| PRB02881 | BN | BNG1 | High | Medium | Young | NA | 3.2 |
| PRB02883 | BN | BNG1 | High | Medium | Young | NA | 43.1 |
| PRB02885 | BN | BNG1 | High | Medium | Adult | NA | 19.1 |
| PRB02887 | BN | BNG1 | High | Medium | Adult | NA | 6.9 |
| PRB02889 | BN | BNG1 | High | Medium | Adult | Female | 14.6 |
| PRB02891 | BN | BNG1 | High | Medium | Adult | Female | 11.5 |
| PRB02893 | BN | BNG1 | High | Medium | Adult | Female | 20.1 |
| PRB02895 | BN | BNG1 | High | Medium | Adult | Female | 13.1 |
| PRB02897 | BN | BNG1 | High | Medium | Adult | Female | 17.5 |
| PRB02899 | BN | BNG1 | High | Medium | Young | Female | 7.9 |
| PRB02901 | BN | BNG1 | High | Medium | NA | NA | 17 |
| PRB02903 | BN | BNG1 | High | Medium | Young | Female | 20.1 |
| PRB02905 | BN | BNG1 | High | Medium | Adult | Female | 20 |
| PRB02907 | BN | BNG1 | High | Medium | Adult | Male | 9.9 |
| PRB02909 | BN | BNG1 | High | Medium | Young | NA | 13 |
| PRB02911 | BN | BNG1 | High | Medium | Adult | NA | 11.6 |
| PRB02913 | BN | BNG1 | High | Medium | Young | Male | 14.2 |
| PRB02915 | BN | BNG1 | High | Medium | Adult | Female | 18.7 |
| PRB02917 | BN | BNG1 | High | Medium | Adult | NA | 15.4 |
| PRB02919 | BN | BNG1 | High | Medium | Young | NA | 13.6 |
| PRB02921 | BN | BNG1 | High | Medium | Young | Female | 16.3 |
| PRB02923 | BN | BNG1 | High | Medium | Adult | Female | 8.7 |
| PRB02925 | BN | BNG1 | High | Medium | Young | Female | 14.9 |
| PRB02927 | BN | BNG1 | High | Medium | Young | Male | 35.6 |
| PRB02929 | BN | BNG1 | High | Medium | Adult | Female | 16.9 |
| PRB02931 | BN | BNG1 | High | Medium | Young | Female | 15.5 |
| PRB02933 | BN | BNG1 | High | Medium | Adult | NA | 9.8 |
| PRB02935 | BN | BNG1 | High | Medium | Young | Female | 18.6 |
| PRB02937 | BN | BNG1 | High | Medium | Adult | Female | 6.1 |
| PRB02939 | BN | BNG1 | High | Medium | Adult | Male | 27.6 |
| PRB02941 | BN | BNG1 | High | Medium | Adult | NA | 17.9 |
| PRB02943 | BN | BNG1 | High | Medium | Adult | NA | 12 |
| PRB02945 | BN | BNG1 | High | Medium | Young | Female | 3.9 |
| PRB02947 | BN | BNG1 | High | Medium | Young | Male | 15.6 |
| PRB02949 | BN | BNG1 | High | Medium | Young | Female | 17.5 |
| PRB02951 | BN | BNG1 | High | Medium | NA | NA | 0 |
| PRB02953 | BN | BNG1 | High | Medium | Young | Male | 13.1 |
| PRB02955 | BN | BNG1 | High | Medium | Young | Female | 24.7 |
| PRB02957 | BN | BNG1 | High | Medium | Adult | Male | 7.6 |
| PRB02959 | BN | BNG1 | High | Medium | Adult | Female | 4.1 |
| PRB02961 | BN | BNG1 | High | Medium | Young | Male | 4 |
| PRB02963 | BN | BNG1 | High | Medium | Adult | Female | 0 |
| PRB02965 | BN | BNG1 | High | Medium | NA | NA | 0 |
| PRB02967 | BN | BNG1 | High | Medium | Adult | Male | NA |
| PRB02969 | BN | BNG1 | High | Medium | Adult | Female | 9.1 |
| PRB02971 | BN | BNG1 | High | Medium | Adult | Female | 6.1 |
| PRB02973 | BN | BNG1 | High | Medium | Adult | Female | 8.7 |
| PRB02975 | BN | BNG1 | High | Medium | Adult | Female | 11.6 |
| PRB02977 | BN | BNG1 | High | Medium | Adult | Female | 7.3 |
| PRB02979 | BN | BNG1 | High | Medium | Adult | Female | 4.1 |
| PRB02981 | BN | BNG1 | High | Medium | Adult | Male | 6.1 |
| PRB02983 | BN | BNG1 | High | Medium | Young | NA | NA |
| PRB02985 | BN | BNG1 | High | Medium | Adult | Male | NA |
| PRB02987 | BN | BNG1 | High | Medium | Adult | Male | NA |
| PRB02989 | BN | BNG1 | High | Medium | Adult | NA | NA |
| PRB02991 | BN | BNG1 | High | Medium | Adult | Female | NA |
| PRB02993 | BN | BNG1 | High | Medium | Adult | Female | NA |
| PRB02995 | BN | BNG1 | High | Medium | Adult | Female | 4.7 |
| PRB05729 | BN | BNG1 | High | Medium | Young | Male | NA |
| PRB05731 | BN | BNG1 | High | Medium | Adult | Male | NA |
| PRB05733 | BN | BNG1 | High | Medium | Adult | Female | NA |
| PRB05735 | BN | BNG1 | High | Medium | Adult | Male | NA |
| PRB05737 | BN | BNG1 | High | Medium | Adult | Female | NA |
| PRB05739 | BN | BNG1 | High | Medium | Adult | Female | NA |
| PRB05741 | BN | BNG1 | High | Medium | Young | Female | NA |
| PRB05743 | BN | BNG1 | High | Medium | Adult | Female | NA |
| PRB05745 | BN | BNG1 | High | Medium | Young | Female | NA |
| PRB05747 | BN | BNG1 | High | Medium | Young | Female | NA |
| PRB05749 | BN | BNG1 | High | Medium | Young | Female | NA |
| PRB05751 | BN | BNG1 | High | Medium | Adult | Female | NA |
| PRB05753 | BN | BNG1 | High | Medium | Young | Female | NA |
| PRB05755 | BN | BNG1 | High | Medium | Young | Female | NA |
| PRB05757 | BN | BNG1 | High | Medium | Young | Male | NA |
| PRB05759 | BN | BNG1 | High | Medium | Adult | Female | NA |
| PRB05761 | BN | BNG1 | High | Medium | Adult | Female | NA |
| PRB05763 | BN | BNG1 | High | Medium | Adult | Male | NA |
| PRB05765 | BN | BNG1 | High | Medium | Adult | Male | NA |
| PRB05767 | BN | BNG1 | High | Medium | Adult | Female | NA |
| PRB05769 | BN | BNG1 | High | Medium | Adult | Female | NA |
| PRB05771 | BN | BNG1 | High | Medium | Young | Male | NA |
| PRB05773 | BN | BNG1 | High | Medium | Adult | Male | NA |
| PRB05775 | BN | BNG1 | High | Medium | Young | Male | NA |
| PRB05777 | BN | BNG1 | High | Medium | Adult | Male | NA |
| PRB05779 | BN | BNG1 | High | Medium | Adult | Female | NA |
| PRB05781 | BN | BNG1 | High | Medium | Adult | Female | NA |
| PRB05783 | BN | BNG1 | High | Medium | Young | Female | NA |
| PRB05785 | BN | BNG1 | High | Medium | Adult | Male | NA |
| PRB05787 | BN | BNG1 | High | Medium | Young | Male | NA |
| PRB05789 | BN | BNG1 | High | Medium | Adult | Female | NA |
| PRB05791 | BN | BNG1 | High | Medium | Adult | Male | NA |
| PRB05793 | BN | BNG1 | High | Medium | Adult | Female | NA |
| PRB05795 | BN | BNG1 | High | Medium | Adult | Female | NA |
| PRB05797 | BN | BNG1 | High | Medium | Adult | Female | NA |
| PRB05799 | BN | BNG1 | High | Medium | Young | Male | NA |
| PRB05801 | BN | BNG1 | High | Medium | Adult | Female | NA |
| PRB05803 | BN | BNG1 | High | Medium | Adult | Female | NA |
| PRB05805 | BN | BNG1 | High | Medium | Adult | Female | NA |
| PRB05807 | BN | BNG1 | High | Medium | Adult | Male | NA |
| PRB05809 | BN | BNG1 | High | Medium | Young | Male | NA |
| PRB05811 | BN | BNG1 | High | Medium | Adult | Female | NA |
| PRB05813 | BN | BNG1 | High | Medium | Young | Female | NA |
| PRB05815 | BN | BNG1 | High | Medium | Young | Male | NA |
| PRB05817 | BN | BNG1 | High | Medium | Adult | Male | NA |
| PRB05819 | BN | BNG1 | High | Medium | Young | Female | NA |
| PRB05821 | BN | BNG1 | High | Medium | Adult | Female | NA |
| PRB05823 | BN | BNG1 | High | Medium | Adult | Male | NA |
| PRB05825 | BN | BNG1 | High | Medium | Adult | Male | NA |
| PRB05827 | BN | BNG1 | High | Medium | Adult | Male | NA |
| PRB05829 | BN | BNG1 | High | Medium | Adult | Male | NA |
| PRB05831 | BN | BNG1 | High | Medium | Young | Female | NA |
| PRB05833 | BN | BNG1 | High | Medium | Young | Female | NA |
| PRB05835 | BN | BNG1 | High | Medium | Adult | Female | NA |
| PRB05837 | BN | BNG1 | High | Medium | Young | Male | NA |
| PRB05839 | BN | BNG1 | High | Medium | Adult | Male | NA |
| PRB05841 | BN | BNG1 | High | Medium | Adult | Female | NA |
| PRB05843 | BN | BNG1 | High | Medium | Adult | Male | NA |
| PRB05845 | BN | BNG1 | High | Medium | Adult | Female | NA |
| PRB05847 | BN | BNG1 | High | Medium | Young | Male | NA |
| PRB05849 | BN | BNG1 | High | Medium | Adult | Female | NA |
| PRB05851 | BN | BNG1 | High | Medium | Adult | Male | NA |
| PRB05853 | BN | BNG1 | High | Medium | Young | Female | NA |
| PRB05855 | BN | BNG1 | High | Medium | Adult | Male | NA |
| PRB05857 | BN | BNG1 | High | Medium | Adult | Male | NA |
| PRB05859 | BN | BNG1 | High | Medium | Adult | Female | NA |
| PRB05861 | BN | BNG1 | High | Medium | Adult | Female | NA |
| PRB05863 | BN | BNG1 | High | Medium | Adult | Female | NA |
| PRB05865 | BN | BNG1 | High | Medium | Adult | Male | NA |
| PRB05867 | BN | BNG1 | High | Medium | Adult | Female | NA |
| PRB05869 | BN | BNG1 | High | Medium | Adult | Male | NA |
| PRB05871 | BN | BNG1 | High | Medium | Adult | Female | NA |
| PRB05873 | BN | BNG1 | High | Medium | Young | Female | NA |
| PRB05875 | BN | BNG1 | High | Medium | Adult | Female | NA |
| PRB05877 | BN | BNG1 | High | Medium | Adult | Male | NA |
| PRB05879 | BN | BNG1 | High | Medium | Adult | Female | NA |
| PRB05881 | BN | BNG1 | High | Medium | Young | Female | NA |
| PRB05883 | BN | BNG1 | High | Medium | Young | Male | NA |
| PRB05885 | BN | BNG1 | High | Medium | Adult | Male | NA |
| PRB05887 | BN | BNG1 | High | Medium | Young | Female | NA |
| PRB05889 | BN | BNG1 | High | Medium | Young | Male | NA |
| PRB05891 | BN | BNG1 | High | Medium | Adult | Female | NA |
| PRB05893 | BN | BNG1 | High | Medium | Adult | Male | NA |
| PRB05895 | BN | BNG1 | High | Medium | Adult | Male | NA |
| PRB05897 | BN | BNG1 | High | Medium | Young | Male | NA |
| PRB05899 | BN | BNG1 | High | Medium | Adult | Female | NA |
| PRB05901 | BN | BNG1 | High | Medium | Young | Female | NA |
| PRB05903 | BN | BNG1 | High | Medium | Adult | Female | NA |
| PRB05905 | BN | BNG1 | High | Medium | Young | Male | NA |
| PRB05907 | BN | BNG1 | High | Medium | Young | Female | NA |
| PRB05909 | BN | BNG1 | High | Medium | Adult | Female | NA |
| PRB05911 | BN | BNG1 | High | Medium | Adult | Female | NA |
| PRB05913 | BN | BNG1 | High | Medium | Adult | Female | NA |
| PRB05915 | BN | BNG1 | High | Medium | Adult | Male | NA |
| PRB05917 | BN | BNG1 | High | Medium | Adult | Male | NA |
| PRB05919 | BN | BNG1 | High | Medium | Young | Male | NA |
| PRB05921 | BN | BNG1 | High | Medium | Adult | Female | NA |
| PRB05923 | BN | BNG1 | High | Medium | Adult | Female | NA |
| PRB05925 | BN | BNG1 | High | Medium | Adult | Male | NA |
| PRB05927 | BN | BNG1 | High | Medium | Adult | Male | NA |
| PRB05929 | BN | BNG1 | High | Medium | Adult | Female | NA |
| PRB05931 | BN | BNG1 | High | Medium | Adult | Male | NA |
| PRB05933 | BN | BNG1 | High | Medium | Young | Female | NA |
| PRB05935 | BN | BNG1 | High | Medium | Young | Female | NA |
| PRB05937 | BN | BNG1 | High | Medium | Young | Male | NA |
| PRB05939 | BN | BNG1 | High | Medium | Adult | Female | NA |
| PRB05941 | BN | BNG1 | High | Medium | Adult | Male | NA |
| PRB05943 | BN | BNG1 | High | Medium | Young | Female | NA |
| PRB05945 | BN | BNG1 | High | Medium | Young | Male | NA |
| PRB06575 | BNPG | BNPGC | Medium | Low | Adult | Female | NA |
| PRB06577 | BNPG | BNPGC | Medium | Low | NA | NA | NA |
| PRB06579 | BNPG | BNPGC | Medium | Low | NA | NA | NA |
| PRB06581 | BNPG | BNPGC | Medium | Low | NA | NA | NA |
| PRB06583 | BNPG | BNPGC | Medium | Low | NA | NA | NA |
| PRB06585 | BNPG | BNPGC | Medium | Low | NA | NA | NA |
| PRB06587 | BNPG | BNPGC | Medium | Low | NA | NA | NA |
| PRB06589 | BNPG | BNPGC | Medium | Low | NA | NA | NA |
| PRB06591 | BNPG | BNPGC | Medium | Low | NA | NA | NA |
| PRB06593 | BNPG | BNPGC | Medium | Low | NA | NA | NA |
| PRB06595 | BNPG | BNPGC | Medium | Low | NA | NA | NA |
| PRB06597 | BNPG | BNPGC | Medium | Low | NA | NA | NA |
| PRB06599 | BNPG | BNPGC | Medium | Low | NA | NA | NA |
| PRB06601 | BNPG | BNPGC | Medium | Low | NA | NA | NA |
| PRB06603 | BNPG | BNPGC | Medium | Low | NA | NA | NA |
| PRB06605 | BNPG | BNPGC | Medium | Low | NA | NA | NA |
| PRB06607 | BNPG | BNPGC | Medium | Low | NA | NA | NA |
| PRB06609 | BNPG | BNPGC | Medium | Low | NA | NA | NA |
| PRB06611 | BNPG | BNPGC | Medium | Low | NA | NA | NA |
| PRB06613 | BNPG | BNPGC | Medium | Low | NA | NA | NA |
| PRB06615 | BNPG | BNPGC | Medium | Low | NA | NA | NA |
| PRB06617 | BNPG | BNPGC | Medium | Low | NA | NA | NA |
| PRB06619 | BNPG | BNPGC | Medium | Low | NA | NA | NA |
| PRB06621 | BNPG | BNPGC | Medium | Low | NA | NA | NA |
| PRB06623 | BNPG | BNPGC | Medium | Low | NA | NA | NA |
| PRB06625 | BNPG | BNPGC | Medium | Low | NA | NA | NA |
| PRB06627 | BNPG | BNPGC | Medium | Low | NA | NA | NA |
| PRB06629 | BNPG | BNPGC | Medium | Low | NA | NA | NA |
| PRB06631 | BNPG | BNPGC | Medium | Low | NA | NA | NA |
| PRB06633 | BNPG | BNPGC | Medium | Low | NA | NA | NA |
| PRB06635 | BNPG | BNPGC | Medium | Low | NA | NA | NA |
| PRB06637 | BNPG | BNPGC | Medium | Low | Adult | Male | NA |
| PRB06639 | BNPG | BNPGC | Medium | Low | NA | NA | NA |
| PRB06641 | BNPG | BNPGC | Medium | Low | NA | NA | NA |
| PRB06643 | BNPG | BNPGC | Medium | Low | NA | NA | NA |
| PRB06645 | BNPG | BNPGC | Medium | Low | NA | NA | NA |
| PRB06647 | BNPG | BNPGC | Medium | Low | NA | NA | NA |
| PRB06649 | BNPG | BNPGC | Medium | Low | NA | NA | NA |
| PRB06651 | BNPG | BNPGC | Medium | Low | NA | NA | NA |
| PRB06653 | BNPG | BNPGC | Medium | Low | NA | NA | NA |
| PRB06655 | BNPG | BNPGC | Medium | Low | NA | NA | NA |
| PRB06657 | BNPG | BNPGC | Medium | Low | NA | NA | NA |
| PRB06659 | BNPG | BNPGC | Medium | Low | NA | NA | NA |
| PRB06661 | BNPG | BNPGC | Medium | Low | NA | NA | NA |
| PRB06663 | BNPG | BNPGC | Medium | Low | NA | NA | NA |
| PRB06665 | BNPG | BNPGC | Medium | Low | NA | NA | NA |
| PRB06667 | BNPG | BNPGC | Medium | Low | NA | NA | NA |
| PRB06669 | BNPG | BNPGC | Medium | Low | NA | NA | NA |
| PRB06671 | BNPG | BNPGC | Medium | Low | NA | NA | NA |
| PRB06673 | BNPG | BNPGC | Medium | Low | NA | NA | NA |
| PRB06675 | BNPG | BNPGC | Medium | Low | NA | NA | NA |
| PRB06677 | BNPG | BNPGC | Medium | Low | NA | NA | NA |
| PRB06679 | BNPG | BNPGC | Medium | Low | NA | NA | NA |
| PRB06681 | BNPG | BNPGC | Medium | Low | NA | NA | NA |
| PRB06683 | BNPG | BNPGC | Medium | Low | NA | NA | NA |
| PRB06685 | BNPG | BNPGC | Medium | Low | NA | NA | NA |
| PRB06687 | BNPG | BNPGC | Medium | Low | NA | NA | NA |
| PRB06689 | BNPG | BNPGC | Medium | Low | NA | NA | NA |
| PRB06691 | BNPG | BNPGC | Medium | Low | NA | NA | NA |
| PRB06693 | BNPG | BNPGC | Medium | Low | NA | NA | NA |
| PRB02753 | BNPG | BNPGG1 | Medium | Low | Adult | Male | 17.2 |
| PRB02755 | BNPG | BNPGG1 | Medium | Low | Adult | Male | 34.9 |
| PRB02757 | BNPG | BNPGG1 | Medium | Low | Adult | Male | 14.4 |
| PRB03691 | BNPG | BNPGG1 | Medium | Low | Adult | Male | 8.4 |
| PRB03693 | BNPG | BNPGG1 | Medium | Low | Adult | NA | 3.4 |
| PRB03695 | BNPG | BNPGG1 | Medium | Low | Adult | Female | 1.8 |
| PRB03697 | BNPG | BNPGG1 | Medium | Low | Adult | NA | 0.6 |
| PRB03699 | BNPG | BNPGG1 | Medium | Low | Young | NA | 9.7 |
| PRB03701 | BNPG | BNPGG1 | Medium | Low | Adult | Male | 0.5 |
| PRB03703 | BNPG | BNPGG1 | Medium | Low | Adult | Female | 0 |
| PRB03705 | BNPG | BNPGG1 | Medium | Low | Adult | Male | 0.6 |
| PRB03707 | BNPG | BNPGG1 | Medium | Low | Adult | Female | NA |
| PRB03709 | BNPG | BNPGG1 | Medium | Low | Adult | Female | 6.7 |
| PRB03711 | BNPG | BNPGG1 | Medium | Low | Young | NA | 0 |
| PRB03713 | BNPG | BNPGG1 | Medium | Low | Adult | Male | 0 |
| PRB03715 | BNPG | BNPGG1 | Medium | Low | Adult | Female | 4.5 |
| PRB03717 | BNPG | BNPGG1 | Medium | Low | Adult | Female | 0.2 |
| PRB03719 | BNPG | BNPGG1 | Medium | Low | Adult | Female | 5.4 |
| PRB03721 | BNPG | BNPGG1 | Medium | Low | Adult | Female | 0 |
| PRB03723 | BNPG | BNPGG1 | Medium | Low | Adult | Female | 0.3 |
| PRB03725 | BNPG | BNPGG1 | Medium | Low | Young | NA | 0.3 |
| PRB03727 | BNPG | BNPGG1 | Medium | Low | Adult | Female | 0.2 |
| PRB03729 | BNPG | BNPGG1 | Medium | Low | Young | NA | 1.3 |
| PRB03731 | BNPG | BNPGG1 | Medium | Low | Adult | Female | 0 |
| PRB03733 | BNPG | BNPGG1 | Medium | Low | Adult | Male | 3.5 |
| PRB03735 | BNPG | BNPGG1 | Medium | Low | Adult | Male | 0.8 |
| PRB03737 | BNPG | BNPGG1 | Medium | Low | Adult | Female | 0.6 |
| PRB03739 | BNPG | BNPGG1 | Medium | Low | Adult | Female | 5.6 |
| PRB03741 | BNPG | BNPGG1 | Medium | Low | Adult | Female | 2.8 |
| PRB03743 | BNPG | BNPGG1 | Medium | Low | Young | NA | 0 |
| PRB03745 | BNPG | BNPGG1 | Medium | Low | Adult | Female | 1.5 |
| PRB03747 | BNPG | BNPGG1 | Medium | Low | Adult | Female | 1.1 |
| PRB03749 | BNPG | BNPGG1 | Medium | Low | Adult | Male | 10.4 |
| PRB03751 | BNPG | BNPGG1 | Medium | Low | Young | NA | 4.7 |
| PRB07303 | BNPG | BNPGG1 | Medium | Low | NA | NA | NA |
| PRB07305 | BNPG | BNPGG1 | Medium | Low | NA | NA | NA |
| PRB07307 | BNPG | BNPGG1 | Medium | Low | NA | NA | NA |
| PRB07309 | BNPG | BNPGG1 | Medium | Low | NA | NA | NA |
| PRB07311 | BNPG | BNPGG1 | Medium | Low | NA | NA | NA |
| PRB07313 | BNPG | BNPGG1 | Medium | Low | NA | NA | NA |
| PRB07315 | BNPG | BNPGG1 | Medium | Low | NA | NA | NA |
| PRB07317 | BNPG | BNPGG1 | Medium | Low | NA | NA | NA |
| PRB07319 | BNPG | BNPGG1 | Medium | Low | NA | NA | NA |
| PRB07321 | BNPG | BNPGG1 | Medium | Low | NA | NA | NA |
| PRB07323 | BNPG | BNPGG1 | Medium | Low | NA | NA | NA |
| PRB07325 | BNPG | BNPGG1 | Medium | Low | NA | NA | NA |
| PRB07327 | BNPG | BNPGG1 | Medium | Low | NA | NA | NA |
| PRB07329 | BNPG | BNPGG1 | Medium | Low | NA | NA | NA |
| PRB07331 | BNPG | BNPGG1 | Medium | Low | NA | NA | NA |
| PRB07333 | BNPG | BNPGG1 | Medium | Low | Adult | Male | NA |
| PRB07335 | BNPG | BNPGG1 | Medium | Low | NA | NA | NA |
| PRB07337 | BNPG | BNPGG1 | Medium | Low | NA | NA | NA |
| PRB07339 | BNPG | BNPGG1 | Medium | Low | NA | NA | NA |
| PRB07341 | BNPG | BNPGG1 | Medium | Low | NA | NA | NA |
| PRB07343 | BNPG | BNPGG1 | Medium | Low | NA | NA | NA |
| PRB07345 | BNPG | BNPGG1 | Medium | Low | NA | NA | NA |
| PRB07347 | BNPG | BNPGG1 | Medium | Low | NA | NA | NA |
| PRB07349 | BNPG | BNPGG1 | Medium | Low | NA | NA | NA |
| PRB07351 | BNPG | BNPGG1 | Medium | Low | NA | NA | NA |
| PRB07353 | BNPG | BNPGG1 | Medium | Low | NA | NA | NA |
| PRB07355 | BNPG | BNPGG1 | Medium | Low | NA | NA | NA |
| PRB07357 | BNPG | BNPGG1 | Medium | Low | Adult | Male | NA |
| PRB07359 | BNPG | BNPGG1 | Medium | Low | NA | NA | NA |
| PRB07361 | BNPG | BNPGG1 | Medium | Low | Adult | Male | NA |
| PRB07363 | BNPG | BNPGG1 | Medium | Low | NA | NA | NA |
| PRB07365 | BNPG | BNPGG1 | Medium | Low | NA | NA | NA |
| PRB07367 | BNPG | BNPGG1 | Medium | Low | NA | NA | NA |
| PRB07369 | BNPG | BNPGG1 | Medium | Low | NA | NA | NA |
| PRB07371 | BNPG | BNPGG1 | Medium | Low | NA | NA | NA |
| PRB07373 | BNPG | BNPGG1 | Medium | Low | NA | NA | NA |
| PRB07375 | BNPG | BNPGG1 | Medium | Low | NA | NA | NA |
| PRB07377 | BNPG | BNPGG1 | Medium | Low | NA | NA | NA |
| PRB07379 | BNPG | BNPGG1 | Medium | Low | NA | NA | NA |
| PRB07381 | BNPG | BNPGG1 | Medium | Low | NA | NA | NA |
| PRB07383 | BNPG | BNPGG1 | Medium | Low | NA | NA | NA |
| PRB07385 | BNPG | BNPGG1 | Medium | Low | NA | NA | NA |
| PRB07387 | BNPG | BNPGG1 | Medium | Low | NA | NA | NA |
| PRB07389 | BNPG | BNPGG1 | Medium | Low | NA | NA | NA |
| PRB07391 | BNPG | BNPGG1 | Medium | Low | NA | NA | NA |
| PRB07393 | BNPG | BNPGG1 | Medium | Low | NA | NA | NA |
| PRB07395 | BNPG | BNPGG1 | Medium | Low | NA | NA | NA |
| PRB07397 | BNPG | BNPGG1 | Medium | Low | NA | NA | NA |
| PRB07399 | BNPG | BNPGG1 | Medium | Low | NA | NA | NA |
| PRB07401 | BNPG | BNPGG1 | Medium | Low | NA | NA | NA |
| PRB07403 | BNPG | BNPGG1 | Medium | Low | NA | NA | NA |
| PRB07405 | BNPG | BNPGG1 | Medium | Low | NA | NA | NA |
| PRB07407 | BNPG | BNPGG1 | Medium | Low | NA | NA | NA |
| PRB07409 | BNPG | BNPGG1 | Medium | Low | NA | NA | NA |
| PRB07411 | BNPG | BNPGG1 | Medium | Low | NA | NA | NA |
| PRB07413 | BNPG | BNPGG1 | Medium | Low | NA | NA | NA |
| PRB07415 | BNPG | BNPGG1 | Medium | Low | NA | NA | NA |
| PRB07417 | BNPG | BNPGG1 | Medium | Low | NA | NA | NA |
| PRB07419 | BNPG | BNPGG1 | Medium | Low | NA | NA | NA |
| PRB07421 | BNPG | BNPGG1 | Medium | Low | NA | NA | NA |
| PRB07423 | BNPG | BNPGG1 | Medium | Low | NA | NA | NA |
| PRB07425 | BNPG | BNPGG1 | Medium | Low | NA | NA | NA |
| PRB07427 | BNPG | BNPGG1 | Medium | Low | NA | NA | NA |
| PRB07429 | BNPG | BNPGG1 | Medium | Low | NA | NA | NA |
| PRB07431 | BNPG | BNPGG1 | Medium | Low | NA | NA | NA |
| PRB07433 | BNPG | BNPGG1 | Medium | Low | NA | NA | NA |
| PRB07435 | BNPG | BNPGG1 | Medium | Low | NA | NA | NA |
| PRB07437 | BNPG | BNPGG1 | Medium | Low | NA | NA | NA |
| PRB07439 | BNPG | BNPGG1 | Medium | Low | NA | NA | NA |
| PRB07441 | BNPG | BNPGG1 | Medium | Low | NA | NA | NA |
| PRB07443 | BNPG | BNPGG1 | Medium | Low | NA | NA | NA |
| PRB07445 | BNPG | BNPGG1 | Medium | Low | NA | NA | NA |
| PRB07447 | BNPG | BNPGG1 | Medium | Low | NA | NA | NA |
| PRB07449 | BNPG | BNPGG1 | Medium | Low | NA | NA | NA |
| PRB07451 | BNPG | BNPGG1 | Medium | Low | NA | NA | NA |
| PRB07453 | BNPG | BNPGG1 | Medium | Low | NA | NA | NA |
| PRB07455 | BNPG | BNPGG2 | Medium | Low | NA | NA | NA |
| PRB07457 | BNPG | BNPGG2 | Medium | Low | NA | NA | NA |
| PRB07459 | BNPG | BNPGG2 | Medium | Low | NA | NA | NA |
| PRB07461 | BNPG | BNPGG2 | Medium | Low | NA | NA | NA |
| PRB07463 | BNPG | BNPGG2 | Medium | Low | NA | NA | NA |
| PRB07465 | BNPG | BNPGG2 | Medium | Low | NA | NA | NA |
| PRB07467 | BNPG | BNPGG2 | Medium | Low | NA | NA | NA |
| PRB07469 | BNPG | BNPGG2 | Medium | Low | NA | NA | NA |
| PRB07471 | BNPG | BNPGG2 | Medium | Low | NA | NA | NA |
| PRB07473 | BNPG | BNPGG2 | Medium | Low | NA | NA | NA |
| PRB07475 | BNPG | BNPGG2 | Medium | Low | NA | NA | NA |
| PRB07477 | BNPG | BNPGG2 | Medium | Low | NA | NA | NA |
| PRB07479 | BNPG | BNPGG2 | Medium | Low | NA | NA | NA |
| PRB07481 | BNPG | BNPGG2 | Medium | Low | NA | NA | NA |
| PRB07483 | BNPG | BNPGG2 | Medium | Low | NA | NA | NA |
| PRB07485 | BNPG | BNPGG2 | Medium | Low | NA | NA | NA |
| PRB07487 | BNPG | BNPGG2 | Medium | Low | NA | NA | NA |
| PRB07489 | BNPG | BNPGG2 | Medium | Low | NA | NA | NA |
| PRB07491 | BNPG | BNPGG2 | Medium | Low | NA | NA | NA |
| PRB07493 | BNPG | BNPGG2 | Medium | Low | NA | NA | NA |
| PRB07495 | BNPG | BNPGG2 | Medium | Low | NA | NA | NA |
| PRB07497 | BNPG | BNPGG2 | Medium | Low | NA | NA | NA |
| PRB07499 | BNPG | BNPGG2 | Medium | Low | NA | NA | NA |
| PRB07501 | BNPG | BNPGG2 | Medium | Low | NA | NA | NA |
| PRB07503 | BNPG | BNPGG2 | Medium | Low | NA | NA | NA |
| PRB07505 | BNPG | BNPGG2 | Medium | Low | NA | NA | NA |
| PRB07507 | BNPG | BNPGG2 | Medium | Low | NA | NA | NA |
| PRB07509 | BNPG | BNPGG2 | Medium | Low | Adult | Male | NA |
| PRB07511 | BNPG | BNPGG2 | Medium | Low | NA | NA | NA |
| PRB07513 | BNPG | BNPGG2 | Medium | Low | NA | NA | NA |
| PRB07515 | BNPG | BNPGG2 | Medium | Low | NA | NA | NA |
| PRB07517 | BNPG | BNPGG2 | Medium | Low | NA | NA | NA |
| PRB07519 | BNPG | BNPGG2 | Medium | Low | NA | NA | NA |
| PRB07521 | BNPG | BNPGG2 | Medium | Low | NA | NA | NA |
| PRB07523 | BNPG | BNPGG2 | Medium | Low | NA | NA | NA |
| PRB07525 | BNPG | BNPGG2 | Medium | Low | NA | NA | NA |
| PRB07527 | BNPG | BNPGG2 | Medium | Low | NA | NA | NA |
| PRB07529 | BNPG | BNPGG2 | Medium | Low | NA | NA | NA |
| PRB07531 | BNPG | BNPGG2 | Medium | Low | NA | NA | NA |
| PRB07533 | BNPG | BNPGG2 | Medium | Low | NA | NA | NA |
| PRB07535 | BNPG | BNPGG2 | Medium | Low | NA | NA | NA |
| PRB07537 | BNPG | BNPGG2 | Medium | Low | NA | NA | NA |
| PRB07539 | BNPG | BNPGG2 | Medium | Low | NA | NA | NA |
| PRB07541 | BNPG | BNPGG2 | Medium | Low | NA | NA | NA |
| PRB07543 | BNPG | BNPGG2 | Medium | Low | NA | NA | NA |
| PRB07545 | BNPG | BNPGG2 | Medium | Low | NA | NA | NA |
| PRB07547 | BNPG | BNPGG2 | Medium | Low | NA | NA | NA |
| PRB07549 | BNPG | BNPGG2 | Medium | Low | NA | NA | NA |
| PRB07551 | BNPG | BNPGG2 | Medium | Low | NA | NA | NA |
| PRB07553 | BNPG | BNPGG2 | Medium | Low | NA | NA | NA |
| PRB07555 | BNPG | BNPGG2 | Medium | Low | NA | NA | NA |
| PRB07557 | BNPG | BNPGG2 | Medium | Low | NA | NA | NA |
| PRB07559 | BNPG | BNPGG2 | Medium | Low | NA | NA | NA |
| PRB07561 | BNPG | BNPGG2 | Medium | Low | NA | NA | NA |
| PRB07563 | BNPG | BNPGG2 | Medium | Low | NA | NA | NA |
| PRB07565 | BNPG | BNPGG2 | Medium | Low | NA | NA | NA |
| PRB07567 | BNPG | BNPGG2 | Medium | Low | NA | NA | NA |
| PRB07569 | BNPG | BNPGG2 | Medium | Low | NA | NA | NA |
| PRB07571 | BNPG | BNPGG2 | Medium | Low | NA | NA | NA |
| PRB07573 | BNPG | BNPGG2 | Medium | Low | NA | NA | NA |
| PRB07575 | BNPG | BNPGG2 | Medium | Low | NA | NA | NA |
| PRB07577 | BNPG | BNPGG2 | Medium | Low | NA | NA | NA |
| PRB07579 | BNPG | BNPGG2 | Medium | Low | NA | NA | NA |
| PRB07581 | BNPG | BNPGG2 | Medium | Low | NA | NA | NA |
| PRB07583 | BNPG | BNPGG2 | Medium | Low | NA | NA | NA |
| PRB07585 | BNPG | BNPGG2 | Medium | Low | NA | NA | NA |
| PRB07587 | BNPG | BNPGG2 | Medium | Low | NA | NA | NA |
| PRB07589 | BNPG | BNPGG2 | Medium | Low | NA | NA | NA |
| PRB07591 | BNPG | BNPGG2 | Medium | Low | NA | NA | NA |
| PRB07593 | BNPG | BNPGG2 | Medium | Low | NA | NA | NA |
| PRB07595 | BNPG | BNPGG2 | Medium | Low | NA | NA | NA |
| PRB07597 | BNPG | BNPGG2 | Medium | Low | NA | NA | NA |
| PRB07599 | BNPG | BNPGG2 | Medium | Low | NA | NA | NA |
| PRB07601 | BNPG | BNPGG2 | Medium | Low | NA | NA | NA |
| PRB07603 | BNPG | BNPGG2 | Medium | Low | NA | NA | NA |
| PRB07605 | BNPG | BNPGG2 | Medium | Low | NA | NA | NA |
| PRB07607 | BNPG | BNPGG2 | Medium | Low | NA | NA | NA |
| PRB07609 | BNPG | BNPGG2 | Medium | Low | NA | NA | NA |
| PRB07611 | BNPG | BNPGG2 | Medium | Low | NA | NA | NA |
| PRB03545 | BSMSPG | BSMSPGG1 | Medium | Low | Adult | Female | 0.2 |
| PRB03547 | BSMSPG | BSMSPGG1 | Medium | Low | Adult | Male | 0 |
| PRB03549 | BSMSPG | BSMSPGG1 | Medium | Low | Adult | Female | 0 |
| PRB03551 | BSMSPG | BSMSPGG1 | Medium | Low | Adult | NA | 0.7 |
| PRB03553 | BSMSPG | BSMSPGG1 | Medium | Low | Adult | Male | 0.4 |
| PRB03555 | BSMSPG | BSMSPGG1 | Medium | Low | Young | NA | 0 |
| PRB03557 | BSMSPG | BSMSPGG1 | Medium | Low | Adult | NA | 9.4 |
| PRB03559 | BSMSPG | BSMSPGG1 | Medium | Low | Adult | Female | 1.3 |
| PRB03561 | BSMSPG | BSMSPGG1 | Medium | Low | Adult | NA | 0.5 |
| PRB03563 | BSMSPG | BSMSPGG1 | Medium | Low | Adult | NA | 1.3 |
| PRB03565 | BSMSPG | BSMSPGG1 | Medium | Low | Adult | Male | NA |
| PRB03567 | BSMSPG | BSMSPGG1 | Medium | Low | Adult | NA | NA |
| PRB03569 | BSMSPG | BSMSPGG1 | Medium | Low | Adult | NA | 17 |
| PRB03571 | BSMSPG | BSMSPGG1 | Medium | Low | Young | NA | NA |
| PRB03573 | BSMSPG | BSMSPGG1 | Medium | Low | Young | NA | NA |
| PRB03575 | BSMSPG | BSMSPGG1 | Medium | Low | Young | Male | 1.5 |
| PRB03577 | BSMSPG | BSMSPGG1 | Medium | Low | Young | NA | NA |
| PRB03579 | BSMSPG | BSMSPGG1 | Medium | Low | Adult | Female | 1.3 |
| PRB03581 | BSMSPG | BSMSPGG1 | Medium | Low | Adult | NA | 1.6 |
| PRB03583 | BSMSPG | BSMSPGG1 | Medium | Low | Young | NA | NA |
| PRB03585 | BSMSPG | BSMSPGG1 | Medium | Low | Adult | NA | 0.1 |
| PRB03587 | BSMSPG | BSMSPGG1 | Medium | Low | Adult | NA | 2.9 |
| PRB03589 | BSMSPG | BSMSPGG1 | Medium | Low | Adult | NA | 2.2 |
| PRB03591 | BSMSPG | BSMSPGG1 | Medium | Low | Adult | Male | NA |
| PRB03593 | BSMSPG | BSMSPGG1 | Medium | Low | Adult | NA | 0 |
| PRB03595 | BSMSPG | BSMSPGG1 | Medium | Low | Young | NA | 0 |
| PRB03597 | BSMSPG | BSMSPGG1 | Medium | Low | Young | NA | 3.9 |
| PRB03599 | BSMSPG | BSMSPGG1 | Medium | Low | Young | NA | 1.9 |
| PRB03601 | BSMSPG | BSMSPGG1 | Medium | Low | Adult | NA | 4.9 |
| PRB03603 | BSMSPG | BSMSPGG1 | Medium | Low | Young | NA | 0 |
| PRB03605 | BSMSPG | BSMSPGG1 | Medium | Low | Adult | NA | 1.3 |
| PRB07077 | BSMSPG | BSMSPGG1 | Medium | Low | NA | NA | NA |
| PRB07079 | BSMSPG | BSMSPGG1 | Medium | Low | NA | NA | NA |
| PRB07081 | BSMSPG | BSMSPGG1 | Medium | Low | NA | NA | NA |
| PRB07083 | BSMSPG | BSMSPGG1 | Medium | Low | NA | NA | NA |
| PRB07085 | BSMSPG | BSMSPGG1 | Medium | Low | NA | NA | NA |
| PRB07087 | BSMSPG | BSMSPGG1 | Medium | Low | Adult | Male | NA |
| PRB07089 | BSMSPG | BSMSPGG1 | Medium | Low | NA | NA | NA |
| PRB07091 | BSMSPG | BSMSPGG1 | Medium | Low | NA | NA | NA |
| PRB07093 | BSMSPG | BSMSPGG1 | Medium | Low | NA | NA | NA |
| PRB07099 | BSMSPG | BSMSPGG1 | Medium | Low | NA | NA | NA |
| PRB07101 | BSMSPG | BSMSPGG1 | Medium | Low | NA | NA | NA |
| PRB07103 | BSMSPG | BSMSPGG1 | Medium | Low | NA | NA | NA |
| PRB07105 | BSMSPG | BSMSPGG1 | Medium | Low | NA | NA | NA |
| PRB07107 | BSMSPG | BSMSPGG1 | Medium | Low | NA | NA | NA |
| PRB07109 | BSMSPG | BSMSPGG1 | Medium | Low | NA | NA | NA |
| PRB07111 | BSMSPG | BSMSPGG1 | Medium | Low | NA | Male | NA |
| PRB07113 | BSMSPG | BSMSPGG1 | Medium | Low | Adult | Male | NA |
| PRB07115 | BSMSPG | BSMSPGG1 | Medium | Low | NA | Female | NA |
| PRB07117 | BSMSPG | BSMSPGG1 | Medium | Low | NA | NA | NA |
| PRB07119 | BSMSPG | BSMSPGG1 | Medium | Low | Adult | Female | NA |
| PRB07121 | BSMSPG | BSMSPGG1 | Medium | Low | NA | NA | NA |
| PRB07123 | BSMSPG | BSMSPGG1 | Medium | Low | NA | NA | NA |
| PRB07125 | BSMSPG | BSMSPGG1 | Medium | Low | NA | NA | NA |
| PRB07127 | BSMSPG | BSMSPGG1 | Medium | Low | NA | NA | NA |
| PRB07129 | BSMSPG | BSMSPGG1 | Medium | Low | NA | NA | NA |
| PRB07131 | BSMSPG | BSMSPGG1 | Medium | Low | NA | NA | NA |
| PRB07231 | BSMSPG | BSMSPGG1 | Medium | Low | Adult | Male | NA |
| PRB07233 | BSMSPG | BSMSPGG1 | Medium | Low | NA | NA | NA |
| PRB07235 | BSMSPG | BSMSPGG1 | Medium | Low | NA | NA | NA |
| PRB07237 | BSMSPG | BSMSPGG1 | Medium | Low | NA | NA | NA |
| PRB07239 | BSMSPG | BSMSPGG1 | Medium | Low | NA | NA | NA |
| PRB07241 | BSMSPG | BSMSPGG1 | Medium | Low | NA | NA | NA |
| PRB07243 | BSMSPG | BSMSPGG1 | Medium | Low | NA | NA | NA |
| PRB07245 | BSMSPG | BSMSPGG1 | Medium | Low | NA | NA | NA |
| PRB07247 | BSMSPG | BSMSPGG1 | Medium | Low | NA | NA | NA |
| PRB07249 | BSMSPG | BSMSPGG1 | Medium | Low | NA | NA | NA |
| PRB07251 | BSMSPG | BSMSPGG1 | Medium | Low | NA | NA | NA |
| PRB07253 | BSMSPG | BSMSPGG1 | Medium | Low | NA | NA | NA |
| PRB04085 | BSMSPG | BSMSPGG2 | Medium | Low | Adult | Male | 3.6 |
| PRB04087 | BSMSPG | BSMSPGG2 | Medium | Low | Adult | NA | 0 |
| PRB04089 | BSMSPG | BSMSPGG2 | Medium | Low | NA | NA | 5.4 |
| PRB04091 | BSMSPG | BSMSPGG2 | Medium | Low | NA | NA | 1.3 |
| PRB04093 | BSMSPG | BSMSPGG2 | Medium | Low | Young | NA | 0 |
| PRB04095 | BSMSPG | BSMSPGG2 | Medium | Low | Young | NA | 0 |
| PRB04097 | BSMSPG | BSMSPGG2 | Medium | Low | Adult | NA | 0 |
| PRB04099 | BSMSPG | BSMSPGG2 | Medium | Low | Adult | NA | 0 |
| PRB04101 | BSMSPG | BSMSPGG2 | Medium | Low | NA | NA | 0.3 |
| PRB04103 | BSMSPG | BSMSPGG2 | Medium | Low | Adult | NA | 0 |
| PRB04105 | BSMSPG | BSMSPGG2 | Medium | Low | NA | NA | 0 |
| PRB04107 | BSMSPG | BSMSPGG2 | Medium | Low | Adult | NA | 0 |
| PRB04109 | BSMSPG | BSMSPGG2 | Medium | Low | Young | NA | 0 |
| PRB04111 | BSMSPG | BSMSPGG2 | Medium | Low | NA | NA | 0.5 |
| PRB04113 | BSMSPG | BSMSPGG2 | Medium | Low | NA | NA | 0 |
| PRB04115 | BSMSPG | BSMSPGG2 | Medium | Low | NA | NA | 0.7 |
| PRB04117 | BSMSPG | BSMSPGG2 | Medium | Low | Adult | NA | 0 |
| PRB04119 | BSMSPG | BSMSPGG2 | Medium | Low | NA | NA | 0.8 |
| PRB04121 | BSMSPG | BSMSPGG2 | Medium | Low | Adult | NA | 0 |
| PRB04123 | BSMSPG | BSMSPGG2 | Medium | Low | NA | NA | 0.5 |
| PRB04125 | BSMSPG | BSMSPGG2 | Medium | Low | Adult | NA | 0 |
| PRB04127 | BSMSPG | BSMSPGG2 | Medium | Low | NA | NA | 0 |
| PRB04129 | BSMSPG | BSMSPGG2 | Medium | Low | Adult | NA | 3.1 |
| PRB04131 | BSMSPG | BSMSPGG2 | Medium | Low | Adult | NA | 0.3 |
| PRB04133 | BSMSPG | BSMSPGG2 | Medium | Low | NA | NA | 0.4 |
| PRB04135 | BSMSPG | BSMSPGG2 | Medium | Low | Adult | Male | 2.4 |
| PRB04137 | BSMSPG | BSMSPGG2 | Medium | Low | Adult | NA | 0 |
| PRB04139 | BSMSPG | BSMSPGG2 | Medium | Low | Adult | NA | 0 |
| PRB04141 | BSMSPG | BSMSPGG2 | Medium | Low | Adult | NA | 0 |
| PRB04143 | BSMSPG | BSMSPGG2 | Medium | Low | NA | NA | 0.7 |
| PRB04145 | BSMSPG | BSMSPGG2 | Medium | Low | NA | NA | 0 |
| PRB04147 | BSMSPG | BSMSPGG2 | Medium | Low | NA | NA | 0 |
| PRB07133 | BSMSPG | BSMSPGG2 | Medium | Low | NA | NA | NA |
| PRB07135 | BSMSPG | BSMSPGG2 | Medium | Low | Adult | Female | NA |
| PRB07137 | BSMSPG | BSMSPGG2 | Medium | Low | NA | NA | NA |
| PRB07139 | BSMSPG | BSMSPGG2 | Medium | Low | NA | NA | NA |
| PRB07141 | BSMSPG | BSMSPGG2 | Medium | Low | NA | NA | NA |
| PRB07143 | BSMSPG | BSMSPGG2 | Medium | Low | NA | NA | NA |
| PRB07145 | BSMSPG | BSMSPGG2 | Medium | Low | NA | NA | NA |
| PRB07147 | BSMSPG | BSMSPGG2 | Medium | Low | NA | NA | NA |
| PRB07149 | BSMSPG | BSMSPGG2 | Medium | Low | Adult | Male | NA |
| PRB07151 | BSMSPG | BSMSPGG2 | Medium | Low | NA | NA | NA |
| PRB07153 | BSMSPG | BSMSPGG2 | Medium | Low | Adult | Female | NA |
| PRB07155 | BSMSPG | BSMSPGG2 | Medium | Low | NA | NA | NA |
| PRB07157 | BSMSPG | BSMSPGG2 | Medium | Low | NA | NA | NA |
| PRB07159 | BSMSPG | BSMSPGG2 | Medium | Low | NA | NA | NA |
| PRB07161 | BSMSPG | BSMSPGG2 | Medium | Low | NA | NA | NA |
| PRB07163 | BSMSPG | BSMSPGG2 | Medium | Low | NA | NA | NA |
| PRB07165 | BSMSPG | BSMSPGG2 | Medium | Low | NA | NA | NA |
| PRB07167 | BSMSPG | BSMSPGG2 | Medium | Low | NA | NA | NA |
| PRB07169 | BSMSPG | BSMSPGG2 | Medium | Low | NA | NA | NA |
| PRB07171 | BSMSPG | BSMSPGG2 | Medium | Low | NA | NA | NA |
| PRB07173 | BSMSPG | BSMSPGG2 | Medium | Low | NA | NA | NA |
| PRB07175 | BSMSPG | BSMSPGG2 | Medium | Low | NA | NA | NA |
| PRB07177 | BSMSPG | BSMSPGG2 | Medium | Low | NA | NA | NA |
| PRB07179 | BSMSPG | BSMSPGG2 | Medium | Low | NA | NA | NA |
| PRB07181 | BSMSPG | BSMSPGG2 | Medium | Low | Adult | Male | NA |
| PRB07183 | BSMSPG | BSMSPGG2 | Medium | Low | NA | NA | NA |
| PRB07185 | BSMSPG | BSMSPGG2 | Medium | Low | NA | NA | NA |
| PRB07187 | BSMSPG | BSMSPGG2 | Medium | Low | NA | NA | NA |
| PRB07189 | BSMSPG | BSMSPGG2 | Medium | Low | NA | NA | NA |
| PRB07191 | BSMSPG | BSMSPGG2 | Medium | Low | NA | NA | NA |
| PRB07193 | BSMSPG | BSMSPGG2 | Medium | Low | NA | NA | NA |
| PRB07195 | BSMSPG | BSMSPGG2 | Medium | Low | NA | NA | NA |
| PRB07197 | BSMSPG | BSMSPGG2 | Medium | Low | NA | NA | NA |
| PRB07199 | BSMSPG | BSMSPGG2 | Medium | Low | NA | NA | NA |
| PRB07201 | BSMSPG | BSMSPGG2 | Medium | Low | NA | NA | NA |
| PRB07203 | BSMSPG | BSMSPGG2 | Medium | Low | NA | NA | NA |
| PRB07205 | BSMSPG | BSMSPGG2 | Medium | Low | NA | NA | NA |
| PRB07207 | BSMSPG | BSMSPGG2 | Medium | Low | NA | NA | NA |
| PRB07209 | BSMSPG | BSMSPGG2 | Medium | Low | NA | NA | NA |
| PRB07211 | BSMSPG | BSMSPGG2 | Medium | Low | NA | NA | NA |
| PRB07213 | BSMSPG | BSMSPGG2 | Medium | Low | NA | NA | NA |
| PRB07215 | BSMSPG | BSMSPGG2 | Medium | Low | NA | NA | NA |
| PRB07217 | BSMSPG | BSMSPGG2 | Medium | Low | NA | NA | NA |
| PRB07219 | BSMSPG | BSMSPGG2 | Medium | Low | NA | NA | NA |
| PRB07221 | BSMSPG | BSMSPGG2 | Medium | Low | NA | NA | NA |
| PRB07223 | BSMSPG | BSMSPGG2 | Medium | Low | NA | NA | NA |
| PRB07225 | BSMSPG | BSMSPGG2 | Medium | Low | NA | NA | NA |
| PRB07227 | BSMSPG | BSMSPGG2 | Medium | Low | NA | NA | NA |
| PRB07229 | BSMSPG | BSMSPGG2 | Medium | Low | NA | NA | NA |
| PRB03139 | CM | CMG1 | Medium | High | NA | NA | 4.8 |
| PRB03141 | CM | CMG1 | Medium | High | NA | NA | 2.7 |
| PRB03143 | CM | CMG1 | Medium | High | NA | NA | 2.3 |
| PRB03145 | CM | CMG1 | Medium | High | NA | NA | 3.3 |
| PRB03147 | CM | CMG1 | Medium | High | NA | NA | 7 |
| PRB03149 | CM | CMG1 | Medium | High | NA | NA | 9.6 |
| PRB03151 | CM | CMG1 | Medium | High | NA | NA | 1.4 |
| PRB03153 | CM | CMG1 | Medium | High | NA | NA | 14.9 |
| PRB03155 | CM | CMG1 | Medium | High | NA | NA | 6.6 |
| PRB03157 | CM | CMG1 | Medium | High | NA | NA | 5.6 |
| PRB03159 | CM | CMG1 | Medium | High | NA | NA | 4.7 |
| PRB03161 | CM | CMG1 | Medium | High | NA | NA | 3.3 |
| PRB03163 | CM | CMG1 | Medium | High | NA | NA | 6.1 |
| PRB03165 | CM | CMG1 | Medium | High | NA | NA | 12.1 |
| PRB03167 | CM | CMG1 | Medium | High | NA | NA | 1.9 |
| PRB03169 | CM | CMG1 | Medium | High | NA | NA | 3 |
| PRB03171 | CM | CMG1 | Medium | High | NA | NA | 20.6 |
| PRB03173 | CM | CMG1 | Medium | High | NA | NA | 4.8 |
| PRB03175 | CM | CMG1 | Medium | High | NA | NA | 1.8 |
| PRB03177 | CM | CMG1 | Medium | High | NA | NA | NA |
| PRB03179 | CM | CMG1 | Medium | High | NA | NA | 9 |
| PRB03181 | CM | CMG1 | Medium | High | NA | NA | 9.9 |
| PRB03183 | CM | CMG1 | Medium | High | NA | NA | NA |
| PRB03185 | CM | CMG1 | Medium | High | NA | NA | 3.4 |
| PRB03187 | CM | CMG1 | Medium | High | NA | NA | 5.1 |
| PRB03189 | CM | CMG1 | Medium | High | NA | NA | NA |
| PRB03191 | CM | CMG1 | Medium | High | NA | NA | NA |
| PRB03193 | CM | CMG1 | Medium | High | NA | NA | 13.9 |
| PRB03195 | CM | CMG1 | Medium | High | NA | NA | 0.3 |
| PRB03197 | CM | CMG1 | Medium | High | NA | NA | NA |
| PRB03305 | CM | CMG1 | Medium | High | NA | NA | NA |
| PRB03311 | CM | CMG1 | Medium | High | NA | NA | 14.8 |
| PRB03315 | CM | CMG1 | Medium | High | NA | NA | 8.4 |
| PRB03317 | CM | CMG1 | Medium | High | NA | NA | 11 |
| PRB04861 | CM | CMG1 | Medium | High | Adult | Male | NA |
| PRB04867 | CM | CMG1 | Medium | High | Adult | Female | NA |
| PRB04877 | CM | CMG1 | Medium | High | Adult | Female | NA |
| PRB04887 | CM | CMG1 | Medium | High | Adult | Female | NA |
| PRB04943 | CM | CMG1 | Medium | High | Young | Male | NA |
| PRB04953 | CM | CMG1 | Medium | High | Adult | Male | NA |
| PRB08001 | CM | CMG1 | Medium | High | NA | NA | NA |
| PRB08003 | CM | CMG1 | Medium | High | NA | NA | NA |
| PRB08005 | CM | CMG1 | Medium | High | NA | NA | NA |
| PRB08007 | CM | CMG1 | Medium | High | NA | NA | NA |
| PRB08009 | CM | CMG1 | Medium | High | NA | NA | NA |
| PRB08011 | CM | CMG1 | Medium | High | NA | NA | NA |
| PRB08013 | CM | CMG1 | Medium | High | NA | NA | NA |
| PRB08015 | CM | CMG1 | Medium | High | NA | NA | NA |
| PRB08017 | CM | CMG1 | Medium | High | NA | NA | NA |
| PRB08019 | CM | CMG1 | Medium | High | Adult | Male | NA |
| PRB08021 | CM | CMG1 | Medium | High | Adult | Female | NA |
| PRB08023 | CM | CMG1 | Medium | High | Young | Male | NA |
| PRB08025 | CM | CMG1 | Medium | High | Young | Female | NA |
| PRB08027 | CM | CMG1 | Medium | High | NA | NA | NA |
| PRB08029 | CM | CMG1 | Medium | High | NA | NA | NA |
| PRB08031 | CM | CMG1 | Medium | High | NA | NA | NA |
| PRB08033 | CM | CMG1 | Medium | High | NA | NA | NA |
| PRB08035 | CM | CMG1 | Medium | High | NA | NA | NA |
| PRB08037 | CM | CMG1 | Medium | High | NA | NA | NA |
| PRB08039 | CM | CMG1 | Medium | High | NA | NA | NA |
| PRB08041 | CM | CMG1 | Medium | High | NA | NA | NA |
| PRB08043 | CM | CMG1 | Medium | High | NA | NA | NA |
| PRB08045 | CM | CMG1 | Medium | High | NA | NA | NA |
| PRB08047 | CM | CMG1 | Medium | High | NA | NA | NA |
| PRB08049 | CM | CMG1 | Medium | High | NA | NA | NA |
| PRB08051 | CM | CMG1 | Medium | High | NA | NA | NA |
| PRB08053 | CM | CMG1 | Medium | High | NA | NA | NA |
| PRB08055 | CM | CMG1 | Medium | High | NA | NA | NA |
| PRB08057 | CM | CMG1 | Medium | High | NA | NA | NA |
| PRB08059 | CM | CMG1 | Medium | High | NA | NA | NA |
| PRB08061 | CM | CMG1 | Medium | High | NA | NA | NA |
| PRB08063 | CM | CMG1 | Medium | High | NA | NA | NA |
| PRB08065 | CM | CMG1 | Medium | High | NA | NA | NA |
| PRB08067 | CM | CMG1 | Medium | High | NA | NA | NA |
| PRB08069 | CM | CMG1 | Medium | High | NA | NA | NA |
| PRB08071 | CM | CMG1 | Medium | High | NA | NA | NA |
| PRB08073 | CM | CMG1 | Medium | High | NA | NA | NA |
| PRB08075 | CM | CMG1 | Medium | High | NA | NA | NA |
| PRB08077 | CM | CMG1 | Medium | High | Adult | Male | NA |
| PRB08079 | CM | CMG1 | Medium | High | NA | NA | NA |
| PRB08081 | CM | CMG1 | Medium | High | NA | NA | NA |
| PRB08083 | CM | CMG1 | Medium | High | NA | NA | NA |
| PRB08085 | CM | CMG1 | Medium | High | NA | NA | NA |
| PRB08087 | CM | CMG1 | Medium | High | Adult | Male | NA |
| PRB08089 | CM | CMG1 | Medium | High | NA | NA | NA |
| PRB08091 | CM | CMG1 | Medium | High | NA | NA | NA |
| PRB08093 | CM | CMG1 | Medium | High | NA | NA | NA |
| PRB08095 | CM | CMG1 | Medium | High | NA | NA | NA |
| PRB08097 | CM | CMG1 | Medium | High | NA | NA | NA |
| PRB08099 | CM | CMG1 | Medium | High | NA | NA | NA |
| PRB08101 | CM | CMG1 | Medium | High | NA | NA | NA |
| PRB08103 | CM | CMG1 | Medium | High | NA | NA | NA |
| PRB08105 | CM | CMG1 | Medium | High | Adult | Male | NA |
| PRB08107 | CM | CMG1 | Medium | High | NA | NA | NA |
| PRB08109 | CM | CMG1 | Medium | High | NA | NA | NA |
| PRB08111 | CM | CMG1 | Medium | High | NA | NA | NA |
| PRB08113 | CM | CMG1 | Medium | High | NA | NA | NA |
| PRB08115 | CM | CMG1 | Medium | High | NA | NA | NA |
| PRB08117 | CM | CMG1 | Medium | High | NA | NA | NA |
| PRB08119 | CM | CMG1 | Medium | High | NA | NA | NA |
| PRB08121 | CM | CMG1 | Medium | High | Adult | Male | NA |
| PRB08123 | CM | CMG1 | Medium | High | NA | NA | NA |
| PRB08125 | CM | CMG1 | Medium | High | NA | NA | NA |
| PRB08127 | CM | CMG1 | Medium | High | NA | NA | NA |
| PRB08129 | CM | CMG1 | Medium | High | NA | NA | NA |
| PRB08131 | CM | CMG1 | Medium | High | NA | NA | NA |
| PRB08133 | CM | CMG1 | Medium | High | NA | NA | NA |
| PRB08135 | CM | CMG1 | Medium | High | NA | NA | NA |
| PRB08137 | CM | CMG1 | Medium | High | NA | NA | NA |
| PRB08139 | CM | CMG1 | Medium | High | NA | NA | NA |
| PRB08147 | CM | CMG1 | Medium | High | Adult | Male | NA |
| PRB08149 | CM | CMG1 | Medium | High | NA | NA | NA |
| PRB08151 | CM | CMG1 | Medium | High | NA | NA | NA |
| PRB08153 | CM | CMG1 | Medium | High | NA | NA | NA |
| PRB08213 | CM | CMG1 | Medium | High | NA | NA | NA |
| PRB08215 | CM | CMG1 | Medium | High | NA | NA | NA |
| PRB08217 | CM | CMG1 | Medium | High | NA | NA | NA |
| PRB08219 | CM | CMG1 | Medium | High | NA | NA | NA |
| PRB08221 | CM | CMG1 | Medium | High | Young | Male | NA |
| PRB08223 | CM | CMG1 | Medium | High | NA | NA | NA |
| PRB08225 | CM | CMG1 | Medium | High | NA | NA | NA |
| PRB08227 | CM | CMG1 | Medium | High | NA | NA | NA |
| PRB08229 | CM | CMG1 | Medium | High | NA | NA | NA |
| PRB08231 | CM | CMG1 | Medium | High | NA | NA | NA |
| PRB08233 | CM | CMG1 | Medium | High | NA | NA | NA |
| PRB08235 | CM | CMG1 | Medium | High | NA | NA | NA |
| PRB08237 | CM | CMG1 | Medium | High | NA | NA | NA |
| PRB08239 | CM | CMG1 | Medium | High | NA | NA | NA |
| PRB08241 | CM | CMG1 | Medium | High | NA | NA | NA |
| PRB08243 | CM | CMG1 | Medium | High | Adult | Female | NA |
| PRB08245 | CM | CMG1 | Medium | High | NA | NA | NA |
| PRB08247 | CM | CMG1 | Medium | High | Adult | Male | NA |
| PRB08249 | CM | CMG1 | Medium | High | NA | NA | NA |
| PRB08251 | CM | CMG1 | Medium | High | NA | NA | NA |
| PRB08253 | CM | CMG1 | Medium | High | NA | NA | NA |
| PRB08255 | CM | CMG1 | Medium | High | NA | NA | NA |
| PRB08257 | CM | CMG1 | Medium | High | NA | NA | NA |
| PRB08259 | CM | CMG1 | Medium | High | NA | NA | NA |
| PRB08261 | CM | CMG1 | Medium | High | NA | NA | NA |
| PRB08263 | CM | CMG1 | Medium | High | NA | NA | NA |
| PRB08265 | CM | CMG1 | Medium | High | NA | NA | NA |
| PRB08267 | CM | CMG1 | Medium | High | NA | NA | NA |
| PRB08269 | CM | CMG1 | Medium | High | NA | NA | NA |
| PRB08271 | CM | CMG1 | Medium | High | NA | NA | NA |
| PRB08273 | CM | CMG1 | Medium | High | NA | NA | NA |
| PRB08275 | CM | CMG1 | Medium | High | NA | NA | NA |
| PRB08277 | CM | CMG1 | Medium | High | NA | NA | NA |
| PRB08279 | CM | CMG1 | Medium | High | NA | NA | NA |
| PRB08281 | CM | CMG1 | Medium | High | NA | NA | NA |
| PRB08283 | CM | CMG1 | Medium | High | NA | NA | NA |
| PRB08285 | CM | CMG1 | Medium | High | NA | NA | NA |
| PRB08287 | CM | CMG1 | Medium | High | NA | NA | NA |
| PRB08289 | CM | CMG1 | Medium | High | Adult | Female | NA |
| PRB08291 | CM | CMG1 | Medium | High | NA | NA | NA |
| PRB08293 | CM | CMG1 | Medium | High | NA | NA | NA |
| PRB08295 | CM | CMG1 | Medium | High | NA | NA | NA |
| PRB08297 | CM | CMG1 | Medium | High | NA | NA | NA |
| PRB08299 | CM | CMG1 | Medium | High | NA | NA | NA |
| PRB08301 | CM | CMG1 | Medium | High | Adult | Female | NA |
| PRB08303 | CM | CMG1 | Medium | High | NA | NA | NA |
| PRB08305 | CM | CMG1 | Medium | High | NA | NA | NA |
| PRB08307 | CM | CMG1 | Medium | High | NA | NA | NA |
| PRB08309 | CM | CMG1 | Medium | High | NA | NA | NA |
| PRB08311 | CM | CMG1 | Medium | High | NA | NA | NA |
| PRB08313 | CM | CMG1 | Medium | High | NA | NA | NA |
| PRB08315 | CM | CMG1 | Medium | High | NA | NA | NA |
| PRB08317 | CM | CMG1 | Medium | High | NA | NA | NA |
| PRB08319 | CM | CMG1 | Medium | High | NA | NA | NA |
| PRB08321 | CM | CMG1 | Medium | High | NA | NA | NA |
| PRB08323 | CM | CMG1 | Medium | High | NA | NA | NA |
| PRB08325 | CM | CMG1 | Medium | High | NA | NA | NA |
| PRB08327 | CM | CMG1 | Medium | High | NA | NA | NA |
| PRB08329 | CM | CMG1 | Medium | High | NA | NA | NA |
| PRB08331 | CM | CMG1 | Medium | High | NA | NA | NA |
| PRB03321 | CM | CMG2 | Medium | High | NA | NA | 12.9 |
| PRB03323 | CM | CMG2 | Medium | High | NA | NA | 7.7 |
| PRB03325 | CM | CMG2 | Medium | High | NA | NA | 13.4 |
| PRB03329 | CM | CMG2 | Medium | High | NA | NA | 7.1 |
| PRB03331 | CM | CMG2 | Medium | High | NA | NA | NA |
| PRB03333 | CM | CMG2 | Medium | High | NA | NA | 9.4 |
| PRB03335 | CM | CMG2 | Medium | High | NA | NA | 16.3 |
| PRB03337 | CM | CMG2 | Medium | High | NA | NA | 30.9 |
| PRB03339 | CM | CMG2 | Medium | High | NA | NA | 6.1 |
| PRB03341 | CM | CMG2 | Medium | High | NA | NA | 16.3 |
| PRB03343 | CM | CMG2 | Medium | High | NA | NA | 4.4 |
| PRB03345 | CM | CMG2 | Medium | High | NA | NA | 9.4 |
| PRB03347 | CM | CMG2 | Medium | High | NA | NA | 21 |
| PRB03349 | CM | CMG2 | Medium | High | NA | NA | 36.6 |
| PRB03351 | CM | CMG2 | Medium | High | NA | NA | 9.1 |
| PRB03353 | CM | CMG2 | Medium | High | NA | NA | 19.8 |
| PRB03355 | CM | CMG2 | Medium | High | NA | NA | 9.6 |
| PRB03357 | CM | CMG2 | Medium | High | NA | NA | NA |
| PRB03359 | CM | CMG2 | Medium | High | NA | NA | 1.4 |
| PRB04897 | CM | CMG2 | Medium | High | Adult | Male | NA |
| PRB04907 | CM | CMG2 | Medium | High | Adult | Female | NA |
| PRB04917 | CM | CMG2 | Medium | High | Young | Male | NA |
| PRB04927 | CM | CMG2 | Medium | High | Adult | Female | NA |
| PRB04933 | CM | CMG2 | Medium | High | Adult | Female | NA |
| PRB08155 | CM | CMG2 | Medium | High | NA | NA | NA |
| PRB08157 | CM | CMG2 | Medium | High | NA | NA | NA |
| PRB08159 | CM | CMG2 | Medium | High | NA | NA | NA |
| PRB08161 | CM | CMG2 | Medium | High | NA | NA | NA |
| PRB08163 | CM | CMG2 | Medium | High | NA | NA | NA |
| PRB08165 | CM | CMG2 | Medium | High | NA | NA | NA |
| PRB08167 | CM | CMG2 | Medium | High | NA | NA | NA |
| PRB08169 | CM | CMG2 | Medium | High | NA | NA | NA |
| PRB08171 | CM | CMG2 | Medium | High | NA | NA | NA |
| PRB08173 | CM | CMG2 | Medium | High | NA | NA | NA |
| PRB08175 | CM | CMG2 | Medium | High | NA | NA | NA |
| PRB08177 | CM | CMG2 | Medium | High | NA | NA | NA |
| PRB08179 | CM | CMG2 | Medium | High | NA | NA | NA |
| PRB08181 | CM | CMG2 | Medium | High | NA | NA | NA |
| PRB08183 | CM | CMG2 | Medium | High | Adult | Male | NA |
| PRB08185 | CM | CMG2 | Medium | High | NA | NA | NA |
| PRB08187 | CM | CMG2 | Medium | High | NA | NA | NA |
| PRB08189 | CM | CMG2 | Medium | High | NA | NA | NA |
| PRB08191 | CM | CMG2 | Medium | High | NA | NA | NA |
| PRB08193 | CM | CMG2 | Medium | High | NA | NA | NA |
| PRB08195 | CM | CMG2 | Medium | High | NA | NA | NA |
| PRB08197 | CM | CMG2 | Medium | High | NA | NA | NA |
| PRB08199 | CM | CMG2 | Medium | High | NA | NA | NA |
| PRB08201 | CM | CMG2 | Medium | High | NA | NA | NA |
| PRB08203 | CM | CMG2 | Medium | High | NA | NA | NA |
| PRB08205 | CM | CMG2 | Medium | High | NA | NA | NA |
| PRB08207 | CM | CMG2 | Medium | High | NA | NA | NA |
| PRB08209 | CM | CMG2 | Medium | High | NA | NA | NA |
| PRB08211 | CM | CMG2 | Medium | High | NA | NA | NA |
| PRB03361 | CM | CMG3 | Medium | High | NA | NA | 7.8 |
| PRB03363 | CM | CMG3 | Medium | High | NA | NA | 0 |
| PRB03365 | CM | CMG3 | Medium | High | NA | NA | 25 |
| PRB03367 | CM | CMG3 | Medium | High | NA | NA | 5.4 |
| PRB03369 | CM | CMG3 | Medium | High | NA | NA | 3.5 |
| PRB03371 | CM | CMG3 | Medium | High | NA | NA | 6.6 |
| PRB03373 | CM | CMG3 | Medium | High | NA | NA | 2.4 |
| PRB03375 | CM | CMG3 | Medium | High | NA | NA | 7 |
| PRB03377 | CM | CMG3 | Medium | High | NA | NA | NA |
| PRB03379 | CM | CMG3 | Medium | High | NA | NA | NA |
| PRB03381 | CM | CMG3 | Medium | High | NA | NA | 0 |
| PRB03383 | CM | CMG3 | Medium | High | NA | NA | 5.8 |
| PRB03385 | CM | CMG3 | Medium | High | NA | NA | 0 |
| PRB03387 | CM | CMG3 | Medium | High | NA | NA | 1.2 |
| PRB03389 | CM | CMG3 | Medium | High | NA | NA | 2.9 |
| PRB03391 | CM | CMG3 | Medium | High | NA | NA | 0 |
| PRB03393 | CM | CMG3 | Medium | High | NA | NA | NA |
| PRB03395 | CM | CMG3 | Medium | High | NA | NA | 0 |
| PRB03397 | CM | CMG3 | Medium | High | NA | NA | 3.4 |
| PRB03399 | CM | CMG3 | Medium | High | NA | NA | 3.4 |
| PRB08333 | CM | CMG3 | Medium | High | NA | NA | NA |
| PRB08335 | CM | CMG3 | Medium | High | NA | NA | NA |
| PRB08337 | CM | CMG3 | Medium | High | NA | NA | NA |
| PRB08339 | CM | CMG3 | Medium | High | NA | NA | NA |
| PRB08341 | CM | CMG3 | Medium | High | NA | NA | NA |
| PRB08343 | CM | CMG3 | Medium | High | Adult | Male | NA |
| PRB08345 | CM | CMG3 | Medium | High | NA | NA | NA |
| PRB08347 | CM | CMG3 | Medium | High | NA | NA | NA |
| PRB08349 | CM | CMG3 | Medium | High | NA | NA | NA |
| PRB08351 | CM | CMG3 | Medium | High | NA | NA | NA |
| PRB08353 | CM | CMG3 | Medium | High | NA | NA | NA |
| PRB08355 | CM | CMG3 | Medium | High | NA | NA | NA |
| PRB08357 | CM | CMG3 | Medium | High | NA | NA | NA |
| PRB08359 | CM | CMG3 | Medium | High | NA | NA | NA |
| PRB08361 | CM | CMG3 | Medium | High | NA | NA | NA |
| PRB08363 | CM | CMG3 | Medium | High | Adult | Male | NA |
| PRB08365 | CM | CMG3 | Medium | High | NA | NA | NA |
| PRB08367 | CM | CMG3 | Medium | High | Adult | Female | NA |
| PRB08369 | CM | CMG3 | Medium | High | NA | NA | NA |
| PRB08371 | CM | CMG3 | Medium | High | NA | NA | NA |
| PRB08373 | CM | CMG3 | Medium | High | NA | NA | NA |
| PRB08375 | CM | CMG3 | Medium | High | NA | NA | NA |
| PRB08377 | CM | CMG3 | Medium | High | NA | NA | NA |
| PRB08379 | CM | CMG3 | Medium | High | NA | NA | NA |
| PRB08381 | CM | CMG3 | Medium | High | NA | NA | NA |
| PRB08383 | CM | CMG3 | Medium | High | NA | NA | NA |
| PRB08385 | CM | CMG3 | Medium | High | Adult | Male | NA |
| PRB08387 | CM | CMG3 | Medium | High | NA | NA | NA |
| PRB08389 | CM | CMG3 | Medium | High | NA | NA | NA |
| PRB08391 | CM | CMG3 | Medium | High | NA | NA | NA |
| PRB03815 | CMS | CMSG1 | Medium | Medium | Adult | Female | 3.4 |
| PRB03817 | CMS | CMSG1 | Medium | Medium | NA | NA | 0.4 |
| PRB03819 | CMS | CMSG1 | Medium | Medium | NA | NA | 4.7 |
| PRB03821 | CMS | CMSG1 | Medium | Medium | NA | NA | 9 |
| PRB03823 | CMS | CMSG1 | Medium | Medium | NA | NA | 4.7 |
| PRB03825 | CMS | CMSG1 | Medium | Medium | NA | NA | 6.1 |
| PRB03827 | CMS | CMSG1 | Medium | Medium | NA | NA | 6.2 |
| PRB03829 | CMS | CMSG1 | Medium | Medium | NA | NA | 6.9 |
| PRB03831 | CMS | CMSG1 | Medium | Medium | NA | NA | 7.7 |
| PRB03833 | CMS | CMSG1 | Medium | Medium | NA | NA | 6.4 |
| PRB03835 | CMS | CMSG1 | Medium | Medium | NA | NA | 11.3 |
| PRB03837 | CMS | CMSG1 | Medium | Medium | NA | NA | 7.1 |
| PRB03839 | CMS | CMSG1 | Medium | Medium | NA | NA | 4.1 |
| PRB03841 | CMS | CMSG1 | Medium | Medium | Adult | Male | 3.3 |
| PRB03843 | CMS | CMSG1 | Medium | Medium | NA | NA | 11.5 |
| PRB03845 | CMS | CMSG1 | Medium | Medium | Adult | Male | 0 |
| PRB03847 | CMS | CMSG1 | Medium | Medium | NA | NA | 9 |
| PRB03849 | CMS | CMSG1 | Medium | Medium | NA | NA | 25.5 |
| PRB03851 | CMS | CMSG1 | Medium | Medium | NA | NA | 15.2 |
| PRB03853 | CMS | CMSG1 | Medium | Medium | NA | NA | 7.3 |
| PRB03855 | CMS | CMSG1 | Medium | Medium | NA | NA | 5.9 |
| PRB03857 | CMS | CMSG1 | Medium | Medium | NA | NA | 16 |
| PRB03859 | CMS | CMSG1 | Medium | Medium | NA | NA | 60.8 |
| PRB03861 | CMS | CMSG1 | Medium | Medium | NA | NA | 3.3 |
| PRB03863 | CMS | CMSG1 | Medium | Medium | Adult | Female | 24.9 |
| PRB03865 | CMS | CMSG1 | Medium | Medium | NA | NA | 2.5 |
| PRB03867 | CMS | CMSG1 | Medium | Medium | NA | NA | 3.4 |
| PRB03869 | CMS | CMSG1 | Medium | Medium | NA | NA | 6.4 |
| PRB03871 | CMS | CMSG1 | Medium | Medium | Adult | Female | 1.8 |
| PRB03873 | CMS | CMSG1 | Medium | Medium | NA | NA | 2.6 |
| PRB03875 | CMS | CMSG1 | Medium | Medium | NA | NA | 6.5 |
| PRB03877 | CMS | CMSG1 | Medium | Medium | NA | NA | 5.2 |
| PRB03879 | CMS | CMSG1 | Medium | Medium | NA | NA | 0 |
| PRB03881 | CMS | CMSG1 | Medium | Medium | NA | NA | 11.6 |
| PRB03883 | CMS | CMSG1 | Medium | Medium | NA | NA | 7.1 |
| PRB03885 | CMS | CMSG1 | Medium | Medium | NA | NA | 0 |
| PRB03887 | CMS | CMSG1 | Medium | Medium | Adult | Female | 13.7 |
| PRB03889 | CMS | CMSG1 | Medium | Medium | Adult | Female | 0.5 |
| PRB03891 | CMS | CMSG1 | Medium | Medium | NA | NA | 0.7 |
| PRB03893 | CMS | CMSG1 | Medium | Medium | NA | NA | 0 |
| PRB03895 | CMS | CMSG1 | Medium | Medium | NA | NA | 1.7 |
| PRB03897 | CMS | CMSG1 | Medium | Medium | NA | NA | 3.4 |
| PRB03899 | CMS | CMSG1 | Medium | Medium | NA | NA | 2.1 |
| PRB03901 | CMS | CMSG1 | Medium | Medium | Adult | Male | 14.4 |
| PRB03903 | CMS | CMSG1 | Medium | Medium | NA | NA | 7.4 |
| PRB03905 | CMS | CMSG1 | Medium | Medium | NA | NA | 7.6 |
| PRB03907 | CMS | CMSG1 | Medium | Medium | NA | NA | 5.1 |
| PRB03909 | CMS | CMSG1 | Medium | Medium | NA | NA | 3.9 |
| PRB03911 | CMS | CMSG1 | Medium | Medium | NA | NA | 0 |
| PRB09023 | CMS | CMSG1 | Medium | Medium | NA | NA | NA |
| PRB09025 | CMS | CMSG1 | Medium | Medium | NA | NA | NA |
| PRB09027 | CMS | CMSG1 | Medium | Medium | Adult | Female | NA |
| PRB09029 | CMS | CMSG1 | Medium | Medium | NA | NA | NA |
| PRB09031 | CMS | CMSG1 | Medium | Medium | NA | NA | NA |
| PRB09033 | CMS | CMSG1 | Medium | Medium | NA | NA | NA |
| PRB09035 | CMS | CMSG1 | Medium | Medium | NA | NA | NA |
| PRB09037 | CMS | CMSG1 | Medium | Medium | NA | NA | NA |
| PRB09039 | CMS | CMSG1 | Medium | Medium | Adult | Female | NA |
| PRB09041 | CMS | CMSG1 | Medium | Medium | NA | NA | NA |
| PRB09043 | CMS | CMSG1 | Medium | Medium | NA | NA | NA |
| PRB09045 | CMS | CMSG1 | Medium | Medium | NA | NA | NA |
| PRB09047 | CMS | CMSG1 | Medium | Medium | NA | NA | NA |
| PRB09049 | CMS | CMSG1 | Medium | Medium | NA | NA | NA |
| PRB09051 | CMS | CMSG1 | Medium | Medium | NA | NA | NA |
| PRB09053 | CMS | CMSG1 | Medium | Medium | NA | NA | NA |
| PRB09055 | CMS | CMSG1 | Medium | Medium | Adult | Male | NA |
| PRB09057 | CMS | CMSG1 | Medium | Medium | NA | NA | NA |
| PRB09059 | CMS | CMSG1 | Medium | Medium | NA | NA | NA |
| PRB09061 | CMS | CMSG1 | Medium | Medium | NA | NA | NA |
| PRB09063 | CMS | CMSG1 | Medium | Medium | NA | NA | NA |
| PRB09065 | CMS | CMSG1 | Medium | Medium | NA | NA | NA |
| PRB09067 | CMS | CMSG1 | Medium | Medium | Adult | Male | NA |
| PRB09069 | CMS | CMSG1 | Medium | Medium | NA | NA | NA |
| PRB09071 | CMS | CMSG1 | Medium | Medium | NA | NA | NA |
| PRB09073 | CMS | CMSG1 | Medium | Medium | NA | NA | NA |
| PRB09075 | CMS | CMSG1 | Medium | Medium | NA | NA | NA |
| PRB09077 | CMS | CMSG1 | Medium | Medium | NA | NA | NA |
| PRB09079 | CMS | CMSG1 | Medium | Medium | NA | NA | NA |
| PRB09081 | CMS | CMSG1 | Medium | Medium | NA | NA | NA |
| PRB09083 | CMS | CMSG1 | Medium | Medium | NA | NA | NA |
| PRB09085 | CMS | CMSG1 | Medium | Medium | NA | NA | NA |
| PRB09087 | CMS | CMSG1 | Medium | Medium | NA | NA | NA |
| PRB09089 | CMS | CMSG1 | Medium | Medium | NA | NA | NA |
| PRB09091 | CMS | CMSG1 | Medium | Medium | NA | NA | NA |
| PRB09093 | CMS | CMSG1 | Medium | Medium | NA | NA | NA |
| PRB09095 | CMS | CMSG1 | Medium | Medium | NA | NA | NA |
| PRB09097 | CMS | CMSG1 | Medium | Medium | NA | NA | NA |
| PRB09099 | CMS | CMSG1 | Medium | Medium | NA | NA | NA |
| PRB09101 | CMS | CMSG1 | Medium | Medium | NA | NA | NA |
| PRB09103 | CMS | CMSG1 | Medium | Medium | NA | NA | NA |
| PRB09105 | CMS | CMSG1 | Medium | Medium | NA | NA | NA |
| PRB09107 | CMS | CMSG1 | Medium | Medium | NA | NA | NA |
| PRB09109 | CMS | CMSG1 | Medium | Medium | NA | NA | NA |
| PRB09111 | CMS | CMSG1 | Medium | Medium | NA | NA | NA |
| PRB09113 | CMS | CMSG1 | Medium | Medium | NA | NA | NA |
| PRB09115 | CMS | CMSG1 | Medium | Medium | NA | NA | NA |
| PRB09117 | CMS | CMSG1 | Medium | Medium | NA | NA | NA |
| PRB09119 | CMS | CMSG1 | Medium | Medium | NA | NA | NA |
| PRB09121 | CMS | CMSG1 | Medium | Medium | NA | NA | NA |
| PRB09123 | CMS | CMSG1 | Medium | Medium | NA | NA | NA |
| PRB09125 | CMS | CMSG1 | Medium | Medium | NA | NA | NA |
| PRB09127 | CMS | CMSG1 | Medium | Medium | NA | NA | NA |
| PRB09129 | CMS | CMSG1 | Medium | Medium | NA | NA | NA |
| PRB09131 | CMS | CMSG1 | Medium | Medium | NA | NA | NA |
| PRB09133 | CMS | CMSG1 | Medium | Medium | NA | NA | NA |
| PRB09135 | CMS | CMSG1 | Medium | Medium | NA | NA | NA |
| PRB09137 | CMS | CMSG1 | Medium | Medium | NA | NA | NA |
| PRB09139 | CMS | CMSG1 | Medium | Medium | NA | NA | NA |
| PRB09141 | CMS | CMSG1 | Medium | Medium | NA | NA | NA |
| PRB09143 | CMS | CMSG1 | Medium | Medium | NA | NA | NA |
| PRB09145 | CMS | CMSG1 | Medium | Medium | NA | NA | NA |
| PRB09147 | CMS | CMSG1 | Medium | Medium | NA | NA | NA |
| PRB09149 | CMS | CMSG1 | Medium | Medium | NA | NA | NA |
| PRB02349 | DD | DDG1 | High | Medium | NA | NA | NA |
| PRB02351 | DD | DDG1 | High | Medium | NA | NA | 8.3 |
| PRB02353 | DD | DDG1 | High | Medium | NA | NA | 2.5 |
| PRB02355 | DD | DDG1 | High | Medium | NA | NA | 9.8 |
| PRB02357 | DD | DDG1 | High | Medium | Adult | Male | 13.1 |
| PRB02359 | DD | DDG1 | High | Medium | Adult | Female | 23.9 |
| PRB02361 | DD | DDG1 | High | Medium | NA | NA | 3.2 |
| PRB02363 | DD | DDG1 | High | Medium | NA | NA | 10.6 |
| PRB02365 | DD | DDG1 | High | Medium | NA | NA | 13.7 |
| PRB02367 | DD | DDG1 | High | Medium | Adult | Male | 48.3 |
| PRB02369 | DD | DDG1 | High | Medium | Adult | Female | 10.3 |
| PRB02371 | DD | DDG1 | High | Medium | Adult | Female | 22.4 |
| PRB02373 | DD | DDG1 | High | Medium | Adult | Female | 5.3 |
| PRB02375 | DD | DDG1 | High | Medium | NA | NA | NA |
| PRB02377 | DD | DDG1 | High | Medium | NA | NA | 5.6 |
| PRB02379 | DD | DDG1 | High | Medium | NA | NA | NA |
| PRB02381 | DD | DDG1 | High | Medium | NA | NA | 6.7 |
| PRB02383 | DD | DDG1 | High | Medium | NA | NA | 0.4 |
| PRB02385 | DD | DDG1 | High | Medium | NA | NA | 11.3 |
| PRB02387 | DD | DDG1 | High | Medium | NA | NA | 6.7 |
| PRB02389 | DD | DDG1 | High | Medium | NA | NA | 10.9 |
| PRB02391 | DD | DDG1 | High | Medium | Adult | Male | 13.8 |
| PRB02397 | DD | DDG1 | High | Medium | NA | NA | 11.7 |
| PRB02399 | DD | DDG1 | High | Medium | NA | NA | 3.6 |
| PRB02401 | DD | DDG1 | High | Medium | NA | NA | 17.6 |
| PRB02403 | DD | DDG1 | High | Medium | NA | NA | 2.2 |
| PRB02405 | DD | DDG1 | High | Medium | NA | NA | 16.1 |
| PRB02407 | DD | DDG1 | High | Medium | NA | NA | 2.4 |
| PRB02409 | DD | DDG1 | High | Medium | Adult | Female | 19.3 |
| PRB02411 | DD | DDG1 | High | Medium | Adult | Male | 1.6 |
| PRB02413 | DD | DDG1 | High | Medium | NA | NA | 15.4 |
| PRB02415 | DD | DDG1 | High | Medium | NA | NA | 8.2 |
| PRB02417 | DD | DDG1 | High | Medium | Adult | Male | 11.4 |
| PRB03095 | DD | DDG1 | High | Medium | Adult | Female | 0.5 |
| PRB03097 | DD | DDG1 | High | Medium | Adult | Female | 4.3 |
| PRB03099 | DD | DDG1 | High | Medium | Adult | Female | 2.9 |
| PRB03101 | DD | DDG1 | High | Medium | Adult | Female | 2.6 |
| PRB03103 | DD | DDG1 | High | Medium | Young | Female | 2.4 |
| PRB03105 | DD | DDG1 | High | Medium | Adult | Male | 3.2 |
| PRB03107 | DD | DDG1 | High | Medium | Adult | Female | 1.5 |
| PRB03109 | DD | DDG1 | High | Medium | Adult | Female | 4.4 |
| PRB03111 | DD | DDG1 | High | Medium | Adult | Male | 0.6 |
| PRB03113 | DD | DDG1 | High | Medium | NA | NA | 13.8 |
| PRB03115 | DD | DDG1 | High | Medium | Adult | Female | 2.9 |
| PRB03117 | DD | DDG1 | High | Medium | Adult | Male | 0.5 |
| PRB03119 | DD | DDG1 | High | Medium | Adult | Female | 1 |
| PRB03121 | DD | DDG1 | High | Medium | Adult | Female | 25 |
| PRB03123 | DD | DDG1 | High | Medium | Adult | Male | 1.9 |
| PRB03125 | DD | DDG1 | High | Medium | Adult | Female | 2.4 |
| PRB03127 | DD | DDG1 | High | Medium | Adult | Male | 11.2 |
| PRB03129 | DD | DDG1 | High | Medium | Adult | Female | 1.2 |
| PRB03131 | DD | DDG1 | High | Medium | Young | Female | 6.7 |
| PRB03133 | DD | DDG1 | High | Medium | Adult | Male | 8.4 |
| PRB03135 | DD | DDG1 | High | Medium | NA | NA | 12.9 |
| PRB03137 | DD | DDG1 | High | Medium | Adult | Male | 4.1 |
| PRB05225 | DD | DDG1 | High | Medium | Adult | Female | NA |
| PRB05227 | DD | DDG1 | High | Medium | NA | NA | NA |
| PRB05229 | DD | DDG1 | High | Medium | NA | NA | NA |
| PRB05231 | DD | DDG1 | High | Medium | NA | NA | NA |
| PRB05233 | DD | DDG1 | High | Medium | Adult | Male | NA |
| PRB05235 | DD | DDG1 | High | Medium | Adult | Female | NA |
| PRB05237 | DD | DDG1 | High | Medium | NA | NA | NA |
| PRB05239 | DD | DDG1 | High | Medium | NA | NA | NA |
| PRB05241 | DD | DDG1 | High | Medium | NA | NA | NA |
| PRB05243 | DD | DDG1 | High | Medium | NA | NA | NA |
| PRB05245 | DD | DDG1 | High | Medium | NA | NA | NA |
| PRB05247 | DD | DDG1 | High | Medium | NA | NA | NA |
| PRB05249 | DD | DDG1 | High | Medium | NA | NA | NA |
| PRB05251 | DD | DDG1 | High | Medium | Adult | Female | NA |
| PRB05253 | DD | DDG1 | High | Medium | NA | NA | NA |
| PRB05255 | DD | DDG1 | High | Medium | NA | NA | NA |
| PRB05257 | DD | DDG1 | High | Medium | NA | NA | NA |
| PRB05259 | DD | DDG1 | High | Medium | Adult | Female | NA |
| PRB05261 | DD | DDG1 | High | Medium | NA | NA | NA |
| PRB05263 | DD | DDG1 | High | Medium | NA | NA | NA |
| PRB05265 | DD | DDG1 | High | Medium | NA | NA | NA |
| PRB05267 | DD | DDG1 | High | Medium | NA | NA | NA |
| PRB05269 | DD | DDG1 | High | Medium | NA | NA | NA |
| PRB05271 | DD | DDG1 | High | Medium | Adult | Male | NA |
| PRB05273 | DD | DDG1 | High | Medium | NA | NA | NA |
| PRB05275 | DD | DDG1 | High | Medium | NA | NA | NA |
| PRB05277 | DD | DDG1 | High | Medium | Adult | Female | NA |
| PRB05279 | DD | DDG1 | High | Medium | NA | NA | NA |
| PRB05281 | DD | DDG1 | High | Medium | NA | NA | NA |
| PRB05283 | DD | DDG1 | High | Medium | NA | NA | NA |
| PRB05285 | DD | DDG1 | High | Medium | NA | NA | NA |
| PRB05287 | DD | DDG1 | High | Medium | NA | NA | NA |
| PRB05289 | DD | DDG1 | High | Medium | NA | NA | NA |
| PRB05291 | DD | DDG1 | High | Medium | NA | NA | NA |
| PRB05293 | DD | DDG1 | High | Medium | Adult | Female | NA |
| PRB05295 | DD | DDG1 | High | Medium | NA | NA | NA |
| PRB05297 | DD | DDG1 | High | Medium | NA | NA | NA |
| PRB05299 | DD | DDG1 | High | Medium | NA | NA | NA |
| PRB05301 | DD | DDG1 | High | Medium | NA | NA | NA |
| PRB05303 | DD | DDG1 | High | Medium | NA | NA | NA |
| PRB05305 | DD | DDG1 | High | Medium | NA | NA | NA |
| PRB05307 | DD | DDG1 | High | Medium | NA | NA | NA |
| PRB05309 | DD | DDG1 | High | Medium | NA | NA | NA |
| PRB05311 | DD | DDG1 | High | Medium | NA | NA | NA |
| PRB05313 | DD | DDG1 | High | Medium | NA | NA | NA |
| PRB05315 | DD | DDG1 | High | Medium | NA | NA | NA |
| PRB05317 | DD | DDG1 | High | Medium | NA | NA | NA |
| PRB05319 | DD | DDG1 | High | Medium | Adult | Female | NA |
| PRB05321 | DD | DDG1 | High | Medium | NA | NA | NA |
| PRB05323 | DD | DDG1 | High | Medium | NA | NA | NA |
| PRB05325 | DD | DDG1 | High | Medium | NA | NA | NA |
| PRB05327 | DD | DDG1 | High | Medium | NA | NA | NA |
| PRB05329 | DD | DDG1 | High | Medium | NA | NA | NA |
| PRB05331 | DD | DDG1 | High | Medium | NA | NA | NA |
| PRB05333 | DD | DDG1 | High | Medium | NA | NA | NA |
| PRB05335 | DD | DDG1 | High | Medium | NA | NA | NA |
| PRB05337 | DD | DDG1 | High | Medium | NA | NA | NA |
| PRB05339 | DD | DDG1 | High | Medium | NA | NA | NA |
| PRB05341 | DD | DDG1 | High | Medium | NA | NA | NA |
| PRB05343 | DD | DDG1 | High | Medium | NA | NA | NA |
| PRB05345 | DD | DDG1 | High | Medium | NA | NA | NA |
| PRB05347 | DD | DDG1 | High | Medium | NA | NA | NA |
| PRB05349 | DD | DDG1 | High | Medium | NA | NA | NA |
| PRB05351 | DD | DDG1 | High | Medium | NA | NA | NA |
| PRB02419 | DD | DDG2 | High | Medium | Adult | Female | 17.4 |
| PRB02421 | DD | DDG2 | High | Medium | Adult | NA | 15.3 |
| PRB02423 | DD | DDG2 | High | Medium | Adult | Female | 4.2 |
| PRB02425 | DD | DDG2 | High | Medium | Adult | Female | 5.6 |
| PRB02427 | DD | DDG2 | High | Medium | Adult | Female | 1.6 |
| PRB02429 | DD | DDG2 | High | Medium | Young | Male | 18.8 |
| PRB02431 | DD | DDG2 | High | Medium | Young | Male | 7.1 |
| PRB02433 | DD | DDG2 | High | Medium | Adult | Male | 9.3 |
| PRB02435 | DD | DDG2 | High | Medium | Young | Female | NA |
| PRB02437 | DD | DDG2 | High | Medium | Young | Female | 9.2 |
| PRB02439 | DD | DDG2 | High | Medium | Adult | Female | 6.9 |
| PRB02441 | DD | DDG2 | High | Medium | Adult | Female | 6.4 |
| PRB02443 | DD | DDG2 | High | Medium | NA | Male | 3.6 |
| PRB02445 | DD | DDG2 | High | Medium | Adult | Male | 3.1 |
| PRB02447 | DD | DDG2 | High | Medium | Adult | Male | 0.9 |
| PRB02449 | DD | DDG2 | High | Medium | Adult | Female | 3.4 |
| PRB02451 | DD | DDG2 | High | Medium | Adult | Male | 2.7 |
| PRB02453 | DD | DDG2 | High | Medium | Adult | Female | 1.5 |
| PRB02455 | DD | DDG2 | High | Medium | Adult | Female | 0.6 |
| PRB02457 | DD | DDG2 | High | Medium | Adult | Female | 3.7 |
| PRB02459 | DD | DDG2 | High | Medium | Adult | Female | 7.3 |
| PRB02461 | DD | DDG2 | High | Medium | Adult | Female | 8.3 |
| PRB02463 | DD | DDG2 | High | Medium | Adult | Male | 5 |
| PRB02465 | DD | DDG2 | High | Medium | Adult | Male | 6.2 |
| PRB02467 | DD | DDG2 | High | Medium | Young | Female | NA |
| PRB02469 | DD | DDG2 | High | Medium | Adult | Female | 2.7 |
| PRB02471 | DD | DDG2 | High | Medium | Adult | Female | 11.8 |
| PRB02473 | DD | DDG2 | High | Medium | Adult | Female | 1.6 |
| PRB02475 | DD | DDG2 | High | Medium | Adult | Male | 2.8 |
| PRB02477 | DD | DDG2 | High | Medium | Young | Female | 7.7 |
| PRB02479 | DD | DDG2 | High | Medium | NA | NA | 7.3 |
| PRB02481 | DD | DDG2 | High | Medium | Adult | Female | 3 |
| PRB02483 | DD | DDG2 | High | Medium | Adult | Male | 10.5 |
| PRB02485 | DD | DDG2 | High | Medium | Adult | Female | 6.4 |
| PRB02487 | DD | DDG2 | High | Medium | Adult | Female | 13.6 |
| PRB02489 | DD | DDG2 | High | Medium | Adult | NA | 6.5 |
| PRB02491 | DD | DDG2 | High | Medium | Adult | Female | 2.7 |
| PRB02493 | DD | DDG2 | High | Medium | Adult | Female | 2.4 |
| PRB05525 | DD | DDG2 | High | Medium | NA | NA | NA |
| PRB05527 | DD | DDG2 | High | Medium | NA | NA | NA |
| PRB05529 | DD | DDG2 | High | Medium | NA | NA | NA |
| PRB05531 | DD | DDG2 | High | Medium | NA | NA | NA |
| PRB05533 | DD | DDG2 | High | Medium | NA | NA | NA |
| PRB05535 | DD | DDG2 | High | Medium | Adult | Female | NA |
| PRB05537 | DD | DDG2 | High | Medium | Adult | Female | NA |
| PRB05539 | DD | DDG2 | High | Medium | NA | NA | NA |
| PRB05541 | DD | DDG2 | High | Medium | NA | NA | NA |
| PRB05543 | DD | DDG2 | High | Medium | NA | NA | NA |
| PRB05545 | DD | DDG2 | High | Medium | NA | NA | NA |
| PRB05547 | DD | DDG2 | High | Medium | NA | NA | NA |
| PRB05549 | DD | DDG2 | High | Medium | NA | NA | NA |
| PRB05551 | DD | DDG2 | High | Medium | NA | NA | NA |
| PRB05553 | DD | DDG2 | High | Medium | NA | NA | NA |
| PRB05555 | DD | DDG2 | High | Medium | NA | NA | NA |
| PRB05557 | DD | DDG2 | High | Medium | NA | NA | NA |
| PRB05559 | DD | DDG2 | High | Medium | NA | NA | NA |
| PRB05561 | DD | DDG2 | High | Medium | NA | NA | NA |
| PRB05563 | DD | DDG2 | High | Medium | NA | NA | NA |
| PRB05565 | DD | DDG2 | High | Medium | Adult | Male | NA |
| PRB05567 | DD | DDG2 | High | Medium | NA | NA | NA |
| PRB05569 | DD | DDG2 | High | Medium | NA | NA | NA |
| PRB05571 | DD | DDG2 | High | Medium | NA | NA | NA |
| PRB05573 | DD | DDG2 | High | Medium | NA | NA | NA |
| PRB05575 | DD | DDG2 | High | Medium | NA | NA | NA |
| PRB05577 | DD | DDG2 | High | Medium | Adult | Male | NA |
| PRB05579 | DD | DDG2 | High | Medium | NA | NA | NA |
| PRB05581 | DD | DDG2 | High | Medium | NA | NA | NA |
| PRB05583 | DD | DDG2 | High | Medium | NA | NA | NA |
| PRB05585 | DD | DDG2 | High | Medium | NA | NA | NA |
| PRB05587 | DD | DDG2 | High | Medium | NA | NA | NA |
| PRB05589 | DD | DDG2 | High | Medium | NA | NA | NA |
| PRB05591 | DD | DDG2 | High | Medium | NA | NA | NA |
| PRB04503 | KR | KRG1 | Low | Low | NA | NA | 4.2 |
| PRB04505 | KR | KRG1 | Low | Low | NA | NA | 2.3 |
| PRB04507 | KR | KRG1 | Low | Low | Adult | Male | 9.1 |
| PRB04509 | KR | KRG1 | Low | Low | NA | NA | 2.4 |
| PRB04511 | KR | KRG1 | Low | Low | NA | NA | 0 |
| PRB04513 | KR | KRG1 | Low | Low | NA | NA | NA |
| PRB04515 | KR | KRG1 | Low | Low | Adult | Female | 3.4 |
| PRB04517 | KR | KRG1 | Low | Low | NA | NA | NA |
| PRB04519 | KR | KRG1 | Low | Low | NA | NA | 4.3 |
| PRB04521 | KR | KRG1 | Low | Low | NA | NA | 3.2 |
| PRB04523 | KR | KRG1 | Low | Low | NA | NA | 4.9 |
| PRB04525 | KR | KRG1 | Low | Low | NA | NA | 4.6 |
| PRB04527 | KR | KRG1 | Low | Low | Adult | Female | NA |
| PRB04529 | KR | KRG1 | Low | Low | NA | NA | 1.7 |
| PRB04531 | KR | KRG1 | Low | Low | NA | NA | 11.7 |
| PRB04533 | KR | KRG1 | Low | Low | NA | NA | 2.4 |
| PRB04535 | KR | KRG1 | Low | Low | NA | NA | 3.3 |
| PRB04537 | KR | KRG1 | Low | Low | NA | NA | NA |
| PRB04539 | KR | KRG1 | Low | Low | NA | NA | NA |
| PRB04541 | KR | KRG1 | Low | Low | NA | NA | NA |
| PRB04543 | KR | KRG1 | Low | Low | NA | NA | NA |
| PRB04545 | KR | KRG1 | Low | Low | NA | NA | 3.8 |
| PRB04547 | KR | KRG1 | Low | Low | NA | NA | 2.7 |
| PRB04549 | KR | KRG1 | Low | Low | NA | NA | 29.1 |
| PRB04551 | KR | KRG1 | Low | Low | NA | NA | 11.9 |
| PRB09683 | KR | KRG1 | Low | Low | Adult | Male | NA |
| PRB09685 | KR | KRG1 | Low | Low | Adult | Female | NA |
| PRB09687 | KR | KRG1 | Low | Low | Adult | Female | NA |
| PRB09689 | KR | KRG1 | Low | Low | Adult | Male | NA |
| PRB09691 | KR | KRG1 | Low | Low | Adult | Female | NA |
| PRB09693 | KR | KRG1 | Low | Low | Adult | Male | NA |
| PRB09695 | KR | KRG1 | Low | Low | Adult | Female | NA |
| PRB09697 | KR | KRG1 | Low | Low | Adult | Male | NA |
| PRB09699 | KR | KRG1 | Low | Low | Adult | Male | NA |
| PRB09701 | KR | KRG1 | Low | Low | Adult | Female | NA |
| PRB09703 | KR | KRG1 | Low | Low | Young | Female | NA |
| PRB09705 | KR | KRG1 | Low | Low | Adult | Male | NA |
| PRB09707 | KR | KRG1 | Low | Low | Adult | Female | NA |
| PRB09709 | KR | KRG1 | Low | Low | Adult | Male | NA |
| PRB09711 | KR | KRG1 | Low | Low | Adult | Female | NA |
| PRB09713 | KR | KRG1 | Low | Low | Adult | Male | NA |
| PRB09715 | KR | KRG1 | Low | Low | Adult | Male | NA |
| PRB09717 | KR | KRG1 | Low | Low | Adult | Female | NA |
| PRB09719 | KR | KRG1 | Low | Low | Adult | Male | NA |
| PRB09721 | KR | KRG1 | Low | Low | Adult | Female | NA |
| PRB09723 | KR | KRG1 | Low | Low | Adult | Male | NA |
| PRB09725 | KR | KRG1 | Low | Low | Adult | Male | NA |
| PRB09727 | KR | KRG1 | Low | Low | Adult | Female | NA |
| PRB09729 | KR | KRG1 | Low | Low | Adult | Female | NA |
| PRB09731 | KR | KRG1 | Low | Low | Adult | Female | NA |
| PRB09733 | KR | KRG1 | Low | Low | Adult | Male | NA |
| PRB09735 | KR | KRG1 | Low | Low | Adult | Female | NA |
| PRB09737 | KR | KRG1 | Low | Low | Young | Female | NA |
| PRB09739 | KR | KRG1 | Low | Low | Adult | Male | NA |
| PRB09741 | KR | KRG1 | Low | Low | Adult | Female | NA |
| PRB09743 | KR | KRG1 | Low | Low | Adult | Male | NA |
| PRB09745 | KR | KRG1 | Low | Low | Adult | Female | NA |
| PRB09747 | KR | KRG1 | Low | Low | Adult | Female | NA |
| PRB09749 | KR | KRG1 | Low | Low | Adult | Male | NA |
| PRB09751 | KR | KRG1 | Low | Low | Adult | Male | NA |
| PRB09753 | KR | KRG1 | Low | Low | Adult | Male | NA |
| PRB09755 | KR | KRG1 | Low | Low | Adult | Female | NA |
| PRB09757 | KR | KRG1 | Low | Low | Adult | Male | NA |
| PRB09759 | KR | KRG1 | Low | Low | Adult | Female | NA |
| PRB09761 | KR | KRG1 | Low | Low | Adult | Male | NA |
| PRB09763 | KR | KRG1 | Low | Low | Adult | Female | NA |
| PRB09765 | KR | KRG1 | Low | Low | Adult | Female | NA |
| PRB09767 | KR | KRG1 | Low | Low | Adult | Male | NA |
| PRB09769 | KR | KRG1 | Low | Low | Adult | Female | NA |
| PRB04553 | KR | KRG2 | Low | Low | NA | NA | 12.7 |
| PRB04555 | KR | KRG2 | Low | Low | NA | NA | NA |
| PRB04557 | KR | KRG2 | Low | Low | NA | NA | 18.9 |
| PRB04559 | KR | KRG2 | Low | Low | Adult | Male | 8.5 |
| PRB04561 | KR | KRG2 | Low | Low | NA | NA | 7.3 |
| PRB04563 | KR | KRG2 | Low | Low | NA | NA | 8.7 |
| PRB04565 | KR | KRG2 | Low | Low | NA | NA | 9.4 |
| PRB04567 | KR | KRG2 | Low | Low | NA | NA | 6.8 |
| PRB04569 | KR | KRG2 | Low | Low | NA | NA | 6.8 |
| PRB04571 | KR | KRG2 | Low | Low | NA | NA | 6.6 |
| PRB04573 | KR | KRG2 | Low | Low | NA | NA | 14.1 |
| PRB04575 | KR | KRG2 | Low | Low | NA | NA | 3.4 |
| PRB04577 | KR | KRG2 | Low | Low | NA | NA | 13.5 |
| PRB04579 | KR | KRG2 | Low | Low | NA | NA | 34.1 |
| PRB04581 | KR | KRG2 | Low | Low | NA | NA | 5.5 |
| PRB04583 | KR | KRG2 | Low | Low | NA | NA | NA |
| PRB04585 | KR | KRG2 | Low | Low | NA | NA | 8.5 |
| PRB04587 | KR | KRG2 | Low | Low | NA | NA | 25 |
| PRB04589 | KR | KRG2 | Low | Low | NA | NA | 8.5 |
| PRB04591 | KR | KRG2 | Low | Low | NA | NA | 4.7 |
| PRB04593 | KR | KRG2 | Low | Low | NA | NA | 0.4 |
| PRB04595 | KR | KRG2 | Low | Low | NA | NA | 2.9 |
| PRB04597 | KR | KRG2 | Low | Low | NA | NA | 17.2 |
| PRB04599 | KR | KRG2 | Low | Low | Adult | Female | 4.3 |
| PRB04601 | KR | KRG2 | Low | Low | NA | NA | 4.9 |
| PRB04603 | KR | KRG2 | Low | Low | NA | NA | 24.8 |
| PRB04605 | KR | KRG2 | Low | Low | NA | NA | 6.1 |
| PRB04607 | KR | KRG2 | Low | Low | NA | NA | 5.2 |
| PRB07613 | KS | KSG1 | Medium | Medium | Adult | Male | NA |
| PRB07615 | KS | KSG1 | Medium | Medium | NA | NA | NA |
| PRB07617 | KS | KSG1 | Medium | Medium | Adult | Female | NA |
| PRB07619 | KS | KSG1 | Medium | Medium | NA | NA | NA |
| PRB07621 | KS | KSG1 | Medium | Medium | NA | NA | NA |
| PRB07623 | KS | KSG1 | Medium | Medium | NA | NA | NA |
| PRB07625 | KS | KSG1 | Medium | Medium | NA | NA | NA |
| PRB07627 | KS | KSG1 | Medium | Medium | NA | NA | NA |
| PRB07699 | KS | KSG1 | Medium | Medium | NA | NA | NA |
| PRB07701 | KS | KSG1 | Medium | Medium | NA | NA | NA |
| PRB07703 | KS | KSG1 | Medium | Medium | NA | NA | NA |
| PRB07705 | KS | KSG1 | Medium | Medium | NA | NA | NA |
| PRB07707 | KS | KSG1 | Medium | Medium | NA | NA | NA |
| PRB07709 | KS | KSG1 | Medium | Medium | NA | NA | NA |
| PRB07711 | KS | KSG1 | Medium | Medium | NA | NA | NA |
| PRB07713 | KS | KSG1 | Medium | Medium | NA | NA | NA |
| PRB07715 | KS | KSG1 | Medium | Medium | NA | NA | NA |
| PRB07717 | KS | KSG1 | Medium | Medium | NA | NA | NA |
| PRB07719 | KS | KSG1 | Medium | Medium | NA | NA | NA |
| PRB07721 | KS | KSG1 | Medium | Medium | NA | NA | NA |
| PRB07723 | KS | KSG1 | Medium | Medium | NA | NA | NA |
| PRB07725 | KS | KSG1 | Medium | Medium | NA | NA | NA |
| PRB07727 | KS | KSG1 | Medium | Medium | NA | NA | NA |
| PRB07729 | KS | KSG1 | Medium | Medium | NA | NA | NA |
| PRB07731 | KS | KSG1 | Medium | Medium | NA | NA | NA |
| PRB07733 | KS | KSG1 | Medium | Medium | NA | NA | NA |
| PRB07735 | KS | KSG1 | Medium | Medium | NA | NA | NA |
| PRB07737 | KS | KSG1 | Medium | Medium | NA | NA | NA |
| PRB07739 | KS | KSG1 | Medium | Medium | NA | NA | NA |
| PRB07741 | KS | KSG1 | Medium | Medium | NA | NA | NA |
| PRB07743 | KS | KSG1 | Medium | Medium | NA | NA | NA |
| PRB07745 | KS | KSG1 | Medium | Medium | Adult | Male | NA |
| PRB07747 | KS | KSG1 | Medium | Medium | NA | NA | NA |
| PRB07749 | KS | KSG1 | Medium | Medium | NA | NA | NA |
| PRB07751 | KS | KSG1 | Medium | Medium | NA | NA | NA |
| PRB07753 | KS | KSG1 | Medium | Medium | NA | NA | NA |
| PRB07755 | KS | KSG1 | Medium | Medium | Adult | Female | NA |
| PRB07757 | KS | KSG1 | Medium | Medium | NA | NA | NA |
| PRB07759 | KS | KSG1 | Medium | Medium | NA | NA | NA |
| PRB07761 | KS | KSG1 | Medium | Medium | NA | NA | NA |
| PRB07763 | KS | KSG1 | Medium | Medium | NA | NA | NA |
| PRB07765 | KS | KSG1 | Medium | Medium | NA | NA | NA |
| PRB07767 | KS | KSG1 | Medium | Medium | NA | NA | NA |
| PRB07769 | KS | KSG1 | Medium | Medium | NA | NA | NA |
| PRB07771 | KS | KSG1 | Medium | Medium | NA | NA | NA |
| PRB07775 | KS | KSG1 | Medium | Medium | NA | NA | NA |
| PRB07777 | KS | KSG1 | Medium | Medium | NA | NA | NA |
| PRB07779 | KS | KSG1 | Medium | Medium | Adult | Female | NA |
| PRB07781 | KS | KSG1 | Medium | Medium | NA | NA | NA |
| PRB07783 | KS | KSG1 | Medium | Medium | NA | NA | NA |
| PRB07785 | KS | KSG1 | Medium | Medium | Adult | Male | NA |
| PRB07787 | KS | KSG1 | Medium | Medium | NA | NA | NA |
| PRB07789 | KS | KSG1 | Medium | Medium | NA | NA | NA |
| PRB07791 | KS | KSG1 | Medium | Medium | NA | NA | NA |
| PRB07793 | KS | KSG1 | Medium | Medium | NA | NA | NA |
| PRB07795 | KS | KSG1 | Medium | Medium | NA | NA | NA |
| PRB07797 | KS | KSG1 | Medium | Medium | NA | NA | NA |
| PRB07799 | KS | KSG1 | Medium | Medium | NA | NA | NA |
| PRB07801 | KS | KSG1 | Medium | Medium | NA | NA | NA |
| PRB07803 | KS | KSG1 | Medium | Medium | NA | NA | NA |
| PRB07805 | KS | KSG1 | Medium | Medium | NA | NA | NA |
| PRB07807 | KS | KSG1 | Medium | Medium | NA | NA | NA |
| PRB07809 | KS | KSG1 | Medium | Medium | NA | NA | NA |
| PRB07811 | KS | KSG1 | Medium | Medium | NA | NA | NA |
| PRB07813 | KS | KSG1 | Medium | Medium | NA | NA | NA |
| PRB07815 | KS | KSG1 | Medium | Medium | NA | NA | NA |
| PRB07817 | KS | KSG1 | Medium | Medium | NA | NA | NA |
| PRB07819 | KS | KSG1 | Medium | Medium | NA | NA | NA |
| PRB07821 | KS | KSG1 | Medium | Medium | NA | NA | NA |
| PRB07823 | KS | KSG1 | Medium | Medium | NA | NA | NA |
| PRB07825 | KS | KSG1 | Medium | Medium | NA | NA | NA |
| PRB07827 | KS | KSG1 | Medium | Medium | NA | NA | NA |
| PRB07829 | KS | KSG1 | Medium | Medium | NA | NA | NA |
| PRB07831 | KS | KSG1 | Medium | Medium | NA | NA | NA |
| PRB07833 | KS | KSG1 | Medium | Medium | NA | NA | NA |
| PRB07835 | KS | KSG1 | Medium | Medium | NA | NA | NA |
| PRB07837 | KS | KSG1 | Medium | Medium | NA | NA | NA |
| PRB07839 | KS | KSG1 | Medium | Medium | NA | NA | NA |
| PRB07841 | KS | KSG1 | Medium | Medium | NA | NA | NA |
| PRB07843 | KS | KSG1 | Medium | Medium | NA | NA | NA |
| PRB07845 | KS | KSG1 | Medium | Medium | NA | NA | NA |
| PRB07847 | KS | KSG1 | Medium | Medium | NA | NA | NA |
| PRB07849 | KS | KSG1 | Medium | Medium | NA | NA | NA |
| PRB07851 | KS | KSG1 | Medium | Medium | NA | NA | NA |
| PRB07853 | KS | KSG1 | Medium | Medium | NA | NA | NA |
| PRB07855 | KS | KSG1 | Medium | Medium | NA | NA | NA |
| PRB07857 | KS | KSG1 | Medium | Medium | NA | NA | NA |
| PRB07859 | KS | KSG1 | Medium | Medium | NA | NA | NA |
| PRB07861 | KS | KSG1 | Medium | Medium | NA | NA | NA |
| PRB07863 | KS | KSG1 | Medium | Medium | NA | NA | NA |
| PRB07895 | KS | KSG1 | Medium | Medium | NA | NA | NA |
| PRB07897 | KS | KSG1 | Medium | Medium | NA | NA | NA |
| PRB07899 | KS | KSG1 | Medium | Medium | NA | NA | NA |
| PRB07901 | KS | KSG1 | Medium | Medium | NA | NA | NA |
| PRB07903 | KS | KSG1 | Medium | Medium | NA | NA | NA |
| PRB07905 | KS | KSG1 | Medium | Medium | NA | NA | NA |
| PRB07907 | KS | KSG1 | Medium | Medium | NA | NA | NA |
| PRB07909 | KS | KSG1 | Medium | Medium | Adult | Male | NA |
| PRB07911 | KS | KSG1 | Medium | Medium | NA | NA | NA |
| PRB07913 | KS | KSG1 | Medium | Medium | NA | NA | NA |
| PRB07915 | KS | KSG1 | Medium | Medium | Adult | Female | NA |
| PRB07917 | KS | KSG1 | Medium | Medium | NA | NA | NA |
| PRB07919 | KS | KSG1 | Medium | Medium | NA | NA | NA |
| PRB07921 | KS | KSG1 | Medium | Medium | Adult | Female | NA |
| PRB07923 | KS | KSG1 | Medium | Medium | Adult | Male | NA |
| PRB07925 | KS | KSG1 | Medium | Medium | NA | NA | NA |
| PRB07927 | KS | KSG1 | Medium | Medium | NA | NA | NA |
| PRB07929 | KS | KSG1 | Medium | Medium | NA | NA | NA |
| PRB07931 | KS | KSG1 | Medium | Medium | NA | NA | NA |
| PRB07933 | KS | KSG1 | Medium | Medium | NA | NA | NA |
| PRB07935 | KS | KSG1 | Medium | Medium | NA | NA | NA |
| PRB07937 | KS | KSG1 | Medium | Medium | NA | NA | NA |
| PRB07939 | KS | KSG1 | Medium | Medium | NA | NA | NA |
| PRB07941 | KS | KSG1 | Medium | Medium | NA | NA | NA |
| PRB07943 | KS | KSG1 | Medium | Medium | NA | NA | NA |
| PRB07945 | KS | KSG1 | Medium | Medium | NA | NA | NA |
| PRB07947 | KS | KSG1 | Medium | Medium | Adult | Male | NA |
| PRB07949 | KS | KSG1 | Medium | Medium | NA | NA | NA |
| PRB07951 | KS | KSG1 | Medium | Medium | NA | NA | NA |
| PRB07953 | KS | KSG1 | Medium | Medium | NA | NA | NA |
| PRB07955 | KS | KSG1 | Medium | Medium | NA | NA | NA |
| PRB07957 | KS | KSG1 | Medium | Medium | NA | NA | NA |
| PRB07959 | KS | KSG1 | Medium | Medium | NA | NA | NA |
| PRB07961 | KS | KSG1 | Medium | Medium | NA | NA | NA |
| PRB07963 | KS | KSG1 | Medium | Medium | NA | NA | NA |
| PRB07965 | KS | KSG1 | Medium | Medium | NA | NA | NA |
| PRB07967 | KS | KSG1 | Medium | Medium | NA | NA | NA |
| PRB07969 | KS | KSG1 | Medium | Medium | NA | NA | NA |
| PRB07971 | KS | KSG1 | Medium | Medium | NA | NA | NA |
| PRB07629 | KS | KSG2 | Medium | Medium | Adult | Male | NA |
| PRB07631 | KS | KSG2 | Medium | Medium | NA | NA | NA |
| PRB07633 | KS | KSG2 | Medium | Medium | Adult | Female | NA |
| PRB07635 | KS | KSG2 | Medium | Medium | NA | NA | NA |
| PRB07637 | KS | KSG2 | Medium | Medium | NA | NA | NA |
| PRB07639 | KS | KSG2 | Medium | Medium | NA | NA | NA |
| PRB07641 | KS | KSG2 | Medium | Medium | NA | NA | NA |
| PRB07643 | KS | KSG2 | Medium | Medium | NA | NA | NA |
| PRB07645 | KS | KSG2 | Medium | Medium | NA | NA | NA |
| PRB07647 | KS | KSG2 | Medium | Medium | Adult | Female | NA |
| PRB07649 | KS | KSG2 | Medium | Medium | Adult | Male | NA |
| PRB07651 | KS | KSG2 | Medium | Medium | NA | NA | NA |
| PRB07653 | KS | KSG2 | Medium | Medium | NA | NA | NA |
| PRB07655 | KS | KSG2 | Medium | Medium | NA | NA | NA |
| PRB07657 | KS | KSG2 | Medium | Medium | NA | NA | NA |
| PRB07659 | KS | KSG2 | Medium | Medium | NA | NA | NA |
| PRB07697 | KS | KSG2 | Medium | Medium | NA | NA | NA |
| PRB07865 | KS | KSG2 | Medium | Medium | NA | NA | NA |
| PRB07867 | KS | KSG2 | Medium | Medium | NA | NA | NA |
| PRB07869 | KS | KSG2 | Medium | Medium | NA | NA | NA |
| PRB07871 | KS | KSG2 | Medium | Medium | NA | NA | NA |
| PRB07873 | KS | KSG2 | Medium | Medium | NA | NA | NA |
| PRB07875 | KS | KSG2 | Medium | Medium | NA | NA | NA |
| PRB07877 | KS | KSG2 | Medium | Medium | NA | NA | NA |
| PRB07879 | KS | KSG2 | Medium | Medium | NA | NA | NA |
| PRB07881 | KS | KSG2 | Medium | Medium | NA | NA | NA |
| PRB07883 | KS | KSG2 | Medium | Medium | NA | NA | NA |
| PRB07885 | KS | KSG2 | Medium | Medium | NA | NA | NA |
| PRB07887 | KS | KSG2 | Medium | Medium | NA | NA | NA |
| PRB07889 | KS | KSG2 | Medium | Medium | NA | NA | NA |
| PRB07891 | KS | KSG2 | Medium | Medium | NA | NA | NA |
| PRB07893 | KS | KSG2 | Medium | Medium | NA | NA | NA |
| PRB03401 | KS | KSG3 | Medium | Medium | NA | NA | 0 |
| PRB03403 | KS | KSG3 | Medium | Medium | NA | NA | 2.2 |
| PRB03405 | KS | KSG3 | Medium | Medium | NA | NA | 1.9 |
| PRB03407 | KS | KSG3 | Medium | Medium | NA | NA | 27.3 |
| PRB03409 | KS | KSG3 | Medium | Medium | NA | NA | 4.8 |
| PRB03411 | KS | KSG3 | Medium | Medium | NA | NA | 0.2 |
| PRB03413 | KS | KSG3 | Medium | Medium | NA | NA | 0.3 |
| PRB03415 | KS | KSG3 | Medium | Medium | NA | NA | 1.9 |
| PRB03417 | KS | KSG3 | Medium | Medium | NA | NA | 0 |
| PRB03419 | KS | KSG3 | Medium | Medium | NA | NA | NA |
| PRB03421 | KS | KSG3 | Medium | Medium | Young | Female | NA |
| PRB03423 | KS | KSG3 | Medium | Medium | NA | NA | 0 |
| PRB03425 | KS | KSG3 | Medium | Medium | NA | NA | 4.5 |
| PRB03427 | KS | KSG3 | Medium | Medium | NA | NA | 3.7 |
| PRB03429 | KS | KSG3 | Medium | Medium | Adult | Male | 0.6 |
| PRB03431 | KS | KSG3 | Medium | Medium | Adult | Male | 2.5 |
| PRB03433 | KS | KSG3 | Medium | Medium | NA | NA | 0 |
| PRB03435 | KS | KSG3 | Medium | Medium | NA | NA | 0.4 |
| PRB03437 | KS | KSG3 | Medium | Medium | NA | NA | 2.4 |
| PRB03439 | KS | KSG3 | Medium | Medium | NA | NA | 0.9 |
| PRB03441 | KS | KSG3 | Medium | Medium | NA | NA | 5.4 |
| PRB03913 | MES | MESG1 | Low | Low | NA | NA | 5.1 |
| PRB03915 | MES | MESG1 | Low | Low | NA | NA | 1.5 |
| PRB03917 | MES | MESG1 | Low | Low | NA | NA | 2.4 |
| PRB03919 | MES | MESG1 | Low | Low | NA | NA | 0 |
| PRB03921 | MES | MESG1 | Low | Low | NA | NA | 7 |
| PRB03923 | MES | MESG1 | Low | Low | NA | NA | 6.9 |
| PRB03925 | MES | MESG1 | Low | Low | NA | NA | 1.1 |
| PRB03927 | MES | MESG1 | Low | Low | NA | NA | 1.9 |
| PRB03929 | MES | MESG1 | Low | Low | NA | NA | 2.2 |
| PRB03931 | MES | MESG1 | Low | Low | NA | NA | 11.3 |
| PRB03933 | MES | MESG1 | Low | Low | NA | Male | 7.5 |
| PRB03935 | MES | MESG1 | Low | Low | NA | NA | 1.4 |
| PRB03937 | MES | MESG1 | Low | Low | NA | Male | 1.7 |
| PRB03939 | MES | MESG1 | Low | Low | NA | NA | 0.6 |
| PRB03941 | MES | MESG1 | Low | Low | Adult | Female | 6.3 |
| PRB09167 | MES | MESG1 | Low | Low | NA | NA | NA |
| PRB09169 | MES | MESG1 | Low | Low | NA | NA | NA |
| PRB09171 | MES | MESG1 | Low | Low | Adult | Female | NA |
| PRB09173 | MES | MESG1 | Low | Low | NA | NA | NA |
| PRB09175 | MES | MESG1 | Low | Low | NA | NA | NA |
| PRB09177 | MES | MESG1 | Low | Low | NA | NA | NA |
| PRB09179 | MES | MESG1 | Low | Low | NA | NA | NA |
| PRB09181 | MES | MESG1 | Low | Low | NA | NA | NA |
| PRB09183 | MES | MESG1 | Low | Low | Adult | Male | NA |
| PRB09185 | MES | MESG1 | Low | Low | NA | NA | NA |
| PRB09187 | MES | MESG1 | Low | Low | NA | NA | NA |
| PRB09189 | MES | MESG1 | Low | Low | NA | NA | NA |
| PRB09191 | MES | MESG1 | Low | Low | NA | NA | NA |
| PRB09193 | MES | MESG1 | Low | Low | NA | NA | NA |
| PRB09195 | MES | MESG1 | Low | Low | NA | NA | NA |
| PRB09197 | MES | MESG1 | Low | Low | NA | NA | NA |
| PRB09199 | MES | MESG1 | Low | Low | NA | NA | NA |
| PRB09201 | MES | MESG1 | Low | Low | NA | NA | NA |
| PRB09203 | MES | MESG1 | Low | Low | NA | NA | NA |
| PRB09205 | MES | MESG1 | Low | Low | NA | NA | NA |
| PRB09207 | MES | MESG1 | Low | Low | NA | NA | NA |
| PRB09209 | MES | MESG1 | Low | Low | NA | NA | NA |
| PRB09211 | MES | MESG1 | Low | Low | NA | NA | NA |
| PRB09213 | MES | MESG1 | Low | Low | NA | NA | NA |
| PRB09215 | MES | MESG1 | Low | Low | NA | NA | NA |
| PRB09217 | MES | MESG1 | Low | Low | NA | NA | NA |
| PRB09219 | MES | MESG1 | Low | Low | NA | NA | NA |
| PRB09221 | MES | MESG1 | Low | Low | NA | NA | NA |
| PRB09223 | MES | MESG1 | Low | Low | NA | NA | NA |
| PRB09225 | MES | MESG1 | Low | Low | NA | NA | NA |
| PRB09227 | MES | MESG1 | Low | Low | NA | NA | NA |
| PRB09229 | MES | MESG1 | Low | Low | NA | NA | NA |
| PRB09231 | MES | MESG1 | Low | Low | Adult | Male | NA |
| PRB09233 | MES | MESG1 | Low | Low | NA | NA | NA |
| PRB09235 | MES | MESG1 | Low | Low | NA | NA | NA |
| PRB09237 | MES | MESG1 | Low | Low | NA | NA | NA |
| PRB09239 | MES | MESG1 | Low | Low | NA | NA | NA |
| PRB09241 | MES | MESG1 | Low | Low | NA | NA | NA |
| PRB09243 | MES | MESG1 | Low | Low | NA | NA | NA |
| PRB09245 | MES | MESG1 | Low | Low | NA | NA | NA |
| PRB09247 | MES | MESG1 | Low | Low | NA | NA | NA |
| PRB09249 | MES | MESG1 | Low | Low | NA | NA | NA |
| PRB09251 | MES | MESG1 | Low | Low | NA | NA | NA |
| PRB09253 | MES | MESG1 | Low | Low | NA | NA | NA |
| PRB09255 | MES | MESG1 | Low | Low | NA | NA | NA |
| PRB09257 | MES | MESG1 | Low | Low | NA | NA | NA |
| PRB09259 | MES | MESG1 | Low | Low | NA | NA | NA |
| PRB04657 | ODD | ODDG1 | High | Low | NA | NA | 2.4 |
| PRB04659 | ODD | ODDG1 | High | Low | NA | NA | 8.2 |
| PRB04661 | ODD | ODDG1 | High | Low | NA | NA | 4.6 |
| PRB04663 | ODD | ODDG1 | High | Low | NA | NA | 6.7 |
| PRB04665 | ODD | ODDG1 | High | Low | NA | NA | 5.2 |
| PRB04667 | ODD | ODDG1 | High | Low | NA | NA | 25.8 |
| PRB04669 | ODD | ODDG1 | High | Low | NA | NA | 9.2 |
| PRB04671 | ODD | ODDG1 | High | Low | NA | NA | 24.8 |
| PRB04673 | ODD | ODDG1 | High | Low | NA | NA | 5.2 |
| PRB04675 | ODD | ODDG1 | High | Low | NA | NA | 23.2 |
| PRB04677 | ODD | ODDG1 | High | Low | NA | NA | 13.8 |
| PRB04679 | ODD | ODDG1 | High | Low | NA | NA | 19.1 |
| PRB04681 | ODD | ODDG1 | High | Low | NA | NA | 19.8 |
| PRB04683 | ODD | ODDG1 | High | Low | NA | NA | 6.2 |
| PRB04685 | ODD | ODDG1 | High | Low | NA | NA | 11.1 |
| PRB04687 | ODD | ODDG1 | High | Low | NA | NA | 49.7 |
| PRB04689 | ODD | ODDG1 | High | Low | NA | NA | 2.1 |
| PRB04691 | ODD | ODDG1 | High | Low | NA | NA | 4.2 |
| PRB04693 | ODD | ODDG1 | High | Low | NA | Male | 3.8 |
| PRB04695 | ODD | ODDG1 | High | Low | Adult | Female | 6.6 |
| PRB04697 | ODD | ODDG1 | High | Low | NA | NA | 14.3 |
| PRB04699 | ODD | ODDG1 | High | Low | NA | NA | 18.6 |
| PRB05129 | ODD | ODDG1 | High | Low | Young | Female | NA |
| PRB05131 | ODD | ODDG1 | High | Low | Adult | Male | NA |
| PRB05133 | ODD | ODDG1 | High | Low | Adult | Female | NA |
| PRB05135 | ODD | ODDG1 | High | Low | Adult | Male | NA |
| PRB05137 | ODD | ODDG1 | High | Low | Adult | Male | NA |
| PRB05139 | ODD | ODDG1 | High | Low | Adult | Female | NA |
| PRB05141 | ODD | ODDG1 | High | Low | Adult | Male | NA |
| PRB05143 | ODD | ODDG1 | High | Low | NA | NA | NA |
| PRB05145 | ODD | ODDG1 | High | Low | Adult | Male | NA |
| PRB05147 | ODD | ODDG1 | High | Low | Adult | Male | NA |
| PRB05149 | ODD | ODDG1 | High | Low | NA | NA | NA |
| PRB05151 | ODD | ODDG1 | High | Low | Adult | Female | NA |
| PRB05153 | ODD | ODDG1 | High | Low | Young | NA | NA |
| PRB05155 | ODD | ODDG1 | High | Low | Adult | Female | NA |
| PRB05157 | ODD | ODDG1 | High | Low | NA | NA | NA |
| PRB05159 | ODD | ODDG1 | High | Low | Adult | Male | NA |
| PRB05161 | ODD | ODDG1 | High | Low | Adult | Male | NA |
| PRB05163 | ODD | ODDG1 | High | Low | NA | NA | NA |
| PRB05165 | ODD | ODDG1 | High | Low | NA | NA | NA |
| PRB05167 | ODD | ODDG1 | High | Low | NA | NA | NA |
| PRB05169 | ODD | ODDG1 | High | Low | NA | NA | NA |
| PRB05171 | ODD | ODDG1 | High | Low | Young | Male | NA |
| PRB05173 | ODD | ODDG1 | High | Low | NA | NA | NA |
| PRB05175 | ODD | ODDG1 | High | Low | Adult | Male | NA |
| PRB05177 | ODD | ODDG1 | High | Low | Adult | Female | NA |
| PRB05179 | ODD | ODDG1 | High | Low | NA | NA | NA |
| PRB05181 | ODD | ODDG1 | High | Low | NA | NA | NA |
| PRB05183 | ODD | ODDG1 | High | Low | NA | NA | NA |
| PRB05185 | ODD | ODDG1 | High | Low | Young | Male | NA |
| PRB05187 | ODD | ODDG1 | High | Low | Adult | Female | NA |
| PRB05189 | ODD | ODDG1 | High | Low | NA | NA | NA |
| PRB05191 | ODD | ODDG1 | High | Low | NA | NA | NA |
| PRB05193 | ODD | ODDG1 | High | Low | NA | NA | NA |
| PRB05195 | ODD | ODDG1 | High | Low | NA | NA | NA |
| PRB05197 | ODD | ODDG1 | High | Low | NA | NA | NA |
| PRB05199 | ODD | ODDG1 | High | Low | Adult | Female | NA |
| PRB05201 | ODD | ODDG1 | High | Low | NA | NA | NA |
| PRB05203 | ODD | ODDG1 | High | Low | NA | NA | NA |
| PRB05205 | ODD | ODDG1 | High | Low | NA | NA | NA |
| PRB05207 | ODD | ODDG1 | High | Low | NA | NA | NA |
| PRB05209 | ODD | ODDG1 | High | Low | Adult | Male | NA |
| PRB05211 | ODD | ODDG1 | High | Low | Adult | Male | NA |
| PRB05213 | ODD | ODDG1 | High | Low | NA | NA | NA |
| PRB05215 | ODD | ODDG1 | High | Low | NA | NA | NA |
| PRB05217 | ODD | ODDG1 | High | Low | NA | NA | NA |
| PRB05219 | ODD | ODDG1 | High | Low | Adult | Male | NA |
| PRB05221 | ODD | ODDG1 | High | Low | NA | NA | NA |
| PRB04701 | ODD | ODDG2 | High | Low | NA | Male | 14.2 |
| PRB04703 | ODD | ODDG2 | High | Low | NA | NA | 14.5 |
| PRB04705 | ODD | ODDG2 | High | Low | NA | NA | 8.5 |
| PRB04707 | ODD | ODDG2 | High | Low | NA | NA | 128.2 |
| PRB04709 | ODD | ODDG2 | High | Low | NA | NA | 6.9 |
| PRB04711 | ODD | ODDG2 | High | Low | NA | NA | 3.4 |
| PRB04713 | ODD | ODDG2 | High | Low | Adult | Female | 4.7 |
| PRB04715 | ODD | ODDG2 | High | Low | Adult | Female | 12.6 |
| PRB04717 | ODD | ODDG2 | High | Low | Adult | Male | 17.5 |
| PRB04719 | ODD | ODDG2 | High | Low | Adult | Male | 16 |
| PRB04721 | ODD | ODDG2 | High | Low | Adult | Male | 25.7 |
| PRB04723 | ODD | ODDG2 | High | Low | Adult | Female | 16 |
| PRB04725 | ODD | ODDG2 | High | Low | Young | Female | 29.4 |
| PRB04727 | ODD | ODDG2 | High | Low | NA | NA | 32.7 |
| PRB04729 | ODD | ODDG2 | High | Low | Adult | NA | 30.6 |
| PRB04731 | ODD | ODDG2 | High | Low | Adult | NA | 8.1 |
| PRB04733 | ODD | ODDG2 | High | Low | Adult | NA | 6.6 |
| PRB04735 | ODD | ODDG2 | High | Low | NA | NA | 18.6 |
| PRB04737 | ODD | ODDG2 | High | Low | Adult | NA | 14.7 |
| PRB04739 | ODD | ODDG2 | High | Low | Adult | NA | 13.8 |
| PRB04741 | ODD | ODDG2 | High | Low | NA | NA | 18 |
| PRB04743 | ODD | ODDG2 | High | Low | Adult | NA | 10.3 |
| PRB04745 | ODD | ODDG2 | High | Low | Adult | NA | 59.4 |
| PRB04747 | ODD | ODDG2 | High | Low | Adult | NA | 5.8 |
| PRB04749 | ODD | ODDG2 | High | Low | Adult | NA | 11.8 |
| PRB04751 | ODD | ODDG2 | High | Low | Adult | Female | 9.1 |
| PRB04753 | ODD | ODDG2 | High | Low | NA | NA | 2.2 |
| PRB04755 | ODD | ODDG2 | High | Low | NA | NA | 30.3 |
| PRB04757 | ODD | ODDG2 | High | Low | NA | NA | 4.4 |
| PRB04759 | ODD | ODDG2 | High | Low | Adult | Female | NA |
| PRB04761 | ODD | ODDG2 | High | Low | Adult | Male | 36.4 |
| PRB04763 | ODD | ODDG2 | High | Low | NA | NA | NA |
| PRB04765 | ODD | ODDG2 | High | Low | NA | NA | 30.4 |
| PRB04767 | ODD | ODDG2 | High | Low | NA | NA | 10.2 |
| PRB04769 | ODD | ODDG2 | High | Low | NA | NA | 43.8 |
| PRB04771 | ODD | ODDG2 | High | Low | NA | NA | 12.4 |
| PRB04773 | ODD | ODDG2 | High | Low | NA | NA | 9.7 |
| PRB04775 | ODD | ODDG2 | High | Low | NA | NA | NA |
| PRB04777 | ODD | ODDG2 | High | Low | NA | NA | 16 |
| PRB04779 | ODD | ODDG2 | High | Low | NA | NA | NA |
| PRB04781 | ODD | ODDG2 | High | Low | NA | NA | 11.8 |
| PRB04783 | ODD | ODDG2 | High | Low | NA | NA | 19.6 |
| PRB04785 | ODD | ODDG2 | High | Low | Adult | NA | 7.1 |
| PRB05045 | ODD | ODDG2 | High | Low | NA | NA | NA |
| PRB05047 | ODD | ODDG2 | High | Low | NA | NA | NA |
| PRB05049 | ODD | ODDG2 | High | Low | NA | NA | NA |
| PRB05051 | ODD | ODDG2 | High | Low | NA | NA | NA |
| PRB05053 | ODD | ODDG2 | High | Low | NA | NA | NA |
| PRB05055 | ODD | ODDG2 | High | Low | Adult | NA | NA |
| PRB05057 | ODD | ODDG2 | High | Low | NA | NA | NA |
| PRB05059 | ODD | ODDG2 | High | Low | NA | NA | NA |
| PRB05061 | ODD | ODDG2 | High | Low | NA | NA | NA |
| PRB05063 | ODD | ODDG2 | High | Low | NA | NA | NA |
| PRB05065 | ODD | ODDG2 | High | Low | NA | NA | NA |
| PRB05083 | ODD | ODDG2 | High | Low | Adult | Male | NA |
| PRB05085 | ODD | ODDG2 | High | Low | NA | NA | NA |
| PRB05087 | ODD | ODDG2 | High | Low | NA | NA | NA |
| PRB05089 | ODD | ODDG2 | High | Low | NA | NA | NA |
| PRB05091 | ODD | ODDG2 | High | Low | NA | NA | NA |
| PRB05093 | ODD | ODDG2 | High | Low | NA | NA | NA |
| PRB05095 | ODD | ODDG2 | High | Low | NA | NA | NA |
| PRB05097 | ODD | ODDG2 | High | Low | NA | NA | NA |
| PRB05099 | ODD | ODDG2 | High | Low | NA | NA | NA |
| PRB05101 | ODD | ODDG2 | High | Low | NA | NA | NA |
| PRB05103 | ODD | ODDG2 | High | Low | NA | NA | NA |
| PRB05105 | ODD | ODDG2 | High | Low | NA | NA | NA |
| PRB05107 | ODD | ODDG2 | High | Low | NA | NA | NA |
| PRB05109 | ODD | ODDG2 | High | Low | NA | NA | NA |
| PRB05111 | ODD | ODDG2 | High | Low | NA | NA | NA |
| PRB05113 | ODD | ODDG2 | High | Low | NA | NA | NA |
| PRB05115 | ODD | ODDG2 | High | Low | NA | NA | NA |
| PRB05117 | ODD | ODDG2 | High | Low | NA | NA | NA |
| PRB05119 | ODD | ODDG2 | High | Low | NA | NA | NA |
| PRB05121 | ODD | ODDG2 | High | Low | NA | NA | NA |
| PRB04219 | PBC | PBCG1 | High | Medium | NA | NA | 0.5 |
| PRB04221 | PBC | PBCG1 | High | Medium | NA | NA | 0 |
| PRB04223 | PBC | PBCG1 | High | Medium | Adult | Female | 0 |
| PRB04225 | PBC | PBCG1 | High | Medium | Adult | Female | 0 |
| PRB04227 | PBC | PBCG1 | High | Medium | Adult | Female | 1.9 |
| PRB04231 | PBC | PBCG1 | High | Medium | Adult | Male | 0 |
| PRB04233 | PBC | PBCG1 | High | Medium | Adult | Female | 0 |
| PRB04235 | PBC | PBCG1 | High | Medium | Adult | Female | 0.3 |
| PRB04237 | PBC | PBCG1 | High | Medium | Young | NA | 0 |
| PRB04239 | PBC | PBCG1 | High | Medium | Adult | NA | 0.7 |
| PRB04241 | PBC | PBCG1 | High | Medium | Adult | NA | 0.3 |
| PRB04243 | PBC | PBCG1 | High | Medium | Adult | NA | 2.1 |
| PRB04245 | PBC | PBCG1 | High | Medium | Adult | NA | 0 |
| PRB04247 | PBC | PBCG1 | High | Medium | Adult | NA | 0 |
| PRB04249 | PBC | PBCG1 | High | Medium | Adult | NA | 0.6 |
| PRB04251 | PBC | PBCG1 | High | Medium | Adult | NA | 39.9 |
| PRB04253 | PBC | PBCG1 | High | Medium | Adult | NA | 2.7 |
| PRB04255 | PBC | PBCG1 | High | Medium | NA | NA | 13.6 |
| PRB04257 | PBC | PBCG1 | High | Medium | NA | NA | 4 |
| PRB04259 | PBC | PBCG1 | High | Medium | NA | NA | 24.4 |
| PRB04261 | PBC | PBCG1 | High | Medium | NA | NA | 7.4 |
| PRB04263 | PBC | PBCG1 | High | Medium | Adult | NA | 6.8 |
| PRB04267 | PBC | PBCG1 | High | Medium | Adult | NA | 0.4 |
| PRB10509 | PBC | PBCG1 | High | Medium | Adult | Female | NA |
| PRB10511 | PBC | PBCG1 | High | Medium | Adult | Male | NA |
| PRB10513 | PBC | PBCG1 | High | Medium | Adult | Male | NA |
| PRB10515 | PBC | PBCG1 | High | Medium | Adult | Female | NA |
| PRB10517 | PBC | PBCG1 | High | Medium | Young | Female | NA |
| PRB10519 | PBC | PBCG1 | High | Medium | Adult | Male | NA |
| PRB10521 | PBC | PBCG1 | High | Medium | Adult | Female | NA |
| PRB10523 | PBC | PBCG1 | High | Medium | Young | Female | NA |
| PRB10525 | PBC | PBCG1 | High | Medium | Adult | Female | NA |
| PRB10527 | PBC | PBCG1 | High | Medium | Adult | Female | NA |
| PRB10529 | PBC | PBCG1 | High | Medium | Adult | Male | NA |
| PRB10531 | PBC | PBCG1 | High | Medium | Adult | Female | NA |
| PRB10533 | PBC | PBCG1 | High | Medium | Adult | Female | NA |
| PRB10535 | PBC | PBCG1 | High | Medium | Adult | Female | NA |
| PRB10537 | PBC | PBCG1 | High | Medium | Adult | Male | NA |
| PRB10539 | PBC | PBCG1 | High | Medium | Adult | Female | NA |
| PRB10541 | PBC | PBCG1 | High | Medium | Adult | Male | NA |
| PRB10543 | PBC | PBCG1 | High | Medium | Adult | Female | NA |
| PRB10545 | PBC | PBCG1 | High | Medium | Young | Male | NA |
| PRB10547 | PBC | PBCG1 | High | Medium | Adult | Male | NA |
| PRB10549 | PBC | PBCG1 | High | Medium | Adult | Male | NA |
| PRB10551 | PBC | PBCG1 | High | Medium | Adult | Female | NA |
| PRB10553 | PBC | PBCG1 | High | Medium | Young | Female | NA |
| PRB10555 | PBC | PBCG1 | High | Medium | Young | Male | NA |
| PRB10557 | PBC | PBCG1 | High | Medium | Adult | Female | NA |
| PRB10559 | PBC | PBCG1 | High | Medium | Adult | Male | NA |
| PRB10561 | PBC | PBCG1 | High | Medium | Adult | Female | NA |
| PRB10563 | PBC | PBCG1 | High | Medium | Young | Female | NA |
| PRB10565 | PBC | PBCG1 | High | Medium | Adult | Male | NA |
| PRB04269 | PBC | PBCG2 | High | Medium | NA | NA | 6.6 |
| PRB04271 | PBC | PBCG2 | High | Medium | Young | NA | 2.4 |
| PRB04273 | PBC | PBCG2 | High | Medium | NA | NA | 6.4 |
| PRB04275 | PBC | PBCG2 | High | Medium | Adult | NA | 5.8 |
| PRB04277 | PBC | PBCG2 | High | Medium | NA | NA | 10.4 |
| PRB04279 | PBC | PBCG2 | High | Medium | Adult | NA | 6.5 |
| PRB04281 | PBC | PBCG2 | High | Medium | Adult | NA | 2.7 |
| PRB04283 | PBC | PBCG2 | High | Medium | Adult | NA | 0.9 |
| PRB04285 | PBC | PBCG2 | High | Medium | Young | NA | 15.6 |
| PRB04287 | PBC | PBCG2 | High | Medium | Adult | NA | NA |
| PRB10569 | PBC | PBCG2 | High | Medium | Adult | Male | NA |
| PRB10571 | PBC | PBCG2 | High | Medium | Adult | Female | NA |
| PRB10573 | PBC | PBCG2 | High | Medium | Adult | Female | NA |
| PRB10575 | PBC | PBCG2 | High | Medium | Young | Male | NA |
| PRB10577 | PBC | PBCG2 | High | Medium | Adult | Female | NA |
| PRB10579 | PBC | PBCG2 | High | Medium | Adult | Male | NA |
| PRB10581 | PBC | PBCG2 | High | Medium | Adult | Female | NA |
| PRB10583 | PBC | PBCG2 | High | Medium | Young | Female | NA |
| PRB10585 | PBC | PBCG2 | High | Medium | Young | Male | NA |
| PRB10587 | PBC | PBCG2 | High | Medium | Young | Female | NA |
| PRB10589 | PBC | PBCG2 | High | Medium | Adult | Female | NA |
| PRB10591 | PBC | PBCG2 | High | Medium | Adult | Female | NA |
| PRB10593 | PBC | PBCG2 | High | Medium | Young | Female | NA |
| PRB10595 | PBC | PBCG2 | High | Medium | Adult | Female | NA |
| PRB10597 | PBC | PBCG2 | High | Medium | Adult | Female | NA |
| PRB10599 | PBC | PBCG2 | High | Medium | Adult | Male | NA |
| PRB10601 | PBC | PBCG2 | High | Medium | Adult | Male | NA |
| PRB10603 | PBC | PBCG2 | High | Medium | Adult | Female | NA |
| PRB10605 | PBC | PBCG2 | High | Medium | Young | Male | NA |
| PRB10607 | PBC | PBCG2 | High | Medium | Adult | Female | NA |
| PRB10609 | PBC | PBCG2 | High | Medium | Adult | Female | NA |
| PRB10611 | PBC | PBCG2 | High | Medium | Adult | Female | NA |
| PRB10613 | PBC | PBCG2 | High | Medium | Adult | Female | NA |
| PRB10615 | PBC | PBCG2 | High | Medium | Young | Female | NA |
| PRB10617 | PBC | PBCG2 | High | Medium | Young | Male | NA |
| PRB10619 | PBC | PBCG2 | High | Medium | Adult | Female | NA |
| PRB10621 | PBC | PBCG2 | High | Medium | Adult | Female | NA |
| PRB10623 | PBC | PBCG2 | High | Medium | Adult | Male | NA |
| PRB10625 | PBC | PBCG2 | High | Medium | Adult | Female | NA |
| PRB10627 | PBC | PBCG2 | High | Medium | Adult | Male | NA |
| PRB10629 | PBC | PBCG2 | High | Medium | Adult | Female | NA |
| PRB10631 | PBC | PBCG2 | High | Medium | Adult | Male | NA |
| PRB10633 | PBC | PBCG2 | High | Medium | Adult | Female | NA |
| PRB10635 | PBC | PBCG2 | High | Medium | Adult | Female | NA |
| PRB10637 | PBC | PBCG2 | High | Medium | Adult | Female | NA |
| PRB10639 | PBC | PBCG2 | High | Medium | Adult | Male | NA |
| PRB10641 | PBC | PBCG2 | High | Medium | Adult | Female | NA |
| PRB10643 | PBC | PBCG2 | High | Medium | Adult | Female | NA |
| PRB10649 | PBC | PBCG3 | High | Medium | Adult | Male | NA |
| PRB10651 | PBC | PBCG3 | High | Medium | Adult | Female | NA |
| PRB10653 | PBC | PBCG3 | High | Medium | Adult | Female | NA |
| PRB10655 | PBC | PBCG3 | High | Medium | Adult | Male | NA |
| PRB10657 | PBC | PBCG3 | High | Medium | Young | Male | NA |
| PRB10659 | PBC | PBCG3 | High | Medium | Adult | Female | NA |
| PRB10661 | PBC | PBCG3 | High | Medium | Adult | Female | NA |
| PRB10663 | PBC | PBCG3 | High | Medium | Adult | Male | NA |
| PRB10665 | PBC | PBCG3 | High | Medium | Adult | Female | NA |
| PRB10667 | PBC | PBCG3 | High | Medium | Adult | Female | NA |
| PRB10669 | PBC | PBCG3 | High | Medium | Young | Male | NA |
| PRB10671 | PBC | PBCG3 | High | Medium | Young | Male | NA |
| PRB10673 | PBC | PBCG3 | High | Medium | Adult | Female | NA |
| PRB10675 | PBC | PBCG3 | High | Medium | Adult | Female | NA |
| PRB10677 | PBC | PBCG3 | High | Medium | Adult | Male | NA |
| PRB10679 | PBC | PBCG3 | High | Medium | Adult | Female | NA |
| PRB10681 | PBC | PBCG3 | High | Medium | Young | Female | NA |
| PRB10683 | PBC | PBCG3 | High | Medium | Adult | Female | NA |
| PRB10689 | PBC | PBCG3 | High | Medium | Young | Female | NA |
| PRB10691 | PBC | PBCG3 | High | Medium | Young | Male | NA |
| PRB10693 | PBC | PBCG3 | High | Medium | Adult | Male | NA |
| PRB10695 | PBC | PBCG3 | High | Medium | Adult | Female | NA |
| PRB10697 | PBC | PBCG3 | High | Medium | Young | Male | NA |
| PRB10699 | PBC | PBCG3 | High | Medium | Young | Female | NA |
| PRB10701 | PBC | PBCG3 | High | Medium | Young | Male | NA |
| PRB06815 | PKG | PKGG1 | Medium | High | NA | NA | NA |
| PRB06817 | PKG | PKGG1 | Medium | High | NA | NA | NA |
| PRB06819 | PKG | PKGG1 | Medium | High | NA | NA | NA |
| PRB06821 | PKG | PKGG1 | Medium | High | NA | NA | NA |
| PRB06823 | PKG | PKGG1 | Medium | High | NA | NA | NA |
| PRB06825 | PKG | PKGG1 | Medium | High | NA | NA | NA |
| PRB06827 | PKG | PKGG1 | Medium | High | NA | NA | NA |
| PRB06829 | PKG | PKGG1 | Medium | High | Adult | Female | NA |
| PRB06831 | PKG | PKGG1 | Medium | High | Adult | Female | NA |
| PRB06833 | PKG | PKGG1 | Medium | High | Young | Male | NA |
| PRB06835 | PKG | PKGG1 | Medium | High | NA | NA | NA |
| PRB06837 | PKG | PKGG1 | Medium | High | NA | NA | NA |
| PRB06839 | PKG | PKGG1 | Medium | High | NA | NA | NA |
| PRB06841 | PKG | PKGG1 | Medium | High | NA | NA | NA |
| PRB06843 | PKG | PKGG1 | Medium | High | NA | NA | NA |
| PRB06845 | PKG | PKGG1 | Medium | High | NA | NA | NA |
| PRB06847 | PKG | PKGG1 | Medium | High | NA | NA | NA |
| PRB06849 | PKG | PKGG1 | Medium | High | NA | NA | NA |
| PRB06851 | PKG | PKGG1 | Medium | High | NA | NA | NA |
| PRB06853 | PKG | PKGG1 | Medium | High | NA | NA | NA |
| PRB06855 | PKG | PKGG1 | Medium | High | NA | NA | NA |
| PRB06857 | PKG | PKGG1 | Medium | High | NA | NA | NA |
| PRB06859 | PKG | PKGG1 | Medium | High | NA | NA | NA |
| PRB06861 | PKG | PKGG1 | Medium | High | NA | NA | NA |
| PRB06863 | PKG | PKGG1 | Medium | High | NA | NA | NA |
| PRB06865 | PKG | PKGG1 | Medium | High | NA | NA | NA |
| PRB06867 | PKG | PKGG1 | Medium | High | NA | NA | NA |
| PRB06869 | PKG | PKGG1 | Medium | High | NA | NA | NA |
| PRB06871 | PKG | PKGG1 | Medium | High | NA | NA | NA |
| PRB06873 | PKG | PKGG1 | Medium | High | NA | NA | NA |
| PRB06875 | PKG | PKGG1 | Medium | High | NA | NA | NA |
| PRB06877 | PKG | PKGG1 | Medium | High | NA | NA | NA |
| PRB06879 | PKG | PKGG1 | Medium | High | Adult | Male | NA |
| PRB06881 | PKG | PKGG1 | Medium | High | NA | NA | NA |
| PRB06883 | PKG | PKGG1 | Medium | High | NA | NA | NA |
| PRB06885 | PKG | PKGG1 | Medium | High | NA | NA | NA |
| PRB06887 | PKG | PKGG1 | Medium | High | NA | NA | NA |
| PRB06889 | PKG | PKGG1 | Medium | High | NA | NA | NA |
| PRB06891 | PKG | PKGG1 | Medium | High | NA | NA | NA |
| PRB06893 | PKG | PKGG1 | Medium | High | NA | NA | NA |
| PRB06895 | PKG | PKGG1 | Medium | High | NA | NA | NA |
| PRB06897 | PKG | PKGG1 | Medium | High | NA | NA | NA |
| PRB06899 | PKG | PKGG1 | Medium | High | NA | NA | NA |
| PRB06901 | PKG | PKGG1 | Medium | High | NA | NA | NA |
| PRB06903 | PKG | PKGG1 | Medium | High | NA | NA | NA |
| PRB06905 | PKG | PKGG1 | Medium | High | NA | NA | NA |
| PRB06907 | PKG | PKGG1 | Medium | High | Adult | Male | NA |
| PRB06909 | PKG | PKGG1 | Medium | High | NA | NA | NA |
| PRB06911 | PKG | PKGG1 | Medium | High | NA | NA | NA |
| PRB06913 | PKG | PKGG1 | Medium | High | NA | NA | NA |
| PRB06915 | PKG | PKGG1 | Medium | High | NA | NA | NA |
| PRB06917 | PKG | PKGG1 | Medium | High | NA | NA | NA |
| PRB06919 | PKG | PKGG1 | Medium | High | NA | NA | NA |
| PRB06921 | PKG | PKGG1 | Medium | High | NA | NA | NA |
| PRB06923 | PKG | PKGG1 | Medium | High | Adult | Male | NA |
| PRB06925 | PKG | PKGG1 | Medium | High | NA | NA | NA |
| PRB06927 | PKG | PKGG1 | Medium | High | NA | NA | NA |
| PRB06929 | PKG | PKGG1 | Medium | High | NA | NA | NA |
| PRB06931 | PKG | PKGG1 | Medium | High | NA | NA | NA |
| PRB06933 | PKG | PKGG1 | Medium | High | NA | NA | NA |
| PRB06935 | PKG | PKGG1 | Medium | High | NA | NA | NA |
| PRB06937 | PKG | PKGG1 | Medium | High | NA | NA | NA |
| PRB06939 | PKG | PKGG1 | Medium | High | NA | NA | NA |
| PRB06941 | PKG | PKGG1 | Medium | High | NA | NA | NA |
| PRB06943 | PKG | PKGG1 | Medium | High | NA | NA | NA |
| PRB06945 | PKG | PKGG1 | Medium | High | NA | NA | NA |
| PRB06947 | PKG | PKGG1 | Medium | High | NA | NA | NA |
| PRB06949 | PKG | PKGG1 | Medium | High | NA | NA | NA |
| PRB06951 | PKG | PKGG1 | Medium | High | NA | NA | NA |
| PRB06953 | PKG | PKGG1 | Medium | High | NA | NA | NA |
| PRB06955 | PKG | PKGG1 | Medium | High | NA | NA | NA |
| PRB06957 | PKG | PKGG1 | Medium | High | NA | NA | NA |
| PRB06959 | PKG | PKGG1 | Medium | High | NA | NA | NA |
| PRB09973 | RBMR | RBMRG1 | Low | Medium | Adult | Male | NA |
| PRB09975 | RBMR | RBMRG1 | Low | Medium | Adult | Female | NA |
| PRB09977 | RBMR | RBMRG1 | Low | Medium | Adult | Female | NA |
| PRB09979 | RBMR | RBMRG1 | Low | Medium | Adult | Male | NA |
| PRB09981 | RBMR | RBMRG1 | Low | Medium | Young | Female | NA |
| PRB09983 | RBMR | RBMRG1 | Low | Medium | Adult | Female | NA |
| PRB09985 | RBMR | RBMRG1 | Low | Medium | Young | Female | NA |
| PRB09987 | RBMR | RBMRG1 | Low | Medium | Adult | Female | NA |
| PRB09989 | RBMR | RBMRG1 | Low | Medium | Young | Male | NA |
| PRB09991 | RBMR | RBMRG1 | Low | Medium | Young | Female | NA |
| PRB09993 | RBMR | RBMRG1 | Low | Medium | Adult | Female | NA |
| PRB09995 | RBMR | RBMRG1 | Low | Medium | Young | Male | NA |
| PRB09997 | RBMR | RBMRG1 | Low | Medium | Young | Female | NA |
| PRB09999 | RBMR | RBMRG1 | Low | Medium | Adult | Female | NA |
| PRB10001 | RBMR | RBMRG1 | Low | Medium | Adult | Male | NA |
| PRB10003 | RBMR | RBMRG1 | Low | Medium | Adult | Female | NA |
| PRB10005 | RBMR | RBMRG1 | Low | Medium | Young | Female | NA |
| PRB10007 | RBMR | RBMRG1 | Low | Medium | Adult | Male | NA |
| PRB10009 | RBMR | RBMRG1 | Low | Medium | Adult | Female | NA |
| PRB10011 | RBMR | RBMRG1 | Low | Medium | Young | Male | NA |
| PRB10013 | RBMR | RBMRG1 | Low | Medium | Young | Male | NA |
| PRB10015 | RBMR | RBMRG1 | Low | Medium | Adult | Female | NA |
| PRB10017 | RBMR | RBMRG1 | Low | Medium | Adult | Male | NA |
| PRB10019 | RBMR | RBMRG1 | Low | Medium | Adult | Male | NA |
| PRB10021 | RBMR | RBMRG1 | Low | Medium | Adult | Female | NA |
| PRB10023 | RBMR | RBMRG1 | Low | Medium | Adult | Female | NA |
| PRB10025 | RBMR | RBMRG1 | Low | Medium | Young | Male | NA |
| PRB10027 | RBMR | RBMRG1 | Low | Medium | Adult | Male | NA |
| PRB10029 | RBMR | RBMRG1 | Low | Medium | Adult | Male | NA |
| PRB10031 | RBMR | RBMRG1 | Low | Medium | Adult | Male | NA |
| PRB10033 | RBMR | RBMRG1 | Low | Medium | Adult | Male | NA |
| PRB10035 | RBMR | RBMRG1 | Low | Medium | Adult | Male | NA |
| PRB10037 | RBMR | RBMRG1 | Low | Medium | Young | Female | NA |
| PRB10039 | RBMR | RBMRG1 | Low | Medium | Adult | Female | NA |
| PRB10041 | RBMR | RBMRG1 | Low | Medium | Adult | Male | NA |
| PRB10043 | RBMR | RBMRG1 | Low | Medium | Adult | Female | NA |
| PRB10045 | RBMR | RBMRG1 | Low | Medium | Adult | Female | NA |
| PRB10047 | RBMR | RBMRG1 | Low | Medium | Young | Female | NA |
| PRB10049 | RBMR | RBMRG1 | Low | Medium | Young | Male | NA |
| PRB10051 | RBMR | RBMRG1 | Low | Medium | Young | Female | NA |
| PRB10053 | RBMR | RBMRG1 | Low | Medium | Adult | Female | NA |
| PRB10055 | RBMR | RBMRG1 | Low | Medium | Young | Male | NA |
| PRB10057 | RBMR | RBMRG1 | Low | Medium | Adult | Female | NA |
| PRB10059 | RBMR | RBMRG1 | Low | Medium | Adult | Female | NA |
| PRB10061 | RBMR | RBMRG1 | Low | Medium | Adult | Male | NA |
| PRB10063 | RBMR | RBMRG1 | Low | Medium | Adult | Male | NA |
| PRB10065 | RBMR | RBMRG1 | Low | Medium | Adult | Female | NA |
| PRB10067 | RBMR | RBMRG1 | Low | Medium | Young | Female | NA |
| PRB10069 | RBMR | RBMRG1 | Low | Medium | Young | Male | NA |
| PRB10071 | RBMR | RBMRG1 | Low | Medium | Adult | Female | NA |
| PRB10073 | RBMR | RBMRG1 | Low | Medium | Adult | Female | NA |
| PRB10075 | RBMR | RBMRG1 | Low | Medium | Young | Male | NA |
| PRB10077 | RBMR | RBMRG1 | Low | Medium | Adult | Female | NA |
| PRB10079 | RBMR | RBMRG1 | Low | Medium | Adult | Female | NA |
| PRB10081 | RBMR | RBMRG1 | Low | Medium | Adult | Male | NA |
| PRB10083 | RBMR | RBMRG1 | Low | Medium | Adult | Female | NA |
| PRB10085 | RBMR | RBMRG1 | Low | Medium | Adult | Male | NA |
| PRB10087 | RBMR | RBMRG1 | Low | Medium | Adult | Female | NA |
| PRB10089 | RBMR | RBMRG1 | Low | Medium | Young | Female | NA |
| PRB10091 | RBMR | RBMRG1 | Low | Medium | Adult | Female | NA |
| PRB10093 | RBMR | RBMRG1 | Low | Medium | Adult | Female | NA |
| PRB10095 | RBMR | RBMRG1 | Low | Medium | Young | Male | NA |
| PRB10097 | RBMR | RBMRG1 | Low | Medium | Adult | Female | NA |
| PRB10099 | RBMR | RBMRG1 | Low | Medium | Adult | Female | NA |
| PRB10101 | RBMR | RBMRG1 | Low | Medium | Adult | Male | NA |
| PRB10103 | RBMR | RBMRG1 | Low | Medium | Young | Male | NA |
| PRB10105 | RBMR | RBMRG1 | Low | Medium | Young | Female | NA |
| PRB10107 | RBMR | RBMRG1 | Low | Medium | Adult | Male | NA |
| PRB10109 | RBMR | RBMRG1 | Low | Medium | Adult | Female | NA |
| PRB10111 | RBMR | RBMRG1 | Low | Medium | Adult | Male | NA |
| PRB10113 | RBMR | RBMRG1 | Low | Medium | Adult | Female | NA |
| PRB10115 | RBMR | RBMRG1 | Low | Medium | Adult | Male | NA |
| PRB10117 | RBMR | RBMRG1 | Low | Medium | Adult | Female | NA |
| PRB10119 | RBMR | RBMRG1 | Low | Medium | Adult | Female | NA |
| PRB10121 | RBMR | RBMRG1 | Low | Medium | Adult | Male | NA |
| PRB10123 | RBMR | RBMRG1 | Low | Medium | Adult | Male | NA |
| PRB10125 | RBMR | RBMRG1 | Low | Medium | Young | Female | NA |
| PRB10127 | RBMR | RBMRG1 | Low | Medium | Young | Male | NA |
| PRB10129 | RBMR | RBMRG1 | Low | Medium | Young | Male | NA |
| PRB10131 | RBMR | RBMRG1 | Low | Medium | Young | Female | NA |
| PRB10133 | RBMR | RBMRG1 | Low | Medium | Adult | Female | NA |
| PRB10135 | RBMR | RBMRG1 | Low | Medium | Young | Male | NA |
| PRB10137 | RBMR | RBMRG1 | Low | Medium | Adult | Female | NA |
| PRB02761 | RN | RNG1 | High | High | Adult | Female | 9.3 |
| PRB02765 | RN | RNG1 | High | High | Adult | Female | 18.7 |
| PRB02767 | RN | RNG1 | High | High | Adult | Female | 2.3 |
| PRB02771 | RN | RNG1 | High | High | Adult | Female | 17.8 |
| PRB02775 | RN | RNG1 | High | High | Adult | Female | 10.4 |
| PRB02777 | RN | RNG1 | High | High | Adult | Male | 39 |
| PRB02779 | RN | RNG1 | High | High | NA | NA | 10 |
| PRB02781 | RN | RNG1 | High | High | Adult | NA | 12.1 |
| PRB02783 | RN | RNG1 | High | High | Adult | Male | 5.7 |
| PRB02785 | RN | RNG1 | High | High | Young | Female | NA |
| PRB02787 | RN | RNG1 | High | High | Young | Male | NA |
| PRB02789 | RN | RNG1 | High | High | Adult | Female | NA |
| PRB02791 | RN | RNG1 | High | High | Adult | Female | 6.7 |
| PRB02793 | RN | RNG1 | High | High | Adult | Female | 14.6 |
| PRB02795 | RN | RNG1 | High | High | NA | NA | 28.2 |
| PRB02797 | RN | RNG1 | High | High | NA | NA | 19.7 |
| PRB02799 | RN | RNG1 | High | High | NA | NA | 10.3 |
| PRB02801 | RN | RNG1 | High | High | NA | NA | 14.5 |
| PRB02803 | RN | RNG1 | High | High | NA | NA | 10 |
| PRB02805 | RN | RNG1 | High | High | NA | NA | 32.4 |
| PRB02807 | RN | RNG1 | High | High | Adult | Female | 14.8 |
| PRB02809 | RN | RNG1 | High | High | Adult | Female | 15.2 |
| PRB02811 | RN | RNG1 | High | High | Adult | Female | 11.1 |
| PRB02813 | RN | RNG1 | High | High | NA | NA | 10.3 |
| PRB08741 | RN | RNG1 | High | High | Adult | Female | NA |
| PRB08743 | RN | RNG1 | High | High | NA | NA | NA |
| PRB08745 | RN | RNG1 | High | High | Adult | Male | NA |
| PRB08747 | RN | RNG1 | High | High | NA | NA | NA |
| PRB08749 | RN | RNG1 | High | High | NA | NA | NA |
| PRB08751 | RN | RNG1 | High | High | Young | Female | NA |
| PRB08753 | RN | RNG1 | High | High | Adult | Female | NA |
| PRB08755 | RN | RNG1 | High | High | NA | NA | NA |
| PRB08757 | RN | RNG1 | High | High | NA | NA | NA |
| PRB08759 | RN | RNG1 | High | High | NA | NA | NA |
| PRB08761 | RN | RNG1 | High | High | Adult | Female | NA |
| PRB08763 | RN | RNG1 | High | High | NA | NA | NA |
| PRB08765 | RN | RNG1 | High | High | NA | NA | NA |
| PRB08767 | RN | RNG1 | High | High | NA | NA | NA |
| PRB08769 | RN | RNG1 | High | High | NA | NA | NA |
| PRB08771 | RN | RNG1 | High | High | NA | NA | NA |
| PRB08773 | RN | RNG1 | High | High | Adult | Female | NA |
| PRB08775 | RN | RNG1 | High | High | NA | NA | NA |
| PRB08777 | RN | RNG1 | High | High | NA | NA | NA |
| PRB08779 | RN | RNG1 | High | High | NA | NA | NA |
| PRB08781 | RN | RNG1 | High | High | NA | NA | NA |
| PRB08783 | RN | RNG1 | High | High | NA | NA | NA |
| PRB08785 | RN | RNG1 | High | High | NA | NA | NA |
| PRB08787 | RN | RNG1 | High | High | NA | NA | NA |
| PRB08789 | RN | RNG1 | High | High | NA | NA | NA |
| PRB08791 | RN | RNG1 | High | High | NA | NA | NA |
| PRB08793 | RN | RNG1 | High | High | NA | NA | NA |
| PRB08795 | RN | RNG1 | High | High | NA | NA | NA |
| PRB08797 | RN | RNG1 | High | High | NA | NA | NA |
| PRB08799 | RN | RNG1 | High | High | NA | NA | NA |
| PRB08801 | RN | RNG1 | High | High | NA | NA | NA |
| PRB08803 | RN | RNG1 | High | High | NA | NA | NA |
| PRB08805 | RN | RNG1 | High | High | NA | NA | NA |
| PRB08807 | RN | RNG1 | High | High | NA | NA | NA |
| PRB08809 | RN | RNG1 | High | High | NA | NA | NA |
| PRB08811 | RN | RNG1 | High | High | NA | NA | NA |
| PRB08813 | RN | RNG1 | High | High | NA | NA | NA |
| PRB08815 | RN | RNG1 | High | High | NA | NA | NA |
| PRB08817 | RN | RNG1 | High | High | NA | NA | NA |
| PRB08819 | RN | RNG1 | High | High | NA | NA | NA |
| PRB02815 | RN | RNG2 | High | High | Adult | Male | 20.3 |
| PRB02817 | RN | RNG2 | High | High | Adult | Female | 12.7 |
| PRB02819 | RN | RNG2 | High | High | Adult | Female | 12.4 |
| PRB02821 | RN | RNG2 | High | High | Adult | Female | 24 |
| PRB02823 | RN | RNG2 | High | High | NA | NA | 20.1 |
| PRB08869 | RN | RNG2 | High | High | NA | NA | NA |
| PRB08871 | RN | RNG2 | High | High | NA | NA | NA |
| PRB08873 | RN | RNG2 | High | High | NA | NA | NA |
| PRB08875 | RN | RNG2 | High | High | NA | NA | NA |
| PRB08877 | RN | RNG2 | High | High | NA | NA | NA |
| PRB08879 | RN | RNG2 | High | High | NA | NA | NA |
| PRB08881 | RN | RNG2 | High | High | NA | NA | NA |
| PRB08883 | RN | RNG2 | High | High | NA | NA | NA |
| PRB08885 | RN | RNG2 | High | High | NA | NA | NA |
| PRB08887 | RN | RNG2 | High | High | Adult | Male | NA |
| PRB08889 | RN | RNG2 | High | High | NA | NA | NA |
| PRB08891 | RN | RNG2 | High | High | NA | NA | NA |
| PRB08893 | RN | RNG2 | High | High | NA | NA | NA |
| PRB08895 | RN | RNG2 | High | High | NA | NA | NA |
| PRB08897 | RN | RNG2 | High | High | Adult | Female | NA |
| PRB08899 | RN | RNG2 | High | High | NA | NA | NA |
| PRB08901 | RN | RNG2 | High | High | NA | NA | NA |
| PRB08903 | RN | RNG2 | High | High | NA | Male | NA |
| PRB08905 | RN | RNG2 | High | High | NA | NA | NA |
| PRB08907 | RN | RNG2 | High | High | NA | NA | NA |
| PRB08909 | RN | RNG2 | High | High | NA | NA | NA |
| PRB08911 | RN | RNG2 | High | High | NA | NA | NA |
| PRB08913 | RN | RNG2 | High | High | NA | NA | NA |
| PRB08915 | RN | RNG2 | High | High | NA | NA | NA |
| PRB02831 | RN | RNG3 | High | High | Adult | Female | 16.1 |
| PRB02833 | RN | RNG3 | High | High | Young | NA | 22.9 |
| PRB02835 | RN | RNG3 | High | High | Adult | Female | 15 |
| PRB02837 | RN | RNG3 | High | High | Adult | NA | 21.5 |
| PRB02839 | RN | RNG3 | High | High | Adult | Female | 0 |
| PRB02841 | RN | RNG3 | High | High | Adult | Female | 1.7 |
| PRB02847 | RN | RNG3 | High | High | Adult | Male | 0.5 |
| PRB02849 | RN | RNG3 | High | High | Young | NA | 0.3 |
| PRB02851 | RN | RNG3 | High | High | Adult | Female | 0.5 |
| PRB02853 | RN | RNG3 | High | High | Adult | Male | 3.5 |
| PRB02855 | RN | RNG3 | High | High | Adult | Female | 0.3 |
| PRB02857 | RN | RNG3 | High | High | Adult | Female | 1.1 |
| PRB02859 | RN | RNG3 | High | High | Adult | Female | 0.4 |
| PRB02861 | RN | RNG3 | High | High | Adult | Female | 9.3 |
| PRB02863 | RN | RNG3 | High | High | Adult | Female | 3.6 |
| PRB02865 | RN | RNG3 | High | High | Adult | Female | 4.8 |
| PRB02867 | RN | RNG3 | High | High | Adult | Female | 14.1 |
| PRB08949 | RN | RNG3 | High | High | NA | NA | NA |
| PRB08951 | RN | RNG3 | High | High | NA | NA | NA |
| PRB08953 | RN | RNG3 | High | High | NA | NA | NA |
| PRB08955 | RN | RNG3 | High | High | NA | NA | NA |
| PRB08957 | RN | RNG3 | High | High | NA | NA | NA |
| PRB08959 | RN | RNG3 | High | High | NA | NA | NA |
| PRB08961 | RN | RNG3 | High | High | NA | NA | NA |
| PRB08963 | RN | RNG3 | High | High | NA | NA | NA |
| PRB08965 | RN | RNG3 | High | High | NA | NA | NA |
| PRB08967 | RN | RNG3 | High | High | NA | NA | NA |
| PRB08969 | RN | RNG3 | High | High | NA | NA | NA |
| PRB08971 | RN | RNG3 | High | High | NA | NA | NA |
| PRB08973 | RN | RNG3 | High | High | NA | NA | NA |
| PRB08975 | RN | RNG3 | High | High | NA | NA | NA |
| PRB08977 | RN | RNG3 | High | High | NA | NA | NA |
| PRB08979 | RN | RNG3 | High | High | NA | NA | NA |
| PRB08981 | RN | RNG3 | High | High | NA | NA | NA |
| PRB08983 | RN | RNG3 | High | High | NA | NA | NA |
| PRB08985 | RN | RNG3 | High | High | NA | NA | NA |
| PRB08987 | RN | RNG3 | High | High | NA | NA | NA |
| PRB08989 | RN | RNG3 | High | High | NA | NA | NA |
| PRB08991 | RN | RNG3 | High | High | NA | NA | NA |
| PRB08993 | RN | RNG3 | High | High | NA | NA | NA |
| PRB08995 | RN | RNG3 | High | High | NA | NA | NA |
| PRB08997 | RN | RNG3 | High | High | NA | NA | NA |
| PRB08999 | RN | RNG3 | High | High | NA | NA | NA |
| PRB09001 | RN | RNG3 | High | High | NA | NA | NA |
| PRB09003 | RN | RNG3 | High | High | NA | NA | NA |
| PRB09005 | RN | RNG3 | High | High | NA | NA | NA |
| PRB09007 | RN | RNG3 | High | High | NA | NA | NA |
| PRB09009 | RN | RNG3 | High | High | NA | NA | NA |
| PRB09011 | RN | RNG3 | High | High | NA | NA | NA |
| PRB09013 | RN | RNG3 | High | High | NA | NA | NA |
| PRB09015 | RN | RNG3 | High | High | NA | NA | NA |
| PRB09017 | RN | RNG3 | High | High | NA | NA | NA |
| PRB03943 | SJMS | SJMSG1 | Low | Low | NA | NA | 1.7 |
| PRB03945 | SJMS | SJMSG1 | Low | Low | NA | NA | 5.4 |
| PRB03947 | SJMS | SJMSG1 | Low | Low | NA | NA | 2.2 |
| PRB03949 | SJMS | SJMSG1 | Low | Low | Young | Male | 1.2 |
| PRB03951 | SJMS | SJMSG1 | Low | Low | Adult | NA | 3.1 |
| PRB03953 | SJMS | SJMSG1 | Low | Low | Adult | NA | 11.6 |
| PRB03955 | SJMS | SJMSG1 | Low | Low | Adult | Female | 9.4 |
| PRB03957 | SJMS | SJMSG1 | Low | Low | Adult | Female | 24.9 |
| PRB03959 | SJMS | SJMSG1 | Low | Low | Adult | NA | 2.9 |
| PRB03961 | SJMS | SJMSG1 | Low | Low | Adult | Male | 4.4 |
| PRB03963 | SJMS | SJMSG1 | Low | Low | NA | NA | 4.3 |
| PRB03965 | SJMS | SJMSG1 | Low | Low | NA | NA | 2.6 |
| PRB03967 | SJMS | SJMSG1 | Low | Low | Adult | Female | 3.9 |
| PRB03969 | SJMS | SJMSG1 | Low | Low | Adult | Female | 4.3 |
| PRB03971 | SJMS | SJMSG1 | Low | Low | NA | NA | 1.3 |
| PRB03973 | SJMS | SJMSG1 | Low | Low | Adult | Female | 3.3 |
| PRB03975 | SJMS | SJMSG1 | Low | Low | Adult | Female | 1.9 |
| PRB03977 | SJMS | SJMSG1 | Low | Low | Adult | NA | 0.6 |
| PRB03979 | SJMS | SJMSG1 | Low | Low | NA | NA | NA |
| PRB03981 | SJMS | SJMSG1 | Low | Low | Adult | Female | 1.2 |
| PRB03983 | SJMS | SJMSG1 | Low | Low | Adult | Female | 9.4 |
| PRB03985 | SJMS | SJMSG1 | Low | Low | Adult | Male | 0.9 |
| PRB03987 | SJMS | SJMSG1 | Low | Low | Adult | Male | NA |
| PRB03989 | SJMS | SJMSG1 | Low | Low | Adult | Male | 6.7 |
| PRB03991 | SJMS | SJMSG1 | Low | Low | NA | NA | 5.8 |
| PRB03993 | SJMS | SJMSG1 | Low | Low | NA | NA | 4.4 |
| PRB03995 | SJMS | SJMSG1 | Low | Low | Adult | Female | NA |
| PRB03997 | SJMS | SJMSG1 | Low | Low | Adult | Female | 2.6 |
| PRB03999 | SJMS | SJMSG1 | Low | Low | Adult | Male | 1 |
| PRB04001 | SJMS | SJMSG1 | Low | Low | Adult | NA | 0.5 |
| PRB04003 | SJMS | SJMSG1 | Low | Low | NA | NA | 2.8 |
| PRB04005 | SJMS | SJMSG1 | Low | Low | Adult | Male | NA |
| PRB04007 | SJMS | SJMSG1 | Low | Low | Adult | Male | 1.5 |
| PRB04009 | SJMS | SJMSG1 | Low | Low | NA | NA | 1.2 |
| PRB04011 | SJMS | SJMSG1 | Low | Low | Adult | Female | 2.3 |
| PRB04013 | SJMS | SJMSG1 | Low | Low | Adult | Male | 2.3 |
| PRB04083 | SJMS | SJMSG1 | Low | Low | NA | NA | 19.3 |
| PRB09523 | SJMS | SJMSG1 | Low | Low | NA | NA | NA |
| PRB09525 | SJMS | SJMSG1 | Low | Low | NA | NA | NA |
| PRB09527 | SJMS | SJMSG1 | Low | Low | NA | NA | NA |
| PRB09529 | SJMS | SJMSG1 | Low | Low | NA | NA | NA |
| PRB09531 | SJMS | SJMSG1 | Low | Low | NA | NA | NA |
| PRB09533 | SJMS | SJMSG1 | Low | Low | NA | NA | NA |
| PRB09535 | SJMS | SJMSG1 | Low | Low | NA | NA | NA |
| PRB09537 | SJMS | SJMSG1 | Low | Low | NA | NA | NA |
| PRB09539 | SJMS | SJMSG1 | Low | Low | NA | NA | NA |
| PRB09541 | SJMS | SJMSG1 | Low | Low | NA | NA | NA |
| PRB09543 | SJMS | SJMSG1 | Low | Low | NA | NA | NA |
| PRB09545 | SJMS | SJMSG1 | Low | Low | NA | NA | NA |
| PRB09547 | SJMS | SJMSG1 | Low | Low | Adult | Female | NA |
| PRB09549 | SJMS | SJMSG1 | Low | Low | NA | NA | NA |
| PRB09551 | SJMS | SJMSG1 | Low | Low | NA | NA | NA |
| PRB09553 | SJMS | SJMSG1 | Low | Low | NA | NA | NA |
| PRB09555 | SJMS | SJMSG1 | Low | Low | NA | NA | NA |
| PRB09557 | SJMS | SJMSG1 | Low | Low | NA | NA | NA |
| PRB09559 | SJMS | SJMSG1 | Low | Low | NA | NA | NA |
| PRB09561 | SJMS | SJMSG1 | Low | Low | NA | NA | NA |
| PRB09563 | SJMS | SJMSG1 | Low | Low | NA | NA | NA |
| PRB09565 | SJMS | SJMSG1 | Low | Low | NA | NA | NA |
| PRB09567 | SJMS | SJMSG1 | Low | Low | NA | NA | NA |
| PRB09569 | SJMS | SJMSG1 | Low | Low | NA | NA | NA |
| PRB09571 | SJMS | SJMSG1 | Low | Low | Adult | Female | NA |
| PRB09573 | SJMS | SJMSG1 | Low | Low | NA | NA | NA |
| PRB09575 | SJMS | SJMSG1 | Low | Low | NA | NA | NA |
| PRB09577 | SJMS | SJMSG1 | Low | Low | NA | NA | NA |
| PRB09579 | SJMS | SJMSG1 | Low | Low | Young | Male | NA |
| PRB09581 | SJMS | SJMSG1 | Low | Low | NA | NA | NA |
| PRB09583 | SJMS | SJMSG1 | Low | Low | NA | NA | NA |
| PRB09585 | SJMS | SJMSG1 | Low | Low | NA | NA | NA |
| PRB09587 | SJMS | SJMSG1 | Low | Low | NA | NA | NA |
| PRB09589 | SJMS | SJMSG1 | Low | Low | NA | NA | NA |
| PRB09591 | SJMS | SJMSG1 | Low | Low | NA | NA | NA |
| PRB09593 | SJMS | SJMSG1 | Low | Low | NA | NA | NA |
| PRB09595 | SJMS | SJMSG1 | Low | Low | NA | NA | NA |
| PRB09597 | SJMS | SJMSG1 | Low | Low | Adult | Male | NA |
| PRB09599 | SJMS | SJMSG1 | Low | Low | NA | NA | NA |
| PRB09601 | SJMS | SJMSG1 | Low | Low | NA | NA | NA |
| PRB04297 | SPCB | SPCBG1 | Low | Low | Adult | NA | NA |
| PRB04299 | SPCB | SPCBG1 | Low | Low | Adult | Male | NA |
| PRB04301 | SPCB | SPCBG1 | Low | Low | Adult | NA | NA |
| PRB04303 | SPCB | SPCBG1 | Low | Low | Adult | Female | NA |
| PRB04305 | SPCB | SPCBG1 | Low | Low | Adult | NA | NA |
| PRB04307 | SPCB | SPCBG1 | Low | Low | Adult | Male | NA |
| PRB04309 | SPCB | SPCBG1 | Low | Low | NA | NA | NA |
| PRB04311 | SPCB | SPCBG1 | Low | Low | Young | NA | NA |
| PRB04313 | SPCB | SPCBG1 | Low | Low | Adult | Male | NA |
| PRB04315 | SPCB | SPCBG1 | Low | Low | Adult | NA | 0.6 |
| PRB04317 | SPCB | SPCBG1 | Low | Low | Young | Female | 3.4 |
| PRB04319 | SPCB | SPCBG1 | Low | Low | Adult | NA | 11.2 |
| PRB04321 | SPCB | SPCBG1 | Low | Low | Adult | NA | 1.6 |
| PRB04323 | SPCB | SPCBG1 | Low | Low | Adult | Female | 24.8 |
| PRB04325 | SPCB | SPCBG1 | Low | Low | Adult | NA | 3.7 |
| PRB04327 | SPCB | SPCBG1 | Low | Low | Adult | Female | 4.2 |
| PRB04329 | SPCB | SPCBG1 | Low | Low | Adult | Male | 16.9 |
| PRB04331 | SPCB | SPCBG1 | Low | Low | Adult | Male | 3 |
| PRB04333 | SPCB | SPCBG1 | Low | Low | Adult | NA | 5.5 |
| PRB04335 | SPCB | SPCBG1 | Low | Low | Adult | Female | 3.9 |
| PRB04337 | SPCB | SPCBG1 | Low | Low | Young | Male | 19.1 |
| PRB04339 | SPCB | SPCBG1 | Low | Low | Adult | NA | 16.9 |
| PRB04341 | SPCB | SPCBG1 | Low | Low | Adult | Female | 4.4 |
| PRB04343 | SPCB | SPCBG1 | Low | Low | Adult | Male | 1.9 |
| PRB04345 | SPCB | SPCBG1 | Low | Low | NA | NA | 6 |
| PRB04347 | SPCB | SPCBG1 | Low | Low | Adult | Female | 8.1 |
| PRB04349 | SPCB | SPCBG1 | Low | Low | Adult | NA | 18.8 |
| PRB04351 | SPCB | SPCBG1 | Low | Low | Adult | Female | 2.3 |
| PRB04353 | SPCB | SPCBG1 | Low | Low | Adult | Male | 1.7 |
| PRB04355 | SPCB | SPCBG1 | Low | Low | NA | NA | 9 |
| PRB04357 | SPCB | SPCBG1 | Low | Low | NA | NA | 0.9 |
| PRB04359 | SPCB | SPCBG1 | Low | Low | Adult | NA | 9.4 |
| PRB04361 | SPCB | SPCBG1 | Low | Low | Adult | NA | 1.4 |
| PRB04363 | SPCB | SPCBG1 | Low | Low | Adult | NA | 3.7 |
| PRB04365 | SPCB | SPCBG1 | Low | Low | Adult | NA | 5.8 |
| PRB04367 | SPCB | SPCBG1 | Low | Low | Adult | NA | 1.9 |
| PRB04369 | SPCB | SPCBG1 | Low | Low | Adult | NA | 21.8 |
| PRB04371 | SPCB | SPCBG1 | Low | Low | NA | NA | 4.3 |
| PRB04373 | SPCB | SPCBG1 | Low | Low | Adult | NA | 2.6 |
| PRB04375 | SPCB | SPCBG1 | Low | Low | NA | NA | NA |
| PRB04377 | SPCB | SPCBG1 | Low | Low | Adult | NA | 8.9 |
| PRB10313 | SPCB | SPCBG1 | Low | Low | Adult | Female | NA |
| PRB10315 | SPCB | SPCBG1 | Low | Low | Adult | Female | NA |
| PRB10317 | SPCB | SPCBG1 | Low | Low | Adult | Female | NA |
| PRB10319 | SPCB | SPCBG1 | Low | Low | Adult | Male | NA |
| PRB10321 | SPCB | SPCBG1 | Low | Low | Adult | Female | NA |
| PRB10323 | SPCB | SPCBG1 | Low | Low | Adult | Female | NA |
| PRB10325 | SPCB | SPCBG1 | Low | Low | Adult | Female | NA |
| PRB10327 | SPCB | SPCBG1 | Low | Low | Adult | Female | NA |
| PRB10329 | SPCB | SPCBG1 | Low | Low | Young | Male | NA |
| PRB10331 | SPCB | SPCBG1 | Low | Low | Adult | Male | NA |
| PRB10333 | SPCB | SPCBG1 | Low | Low | Adult | Female | NA |
| PRB10335 | SPCB | SPCBG1 | Low | Low | Adult | Female | NA |
| PRB10337 | SPCB | SPCBG1 | Low | Low | Adult | Female | NA |
| PRB10339 | SPCB | SPCBG1 | Low | Low | Young | Male | NA |
| PRB10341 | SPCB | SPCBG1 | Low | Low | Adult | Female | NA |
| PRB10343 | SPCB | SPCBG1 | Low | Low | Adult | Female | NA |
| PRB10345 | SPCB | SPCBG1 | Low | Low | Young | Male | NA |
| PRB10347 | SPCB | SPCBG1 | Low | Low | Adult | Female | NA |
| PRB10349 | SPCB | SPCBG1 | Low | Low | Adult | Male | NA |
| PRB10351 | SPCB | SPCBG1 | Low | Low | Adult | Male | NA |
| PRB04431 | SPCB | SPCBG2 | Low | Low | Adult | Female | NA |
| PRB04433 | SPCB | SPCBG2 | Low | Low | Adult | Female | NA |
| PRB04435 | SPCB | SPCBG2 | Low | Low | Adult | Male | 3.2 |
| PRB04437 | SPCB | SPCBG2 | Low | Low | NA | NA | 3.6 |
| PRB04439 | SPCB | SPCBG2 | Low | Low | Adult | Male | 2.2 |
| PRB04441 | SPCB | SPCBG2 | Low | Low | Adult | Male | 0.1 |
| PRB04443 | SPCB | SPCBG2 | Low | Low | Young | Female | 4.3 |
| PRB04445 | SPCB | SPCBG2 | Low | Low | NA | NA | 6.6 |
| PRB04447 | SPCB | SPCBG2 | Low | Low | NA | NA | 0.9 |
| PRB04449 | SPCB | SPCBG2 | Low | Low | NA | NA | NA |
| PRB04451 | SPCB | SPCBG2 | Low | Low | NA | NA | 16.1 |
| PRB04453 | SPCB | SPCBG2 | Low | Low | Adult | Female | 11.5 |
| PRB04455 | SPCB | SPCBG2 | Low | Low | Adult | Female | 3.6 |
| PRB04457 | SPCB | SPCBG2 | Low | Low | Young | Male | NA |
| PRB04459 | SPCB | SPCBG2 | Low | Low | Adult | Female | 6.1 |
| PRB04461 | SPCB | SPCBG2 | Low | Low | Adult | Male | 11.3 |
| PRB04463 | SPCB | SPCBG2 | Low | Low | Adult | Female | 2.5 |
| PRB04465 | SPCB | SPCBG2 | Low | Low | Adult | Female | 2.4 |
| PRB04467 | SPCB | SPCBG2 | Low | Low | Adult | Male | 3.4 |
| PRB04469 | SPCB | SPCBG2 | Low | Low | NA | NA | 6.5 |
| PRB04471 | SPCB | SPCBG2 | Low | Low | NA | NA | 4.2 |
| PRB04473 | SPCB | SPCBG2 | Low | Low | NA | NA | 5.1 |
| PRB04475 | SPCB | SPCBG2 | Low | Low | Adult | Female | 2.2 |
| PRB04477 | SPCB | SPCBG2 | Low | Low | Adult | Male | NA |
| PRB04479 | SPCB | SPCBG2 | Low | Low | Adult | Male | NA |
| PRB04481 | SPCB | SPCBG2 | Low | Low | Adult | Male | 2.5 |
| PRB04483 | SPCB | SPCBG2 | Low | Low | Adult | Female | 8.2 |
| PRB04485 | SPCB | SPCBG2 | Low | Low | NA | NA | 7 |
| PRB04487 | SPCB | SPCBG2 | Low | Low | Adult | Female | 3.9 |
| PRB04489 | SPCB | SPCBG2 | Low | Low | Adult | Female | 8 |
| PRB04491 | SPCB | SPCBG2 | Low | Low | NA | NA | 12 |
| PRB04493 | SPCB | SPCBG2 | Low | Low | Adult | Female | 1.2 |
| PRB04495 | SPCB | SPCBG2 | Low | Low | NA | NA | 3.6 |
| PRB04497 | SPCB | SPCBG2 | Low | Low | Adult | NA | 2.7 |
| PRB04499 | SPCB | SPCBG2 | Low | Low | Young | NA | 2.2 |
| PRB04501 | SPCB | SPCBG2 | Low | Low | Adult | Female | 6.2 |
| PRB10353 | SPCB | SPCBG2 | Low | Low | Adult | Male | NA |
| PRB10355 | SPCB | SPCBG2 | Low | Low | Young | Female | NA |
| PRB10357 | SPCB | SPCBG2 | Low | Low | Adult | Male | NA |
| PRB10359 | SPCB | SPCBG2 | Low | Low | Adult | Female | NA |
| PRB10361 | SPCB | SPCBG2 | Low | Low | Adult | Male | NA |
| PRB10363 | SPCB | SPCBG2 | Low | Low | Adult | Male | NA |
| PRB10365 | SPCB | SPCBG2 | Low | Low | Adult | Female | NA |
| PRB10367 | SPCB | SPCBG2 | Low | Low | Adult | Male | NA |
| PRB10369 | SPCB | SPCBG2 | Low | Low | Adult | Female | NA |
| PRB10371 | SPCB | SPCBG2 | Low | Low | Young | Male | NA |
| PRB10373 | SPCB | SPCBG2 | Low | Low | Adult | Female | NA |
| PRB10375 | SPCB | SPCBG2 | Low | Low | Adult | Male | NA |
| PRB10377 | SPCB | SPCBG2 | Low | Low | Adult | Female | NA |
| PRB10379 | SPCB | SPCBG2 | Low | Low | Adult | Male | NA |
| PRB10381 | SPCB | SPCBG2 | Low | Low | Adult | Male | NA |
| PRB10383 | SPCB | SPCBG2 | Low | Low | Adult | Male | NA |
| PRB10385 | SPCB | SPCBG2 | Low | Low | Adult | Female | NA |
| PRB10387 | SPCB | SPCBG2 | Low | Low | Young | Male | NA |
| PRB10389 | SPCB | SPCBG2 | Low | Low | Adult | Male | NA |
| PRB10391 | SPCB | SPCBG2 | Low | Low | Adult | Female | NA |
| PRB10393 | SPCB | SPCBG2 | Low | Low | Young | Female | NA |
| PRB10395 | SPCB | SPCBG2 | Low | Low | Adult | Male | NA |
| PRB10397 | SPCB | SPCBG2 | Low | Low | Adult | Male | NA |
| PRB10399 | SPCB | SPCBG2 | Low | Low | Adult | Male | NA |
| PRB10401 | SPCB | SPCBG2 | Low | Low | Adult | Female | NA |
| PRB10403 | SPCB | SPCBG2 | Low | Low | Adult | Female | NA |
| PRB10405 | SPCB | SPCBG2 | Low | Low | Adult | Female | NA |
| PRB10407 | SPCB | SPCBG2 | Low | Low | Adult | Male | NA |
| PRB10409 | SPCB | SPCBG2 | Low | Low | Adult | Male | NA |
| PRB10411 | SPCB | SPCBG2 | Low | Low | Adult | Male | NA |
| PRB10413 | SPCB | SPCBG2 | Low | Low | Adult | Male | NA |
| PRB10415 | SPCB | SPCBG2 | Low | Low | Adult | Male | NA |
| PRB10417 | SPCB | SPCBG2 | Low | Low | Adult | Female | NA |
| PRB10419 | SPCB | SPCBG2 | Low | Low | Adult | Female | NA |
| PRB10421 | SPCB | SPCBG2 | Low | Low | Adult | Male | NA |
| PRB10423 | SPCB | SPCBG2 | Low | Low | Adult | Male | NA |
| PRB10425 | SPCB | SPCBG2 | Low | Low | Adult | Female | NA |
| PRB10427 | SPCB | SPCBG2 | Low | Low | Adult | Female | NA |
| PRB10429 | SPCB | SPCBG2 | Low | Low | Adult | Female | NA |
| PRB10431 | SPCB | SPCBG2 | Low | Low | Young | Male | NA |
| PRB10433 | SPCB | SPCBG2 | Low | Low | Adult | Male | NA |
| PRB10435 | SPCB | SPCBG2 | Low | Low | Adult | Male | NA |
| PRB10437 | SPCB | SPCBG2 | Low | Low | Adult | Male | NA |
| PRB10439 | SPCB | SPCBG2 | Low | Low | Adult | Female | NA |
| PRB10441 | SPCB | SPCBG2 | Low | Low | Young | Male | NA |
| PRB10443 | SPCB | SPCBG2 | Low | Low | Adult | Female | NA |
| PRB10445 | SPCB | SPCBG2 | Low | Low | Adult | Female | NA |
| PRB10447 | SPCB | SPCBG2 | Low | Low | Adult | Male | NA |
| PRB10449 | SPCB | SPCBG2 | Low | Low | Young | Male | NA |
| PRB10451 | SPCB | SPCBG2 | Low | Low | Adult | Male | NA |
| PRB10453 | SPCB | SPCBG2 | Low | Low | Adult | Male | NA |
| PRB10455 | SPCB | SPCBG2 | Low | Low | Adult | Male | NA |
| PRB10457 | SPCB | SPCBG2 | Low | Low | Adult | Female | NA |
| PRB10459 | SPCB | SPCBG2 | Low | Low | Young | Female | NA |
| PRB10461 | SPCB | SPCBG2 | Low | Low | Adult | Male | NA |
| PRB10221 | SPCB | SPCBG3 | Low | Low | Adult | Male | NA |
| PRB10223 | SPCB | SPCBG3 | Low | Low | Adult | Female | NA |
| PRB10225 | SPCB | SPCBG3 | Low | Low | Adult | Female | NA |
| PRB10227 | SPCB | SPCBG3 | Low | Low | Adult | Female | NA |
| PRB10229 | SPCB | SPCBG3 | Low | Low | Adult | Male | NA |
| PRB10231 | SPCB | SPCBG3 | Low | Low | Adult | Female | NA |
| PRB10233 | SPCB | SPCBG3 | Low | Low | Adult | Male | NA |
| PRB10235 | SPCB | SPCBG3 | Low | Low | Young | Male | NA |
| PRB10237 | SPCB | SPCBG3 | Low | Low | Adult | Male | NA |
| PRB10239 | SPCB | SPCBG3 | Low | Low | Adult | Female | NA |
| PRB10241 | SPCB | SPCBG3 | Low | Low | Young | Female | NA |
| PRB10243 | SPCB | SPCBG3 | Low | Low | Adult | Male | NA |
| PRB10245 | SPCB | SPCBG3 | Low | Low | Adult | Male | NA |
| PRB10247 | SPCB | SPCBG3 | Low | Low | Adult | Female | NA |
| PRB10249 | SPCB | SPCBG3 | Low | Low | Adult | Male | NA |
| PRB10251 | SPCB | SPCBG3 | Low | Low | Adult | Female | NA |
| PRB10253 | SPCB | SPCBG3 | Low | Low | Adult | Male | NA |
| PRB10255 | SPCB | SPCBG3 | Low | Low | Young | Female | NA |
| PRB10257 | SPCB | SPCBG3 | Low | Low | Adult | Female | NA |
| PRB10259 | SPCB | SPCBG3 | Low | Low | Adult | Male | NA |
| PRB10261 | SPCB | SPCBG3 | Low | Low | Adult | Female | NA |
| PRB10263 | SPCB | SPCBG3 | Low | Low | Adult | Female | NA |
| PRB10265 | SPCB | SPCBG3 | Low | Low | Adult | Female | NA |
| PRB10267 | SPCB | SPCBG3 | Low | Low | Adult | Male | NA |
| PRB10269 | SPCB | SPCBG3 | Low | Low | Adult | Male | NA |
| PRB10271 | SPCB | SPCBG3 | Low | Low | Adult | Male | NA |
| PRB10273 | SPCB | SPCBG3 | Low | Low | Adult | Male | NA |
| PRB10275 | SPCB | SPCBG3 | Low | Low | Young | Female | NA |
| PRB10277 | SPCB | SPCBG3 | Low | Low | Adult | Female | NA |
| PRB10279 | SPCB | SPCBG3 | Low | Low | Young | Female | NA |
| PRB10281 | SPCB | SPCBG3 | Low | Low | Adult | Male | NA |
| PRB10283 | SPCB | SPCBG3 | Low | Low | Young | Female | NA |
| PRB10285 | SPCB | SPCBG3 | Low | Low | Adult | Female | NA |
| PRB10287 | SPCB | SPCBG3 | Low | Low | Adult | Male | NA |
| PRB10289 | SPCB | SPCBG3 | Low | Low | Adult | Male | NA |
| PRB10291 | SPCB | SPCBG3 | Low | Low | Adult | Female | NA |
| PRB10293 | SPCB | SPCBG3 | Low | Low | Adult | Female | NA |
| PRB10295 | SPCB | SPCBG3 | Low | Low | Adult | Female | NA |
| PRB10297 | SPCB | SPCBG3 | Low | Low | Adult | Male | NA |
| PRB03447 | WB | WBG1 | Medium | High | Adult | Male | 3 |
| PRB03449 | WB | WBG1 | Medium | High | NA | NA | 6.8 |
| PRB03451 | WB | WBG1 | Medium | High | NA | NA | 1.1 |
| PRB03453 | WB | WBG1 | Medium | High | Adult | Male | 6 |
| PRB03455 | WB | WBG1 | Medium | High | NA | NA | 0.4 |
| PRB03457 | WB | WBG1 | Medium | High | Adult | Male | 0 |
| PRB03459 | WB | WBG1 | Medium | High | Young | Female | 0.1 |
| PRB03461 | WB | WBG1 | Medium | High | Adult | Female | 0 |
| PRB03463 | WB | WBG1 | Medium | High | Adult | Female | 12.1 |
| PRB03465 | WB | WBG1 | Medium | High | NA | Female | 21.5 |
| PRB08393 | WB | WBG1 | Medium | High | NA | NA | NA |
| PRB08395 | WB | WBG1 | Medium | High | NA | NA | NA |
| PRB08397 | WB | WBG1 | Medium | High | NA | NA | NA |
| PRB08399 | WB | WBG1 | Medium | High | NA | NA | NA |
| PRB08401 | WB | WBG1 | Medium | High | NA | NA | NA |
| PRB08403 | WB | WBG1 | Medium | High | NA | NA | NA |
| PRB08405 | WB | WBG1 | Medium | High | NA | NA | NA |
| PRB08407 | WB | WBG1 | Medium | High | NA | NA | NA |
| PRB08409 | WB | WBG1 | Medium | High | NA | NA | NA |
| PRB08411 | WB | WBG1 | Medium | High | NA | NA | NA |
| PRB08413 | WB | WBG1 | Medium | High | NA | NA | NA |
| PRB08415 | WB | WBG1 | Medium | High | NA | NA | NA |
| PRB08417 | WB | WBG1 | Medium | High | NA | NA | NA |
| PRB08419 | WB | WBG1 | Medium | High | NA | NA | NA |
| PRB08421 | WB | WBG1 | Medium | High | Young | Female | NA |
| PRB08423 | WB | WBG1 | Medium | High | NA | NA | NA |
| PRB08425 | WB | WBG1 | Medium | High | NA | NA | NA |
| PRB08427 | WB | WBG1 | Medium | High | NA | NA | NA |
| PRB08429 | WB | WBG1 | Medium | High | NA | NA | NA |
| PRB08431 | WB | WBG1 | Medium | High | NA | NA | NA |
| PRB08433 | WB | WBG1 | Medium | High | NA | NA | NA |
| PRB08435 | WB | WBG1 | Medium | High | NA | NA | NA |
| PRB08437 | WB | WBG1 | Medium | High | NA | NA | NA |
| PRB08439 | WB | WBG1 | Medium | High | NA | NA | NA |
| PRB08519 | WB | WBG1 | Medium | High | NA | NA | NA |
| PRB08521 | WB | WBG1 | Medium | High | NA | NA | NA |
| PRB08523 | WB | WBG1 | Medium | High | NA | NA | NA |
| PRB08525 | WB | WBG1 | Medium | High | NA | NA | NA |
| PRB08527 | WB | WBG1 | Medium | High | NA | NA | NA |
| PRB08529 | WB | WBG1 | Medium | High | NA | NA | NA |
| PRB08531 | WB | WBG1 | Medium | High | NA | NA | NA |
| PRB08533 | WB | WBG1 | Medium | High | NA | NA | NA |
| PRB08535 | WB | WBG1 | Medium | High | NA | NA | NA |
| PRB08537 | WB | WBG1 | Medium | High | NA | NA | NA |
| PRB08539 | WB | WBG1 | Medium | High | NA | NA | NA |
| PRB08541 | WB | WBG1 | Medium | High | NA | NA | NA |
| PRB08543 | WB | WBG1 | Medium | High | NA | NA | NA |
| PRB08545 | WB | WBG1 | Medium | High | Adult | Male | NA |
| PRB08547 | WB | WBG1 | Medium | High | NA | NA | NA |
| PRB08549 | WB | WBG1 | Medium | High | NA | NA | NA |
| PRB08551 | WB | WBG1 | Medium | High | NA | NA | NA |
| PRB08553 | WB | WBG1 | Medium | High | NA | NA | NA |
| PRB08555 | WB | WBG1 | Medium | High | NA | NA | NA |
| PRB08557 | WB | WBG1 | Medium | High | NA | NA | NA |
| PRB08559 | WB | WBG1 | Medium | High | NA | NA | NA |
| PRB08561 | WB | WBG1 | Medium | High | NA | NA | NA |
| PRB08563 | WB | WBG1 | Medium | High | NA | NA | NA |
| PRB08565 | WB | WBG1 | Medium | High | NA | NA | NA |
| PRB08567 | WB | WBG1 | Medium | High | NA | NA | NA |
| PRB08569 | WB | WBG1 | Medium | High | NA | NA | NA |
| PRB08571 | WB | WBG1 | Medium | High | NA | NA | NA |
| PRB08573 | WB | WBG1 | Medium | High | NA | NA | NA |
| PRB03467 | WB | WBG2 | Medium | High | NA | NA | 6.4 |
| PRB03469 | WB | WBG2 | Medium | High | NA | NA | 0 |
| PRB03471 | WB | WBG2 | Medium | High | NA | NA | 1 |
| PRB03473 | WB | WBG2 | Medium | High | NA | NA | 9.7 |
| PRB03475 | WB | WBG2 | Medium | High | NA | NA | 53.2 |
| PRB03477 | WB | WBG2 | Medium | High | NA | NA | 36.5 |
| PRB03479 | WB | WBG2 | Medium | High | NA | NA | 0 |
| PRB03481 | WB | WBG2 | Medium | High | NA | NA | 3.5 |
| PRB03483 | WB | WBG2 | Medium | High | NA | NA | 5.5 |
| PRB03485 | WB | WBG2 | Medium | High | NA | NA | 4.4 |
| PRB03487 | WB | WBG2 | Medium | High | NA | NA | 4.9 |
| PRB03489 | WB | WBG2 | Medium | High | NA | NA | 0 |
| PRB03491 | WB | WBG2 | Medium | High | Young | Female | 0 |
| PRB03493 | WB | WBG2 | Medium | High | NA | NA | 0 |
| PRB03495 | WB | WBG2 | Medium | High | NA | NA | 4 |
| PRB03497 | WB | WBG2 | Medium | High | NA | NA | 2.9 |
| PRB03499 | WB | WBG2 | Medium | High | Adult | Male | 20.8 |
| PRB03501 | WB | WBG2 | Medium | High | NA | NA | 9.2 |
| PRB03503 | WB | WBG2 | Medium | High | NA | NA | 0 |
| PRB03505 | WB | WBG2 | Medium | High | NA | NA | NA |
| PRB03507 | WB | WBG2 | Medium | High | NA | NA | 1.4 |
| PRB03509 | WB | WBG2 | Medium | High | NA | NA | 0 |
| PRB03511 | WB | WBG2 | Medium | High | NA | NA | 0 |
| PRB03513 | WB | WBG2 | Medium | High | NA | NA | 6.8 |
| PRB08441 | WB | WBG2 | Medium | High | NA | NA | NA |
| PRB08443 | WB | WBG2 | Medium | High | NA | NA | NA |
| PRB08445 | WB | WBG2 | Medium | High | NA | NA | NA |
| PRB08447 | WB | WBG2 | Medium | High | NA | NA | NA |
| PRB08449 | WB | WBG2 | Medium | High | NA | NA | NA |
| PRB08451 | WB | WBG2 | Medium | High | NA | NA | NA |
| PRB08453 | WB | WBG2 | Medium | High | NA | NA | NA |
| PRB08455 | WB | WBG2 | Medium | High | NA | NA | NA |
| PRB08457 | WB | WBG2 | Medium | High | NA | NA | NA |
| PRB08459 | WB | WBG2 | Medium | High | NA | NA | NA |
| PRB08461 | WB | WBG2 | Medium | High | NA | NA | NA |
| PRB08463 | WB | WBG2 | Medium | High | NA | NA | NA |
| PRB08465 | WB | WBG2 | Medium | High | NA | NA | NA |
| PRB08467 | WB | WBG2 | Medium | High | Adult | Female | NA |
| PRB08469 | WB | WBG2 | Medium | High | NA | NA | NA |
| PRB08471 | WB | WBG2 | Medium | High | NA | NA | NA |
| PRB08473 | WB | WBG2 | Medium | High | NA | NA | NA |
| PRB08475 | WB | WBG2 | Medium | High | NA | NA | NA |
| PRB08477 | WB | WBG2 | Medium | High | NA | NA | NA |
| PRB08479 | WB | WBG2 | Medium | High | NA | NA | NA |
| PRB08481 | WB | WBG2 | Medium | High | NA | NA | NA |
| PRB08483 | WB | WBG2 | Medium | High | NA | NA | NA |
| PRB08485 | WB | WBG2 | Medium | High | NA | NA | NA |
| PRB08487 | WB | WBG2 | Medium | High | NA | NA | NA |
| PRB08489 | WB | WBG2 | Medium | High | NA | NA | NA |
| PRB08491 | WB | WBG2 | Medium | High | NA | NA | NA |
| PRB08493 | WB | WBG2 | Medium | High | NA | NA | NA |
| PRB08495 | WB | WBG2 | Medium | High | NA | NA | NA |
| PRB08497 | WB | WBG2 | Medium | High | NA | NA | NA |
| PRB08499 | WB | WBG2 | Medium | High | NA | NA | NA |
| PRB08501 | WB | WBG2 | Medium | High | NA | NA | NA |
| PRB08503 | WB | WBG2 | Medium | High | NA | NA | NA |
| PRB08505 | WB | WBG2 | Medium | High | NA | NA | NA |
| PRB08507 | WB | WBG2 | Medium | High | NA | NA | NA |
| PRB08509 | WB | WBG2 | Medium | High | NA | NA | NA |
| PRB08511 | WB | WBG2 | Medium | High | NA | NA | NA |
| PRB08513 | WB | WBG2 | Medium | High | NA | NA | NA |
| PRB08515 | WB | WBG2 | Medium | High | NA | NA | NA |
| PRB08517 | WB | WBG2 | Medium | High | NA | NA | NA |
| PRB03515 | WB | WBG3 | Medium | High | NA | NA | 66.7 |
| PRB03517 | WB | WBG3 | Medium | High | NA | NA | 18.1 |
| PRB03519 | WB | WBG3 | Medium | High | NA | NA | 2.9 |
| PRB03521 | WB | WBG3 | Medium | High | NA | NA | 2.2 |
| PRB03523 | WB | WBG3 | Medium | High | NA | NA | 1 |
| PRB03525 | WB | WBG3 | Medium | High | NA | NA | 2.4 |
| PRB03527 | WB | WBG3 | Medium | High | NA | NA | 0 |
| PRB03529 | WB | WBG3 | Medium | High | NA | NA | 5.1 |
| PRB03531 | WB | WBG3 | Medium | High | NA | NA | 2.4 |
| PRB03533 | WB | WBG3 | Medium | High | NA | Male | 0 |
| PRB03535 | WB | WBG3 | Medium | High | NA | NA | 0 |
| PRB03537 | WB | WBG3 | Medium | High | NA | Male | 1.8 |
| PRB03539 | WB | WBG3 | Medium | High | NA | NA | 4 |
| PRB03541 | WB | WBG3 | Medium | High | Adult | Female | 3.5 |
| PRB03543 | WB | WBG3 | Medium | High | NA | NA | 0.2 |
| PRB08575 | WB | WBG3 | Medium | High | NA | NA | NA |
| PRB08577 | WB | WBG3 | Medium | High | NA | NA | NA |
| PRB08579 | WB | WBG3 | Medium | High | NA | NA | NA |
| PRB08581 | WB | WBG3 | Medium | High | NA | NA | NA |
| PRB08583 | WB | WBG3 | Medium | High | NA | NA | NA |
| PRB08585 | WB | WBG3 | Medium | High | NA | NA | NA |
| PRB08587 | WB | WBG3 | Medium | High | NA | NA | NA |
| PRB08589 | WB | WBG3 | Medium | High | NA | NA | NA |
| PRB08591 | WB | WBG3 | Medium | High | NA | NA | NA |
| PRB08593 | WB | WBG3 | Medium | High | NA | NA | NA |
| PRB08595 | WB | WBG3 | Medium | High | NA | NA | NA |
| PRB08597 | WB | WBG3 | Medium | High | NA | NA | NA |
| PRB08599 | WB | WBG3 | Medium | High | NA | NA | NA |
| PRB08601 | WB | WBG3 | Medium | High | NA | NA | NA |
| PRB08603 | WB | WBG3 | Medium | High | NA | NA | NA |
| PRB08605 | WB | WBG3 | Medium | High | NA | NA | NA |
| PRB08607 | WB | WBG3 | Medium | High | NA | NA | NA |
| PRB08609 | WB | WBG3 | Medium | High | NA | NA | NA |
| PRB08611 | WB | WBG3 | Medium | High | NA | NA | NA |
| PRB08613 | WB | WBG3 | Medium | High | NA | NA | NA |
| PRB08615 | WB | WBG3 | Medium | High | NA | NA | NA |
| PRB08617 | WB | WBG3 | Medium | High | NA | NA | NA |
| PRB08619 | WB | WBG4 | Medium | High | NA | NA | NA |
| PRB08621 | WB | WBG4 | Medium | High | NA | NA | NA |
| PRB08623 | WB | WBG4 | Medium | High | NA | NA | NA |
| PRB08625 | WB | WBG4 | Medium | High | NA | NA | NA |
| PRB08627 | WB | WBG4 | Medium | High | NA | NA | NA |
| PRB08629 | WB | WBG4 | Medium | High | NA | NA | NA |
| PRB08631 | WB | WBG4 | Medium | High | NA | NA | NA |
| PRB08633 | WB | WBG4 | Medium | High | NA | NA | NA |
| PRB08635 | WB | WBG4 | Medium | High | NA | NA | NA |
| PRB08637 | WB | WBG4 | Medium | High | NA | NA | NA |
| PRB08639 | WB | WBG4 | Medium | High | NA | NA | NA |
| PRB08641 | WB | WBG4 | Medium | High | NA | NA | NA |
| PRB08643 | WB | WBG4 | Medium | High | NA | NA | NA |
| PRB08645 | WB | WBG4 | Medium | High | NA | NA | NA |
| PRB08647 | WB | WBG4 | Medium | High | NA | NA | NA |
| PRB08649 | WB | WBG4 | Medium | High | NA | NA | NA |
| PRB08651 | WB | WBG4 | Medium | High | NA | NA | NA |
| PRB08653 | WB | WBG4 | Medium | High | NA | NA | NA |
| PRB08655 | WB | WBG4 | Medium | High | NA | NA | NA |
| PRB08657 | WB | WBG4 | Medium | High | NA | NA | NA |
| PRB08659 | WB | WBG4 | Medium | High | NA | NA | NA |

**Supporting Figure 1: Macaque baiting in urban area (Dhaka) by one of the in-country field team members**

**Supporting Figure 2: Work flow and molecular results at each stage of sample processing.**

**Supporting Figure 3: Percent Identity Distance (PID) histogram showing distribution of pairwise sequence identities for all 11,303 PbV sequences. X axis is the percent identity between pairwise sequences. Y axis is the frequency of occurrence. Several clear peaks and throughs were identified including troughs at 96% and 88% that served as genetic cut-off values to demarcate OTUs**

**Supporting Figure 4**: An example of an OTU generated using a genetic cut-off of ≥96%, informed by the PID histogram. A star-like radial phylogeny is observed with no evidence of sub-clustering.

**Supporting Figure 5**: An example of an OTU generated using a genetic cut-off of ≥88%, informed by the PID histogram. Evidence of phylogenetic sub-clustering is observed, potentially indicating discrete evolutionary processes shaping these sequences

**Supporting Table 3: Summary of network measures and pre-network randomization tests of viral OTU co-occurrence networks, when OTUs were assigned using a criterion of 88% genetic similarity.**

|  | Individual | Group | Site |
| --- | --- | --- | --- |
| Modularity | 0.62** | 0.55** | 0.54** |
| Number of communities | 27** | 8** | 6** |

**p < 0.01

**Supporting Figure 6: Plots of variance partitioning of the effects of animal and environmental co-variates on viral OTU (assignments based on a criterion <88% phylogenetic similarity) assemblages, from Joint Species Distribution Models (JSDMs) of (A) individual macaques within groups, and (B) macaque groups within species.**

**
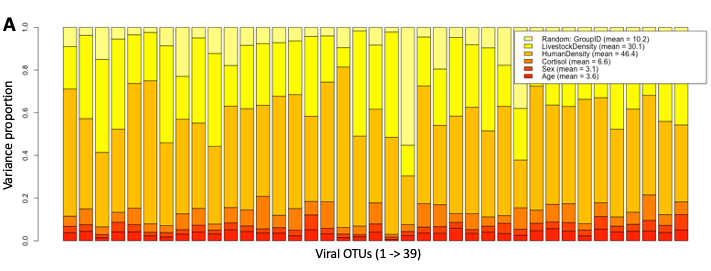
**

**Supporting Figure 7: Heat-maps of posterior support values from the Joint Species Distribution Models (JSDMs) examining the effects of animal and environmental co-variates on viral OTU (assignments based on a criterion <88% phylogenetic similarity) assemblages, at (A) the individual level with group as a random effect, and (B) the group level with site as a random effect. Rows contain the co-variates, and the columns viral OTUs whose occurrence they predict. Cells indicate strongly positive (red) and strongly negative (blue) effects at >95% posterior support. Blank or white cells indicate no strong effects.**

**
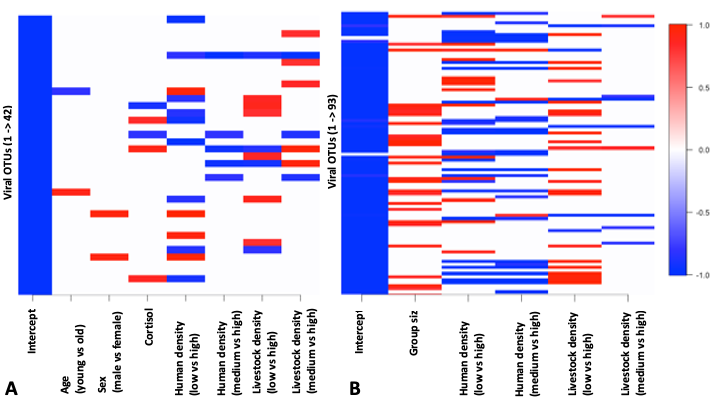
**

**Supporting Figure 8: Heat-maps that indicate residual correlations between viral OTU (assignments based on a criterion >88% phylogenetic similarity) pairs for individual-level JSDMs with group as a random effect, and for group-level JSDMs with site as a random effect, for posterior support cut-offs of (A) 75% and (B) 95%. Cells indicate both strongly positive (red) and strongly negative (blue) residual correlations between OTU pairs after accounting for phylogenetic relationships, and for animal and environmental co-variates. White cells indicate no strong correlations. In each heat-map, OTUs have been re-ordered to best illustrate the observed association structure. (C) Bar-plots and proportions-test results comparing the percentages of viral OTU pairs that showed strong (both positive and negative) residual correlations across the organizational levels, i.e. individuals within groups, versus groups within sites**.

**References:**

Anthony, S. J., Islam, A., Johnson, C., Navarrete-Macias, I., Liang, E., Jain, K., Hitchens, P. L., Che, X., Soloyvov, A., Hicks, A. L., Ojeda-Flores, R.,Zambrana-Torrelio, C., Ulrich, W., Rostal, M. K., Petrosov, A., Garcia, J., Haider, N., Wolfe, N., Goldstein, T., Morse, S. S., Rahman, M., Epstein, J. H., Mazet, J., Daszak, P., & Lipkin, W. I. (2015). Non-random patterns in viral diversity. *Nature Communications*, *6*, 8147.

Engel, G. A., & Besnard, F. (2017). Human – Nonhuman Primate Disease Transmission. In *The International Encyclopedia of Primatology*. <https://doi.org/10.1002/9781119179313.wbprim0374>

Islam, S., Rahman, M. K., Uddin, M. H., Rahman, M. M., Chowdhury, M. N. U., Hassan, M. M., Magalhaes, R. S., & Islam, A. (2022). Prevalence and diversity of gastrointestinal parasites in free-ranging rhesus macaques (*Macaca mulatta*) in different land gradients of Bangladesh. *American Journal of Primatology*, 84:e23345

Oikarinen, S., Tauriainen, S., Viskari, H., Simell, O., Knip, M., Virtanen, S., & Hyöty, H. (2009). PCR inhibition in stool samples in relation to age of infants. *Journal of Clinical Virology*, *44*(3), 211–214.

Padaris, E., & Schliep, K. (2019). Ape 5.0: an environment for modern phylogenetics and evolutionary analysis in R. *Bioinformatics*, 35, 526-528

Rawlins, R. G. & Kessler, M. J. (1986). The cayo santiago macaques history, behavior, and biology, 1st edition. State University of New York Press

Wolfe, N. D., Escalante, A. A., Karesh, W. B., Kilbourn, A., Spielman, A. & Lal, A. A. (1998). Wild primate populations in emerging infectious disease research: The missing link? *Emerging Infectious Diseases*, *4*, 149–158.

Wolfe, N. D., Dunavan, C. P., & Diamond, J. (2007). Origins of major human infectious diseases. *Nature*, *447*(7142), 279–283.
